# Cost analysis of implementing a vial-sharing strategy for chemotherapy drugs using intelligent dispensing robots in a tertiary Chinese hospital in Sichuan

**DOI:** 10.3389/fpubh.2022.936686

**Published:** 2022-09-21

**Authors:** Hui Liu, Linke Zou, Yujie Song, Junfeng Yan

**Affiliations:** ^1^Department of Pharmacy, Sichuan Academy of Medical Sciences and Sichuan Provincial People's Hospital, School of Medicine, University of Electronic Science and Technology of China, Chengdu, China; ^2^Personalized Drug Therapy Key Laboratory of Sichuan Province, Chengdu, China

**Keywords:** chemotherapy drug, amount of waste, cost savings, vial-sharing, intelligent dispensing robots

## Abstract

**Introduction:**

Chemotherapy drug wasting is a huge problem in oncology that not only results in excessive expenses on chemotherapy drugs but also increases the cost of disposing of chemotherapy waste and the risk of occupational exposure in the environment. The main objective of this study was to evaluate the potential for hospitals in China to employ a real-time vial-sharing strategy that can save drug costs.

**Method:**

This study was conducted retrospectively at Pharmacy Intravenous Admixture Services (PIVAS), People's Hospital of Sichuan Province, China, from September to November 2021. Data on prescription drugs wasted were collected from the Hospital Information System (HIS). To assess the real-time vial-sharing strategy, we estimated drug wastage and drug waste costs using intelligent robots that dispense multiple prescriptions simultaneously.

**Results:**

24 of the 46 wasted drugs were cost-saved. The vial-sharing strategy saved 186,067 mg of drugs, or ~59.08% of the total amount wasted, resulting in savings of 150,073.53 China Yuan (CNY), or 47.51% of the cost of the total waste.

**Conclusion:**

Our investigation established that employing a real-time vial-sharing strategy using an intelligent robot to dispense multiple prescriptions simultaneously is cost-effective. Additionally, this approach presented no safety issue concerns, such as the introduction of impurities to sterile compounding *via* repeated interspersing or the incorrect registration of information during drug storage, often encountered with traditional vial-sharing strategies.

## Introduction

According to a recent statistical finding, the number of new tumor cases worldwide reached 19.3 million in 2020 (1). Cancer is among the leading causes of human death. Globally, the cost of cancer treatment is usually very high because treatment is long-term and drugs are very expensive, and providing affordable health care becomes a huge challenge. Increasing health care costs are putting a huge strain on patients and the health insurance system. The drastic increase in the use of chemotherapy drugs has led to an upsurge in the amounts of intravenous chemotherapy drugs left in vials after use due to individualized dosing, resulting in significant drug waste. From an economic point of view, the value of drugs discarded as waste is high, and disposing of the waste is equally expensive. Therefore, reducing drug waste in this part of oncology is essential for resource and cost savings.

Several studies have assessed the amount and cost of wasted chemotherapy drugs around the world (2–6). Investigations revealed that patients incurred vast drug waste-related economic consequences because they paid for both the dosages used and discarded. Ibrahim suggested that dose rounding of chemotherapy drugs could result in theoretical cost savings of about 10%, with potential annual cost savings of $192,800 (7). Vandyke et al. (8) made cost savings of nearly $200,000 in 1 year through automated dose rounding administered by pharmacists. Heinhuis et al.'s (9) implementation of fixed-dosing led to a significant reduction in the number of vials used for almost all monoclonal antibodies. Measures such as dose-banding and fixed-dosing also increased the possibility of recycling unused drugs during their expiration date. In the US study (10), Bendamustine and Bortezomib, were used as examples to assess the impact of different packaging methods on single-dose vials, and the results showed that drug waste can be significantly reduced by optimizing vials. Unfortunately, implementing this method is beyond the control of medical institutions because designing vial specifications mainly depends on pharmaceutical enterprises. Jiang et al. (11) obtained a 394,536 CAD (21.1%) reduction in total drug costs over 3 years by scheduling as many patients to receive carbazole on 1 weekday as possible for combination chemotherapy. To accommodate vial sharing, some studies have also used Closed System Drug Transfer Devices (CSTD) to extend the shelf life of drugs after opening. Edwards et al. (12) saved over $96,000 over 7 weeks using CSTD, with an estimated $700,000 saved per year. Juhász et al. (13) achieved cost savings of up to 18.6% using CSTD for expensive intravenous biologics.

Based on literature studies, few investigations on the real-time vial-sharing strategy have been conducted outside of China. Few inquiries on chemotherapy drug waste and vial sharing are currently available in China. Vial-sharing in previous studies usually meant preparing each prescription drug individually and retaining the remainder of the current vial for reuse when preparing the same drug for the next prescription (12–18). There is a need to evaluate the sterile state and stability data of the product and a need to amplify efforts to preserve residual drugs, which is obviously very complex and easy to make mistakes. However, the process of preparation in our current research is based on the need to use the same drug for different prescriptions, with the intelligent dispensing robot employed to prepare all prescription doses of the same drug at once.

The objectives of this study are:

1. To determine the extent of chemotherapy drug wastage and its cost in a tertiary general hospital in China.
2. To determine the amount and cost of drug wastage that can be saved by implementing a real-time vial-sharing strategy using intelligent robots that dispense multiple prescriptions simultaneously.

## Methods

### Data sources

This study was conducted retrospectively at Pharmacy Intravenous Admixture Services (PIVAS) of the People's Hospital of Sichuan Province, China. Sichuan Provincial People's Hospital has the largest and most standardized, as well as technologically most advanced and mature, intravenous drug intelligent dispensing center in China, which is directly managed by the Department of Pharmacy and is fully computerized. Introducing intelligent dispensing robots can effectively reduce the exposure of chemotherapy drugs to medical staff (19). The WEINAS intelligent dispensing robots are used to prepare hazardous injectable drugs, such as antineoplastic drugs, automatically. Their operating system enables the dispensation of multiple prescriptions for the same drug simultaneously. First, multiple two-dimensional prescription information codes for the same drug and scanned, and then the drugs are placed into the compounding area at the same time according to the instructions. If any drugs remain in the vial after the dispensation of the previous prescription, they will be used immediately when distributing the next prescription, resulting in real-time sharing. The entire preparation process is continuously verified and recorded for traceability.

More than 90% of chemotherapy drug preparations in the hospital can be done directly by three intelligent robotic systems in a fully enclosed purified space. Drugs that can be shared in vials can all be set up in advance on the robot system following to the specifications of drugs and their characteristics, without the need to specify their dosage and specifications. For each new drug introduced, a robot engineer can perform experimental debugging and then enter the corresponding instructions for the robot to perform the sharing operation. As a precondition for drug sharing, the drug must be capable of being dispensed individually using a robot. Firstly, administration by a robot does not affect the physicochemical properties of the drug, drugs that foam after shaking are not suitable. Also, drugs do not reduce the efficiency of a robot's dispensing, but drugs that must still be left to stand for a period after adding solvents to them are not appropriate. The removed drugs are shown in Table 1. Excluding these special cases, any drugs that can be dispensed using the intelligent dispensing robot can be shared.

**Table 1:** Drugs not considered for vial sharing by intelligent dispensing robots.

**Removed drug**	**Manufacturer**	**Specification (mg)**	**Drug characteristic**	**Reason**
Paclitaxel for injection (Albumin Bound)	Jiangsu hengrui medicine	100	Foaming	It foams and needs to be left to stand for a while after the addition of the corresponding solvent.
Camrelizumab for injection	Jiangsu hengrui medicine	200	Foaming	It foams
Kangai injection	Changbaishan pharmaceutical	10	None	Because the vial specification is too small, it would require a significant amount of vials per prescription and would result in inefficient dispensing by the robot.
Azacitidine for injection	Sichuan huyu pharmaceutical	100	Instability	Ready-to-use
Mesna injection	Jiangsu hengrui medicine	400	None	Administration by intravenous bolus (Not including intravenous infusion)

### Design of the study

This study aimed to determine the potential cost savings of implementing real-time vial-sharing in Chinese hospitals using intelligent dispensing robots at PIVAS. To realize this goal, the investigation utilized the simultaneous preparation of multiple prescriptions feature of the hospital's intelligent dispensing robots to carry out the experimental design. The intelligent dispensing robot uses a real-time vial-sharing strategy that works by placing several prescriptions for the same drug on the operating table at the same time and then using a specially designed syringe with needles on both ends, one of which is inserted into the vial and the other into the infusion bag. Upon entering a command to share a prescription, the needle in the vial is not withdrawn, but the needle in the infusion bag is withdrawn and inserted into another infusion bag, and then the remaining drug in the vial is withdrawn into the infusion bag, thus completing the process of vial-sharing. This whole process of vial-sharing can be set up in the robot system without fear of drug instability associated with the process after opening or increased risk of the rubber falling off caused by extracting the vial of liquid several times. On the contrary, as we uncovered in this paper, the procedure instead reduces the time cost.

The time interval used for real-time sharing preparation was set based on the time between one start-up and the shutdown of the intelligent dispensing robots. The working hours of the robots are the same as the staff working hours every day: the robots work during two periods, from 8:00 a.m. to 12:00 a.m. and from 2:00 to 5:00 p.m. By calculating the amount and cost of wasted chemotherapy drugs, we evaluated the possibility of achieving cost savings in a Chinese hospital PIVAS using a real-time vial-sharing strategy in which intelligent robots dispensed multiple prescriptions simultaneously.

### Date collection and calculation

**Figure 1:**
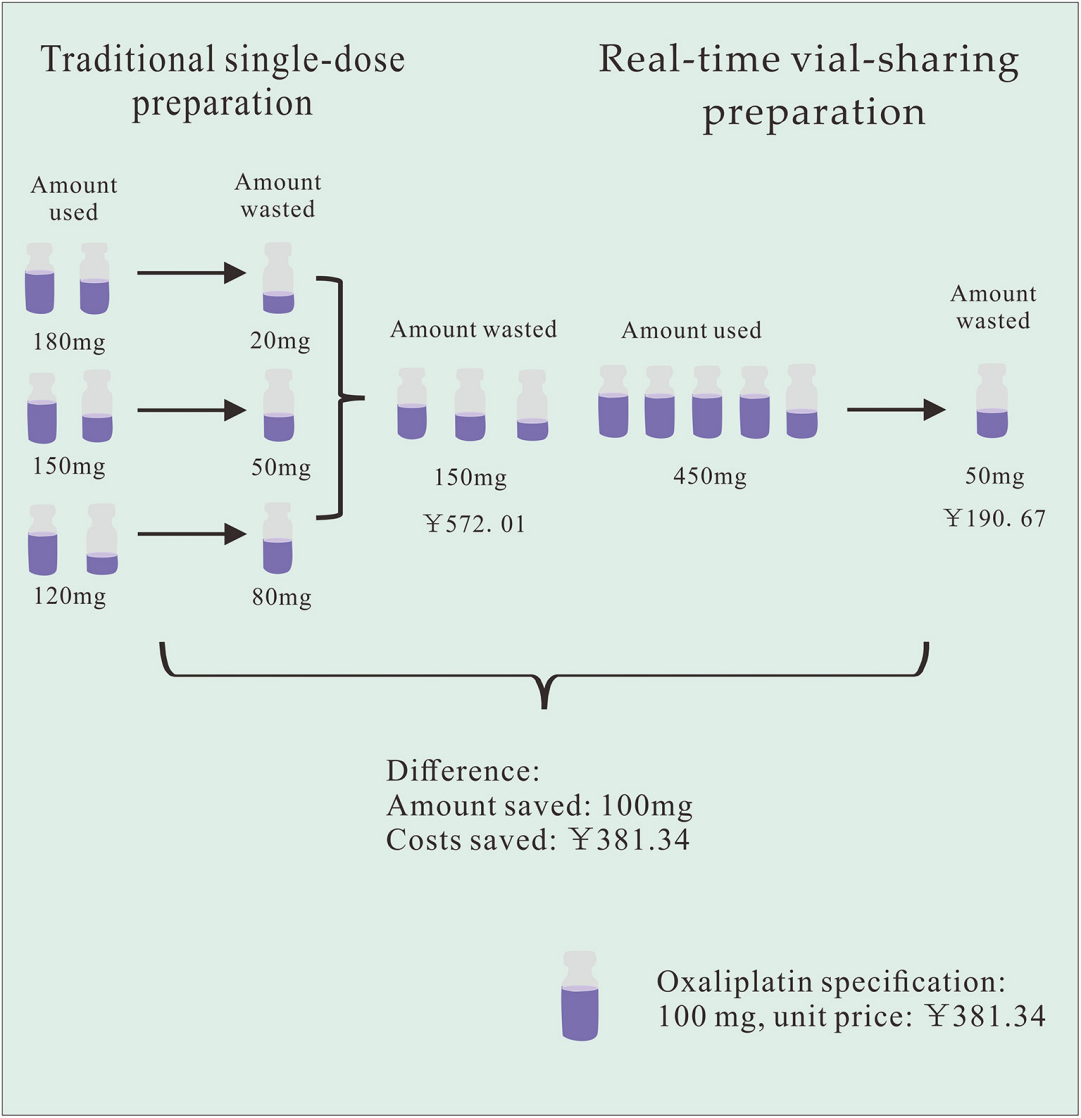
Example of cost-saving diagrams with Oxaliplatin at one time interval.

Using HIS, we retrospectively observed all drug prescriptions that potentially generated waste at PIVAS from September-November 2021. The information collected included the name of the prescribed drug, the actual dose of the drug, drug specifications, number of vials used, and unit price per vial of the drug. The amount of waste and the cost of the wasted drug were calculated based on the difference between patient usage and vial specifications. The determination of drug costs was predicated on the unit price of the drug per milligram. The amount of drug waste, the cost of drug waste, and the number of drug prescriptions that generated waste were also analyzed and compared. Finally, assuming that vial-sharing was implemented in the manner described in Table 2, the total number of vials used for each drug each time was utilized to estimate the number and cost of vials that could be saved for each drug. The savings diagram is shown in Figure 1.

**Table 2:** Example of total *Oxaliplatin waste costs for traditional single-dose preparation and vial-sharing preparation options.

**Preparation method**	**Date**	**Patient**	**Amount used (mg)**	**Number of vials used**	**Amount wasted (mg)**	**#Drug wasted cost (CNY)**
Traditional single-dose preparations	9/1 am	Patient 1	180	2	20	¥76.27
		Patient 2	120	2	80	¥305.07
		Patient 3	150	2	50	¥190.67
	9/1 pm	Patient 4	180	2	20	¥76.27
		Patient 5	140	2	60	¥228.80
		Patient 6	150	2	50	¥190.67
	Total		920	12	280	¥1,067.75
Vial-sharing preparations	9/1 am	Total at am	450	5	50	¥190.67
	9/1 pm	Total at pm	470	5	30	¥114.40
	Total		920	10	80	¥305.07

*Oxaliplatin: the specification is 100 mg, and the unit price of the drug is ¥381.34 per vial. #Drug wasted cost (CNY) = [Unit price (CNY/vial)/Specifications (mg)] * Amount wasted (mg).

### Statistical analysis

Microsoft Office Professional Plus Excel 2019 was used to compile and analyze the data in this study. The amount of waste, drug waste costs, and cost saved of drugs are collated and summed. As continuous variables, they are reported as outcomes in this study. A drug waste cost is the unit price per milligram of a drug multiplied by the amount of the drug wasted. This paper contains a small sample of quantitative information for a paired design. We conducted a hypothetical test on drug waste costs, and the difference does not conform to the normal distribution by the normality test. So, using the paired *t*-test is not appropriate. The Wilcoxon signed-rank test should be employed instead. A *p*-value < 0.05 indicated statistical significance. The percentages of the amount of waste, waste costs, number of waste-generating drug prescriptions, and cost saved for each drug are displayed on the fan charts.

## Results

### Amount of waste

**Figure 2:**
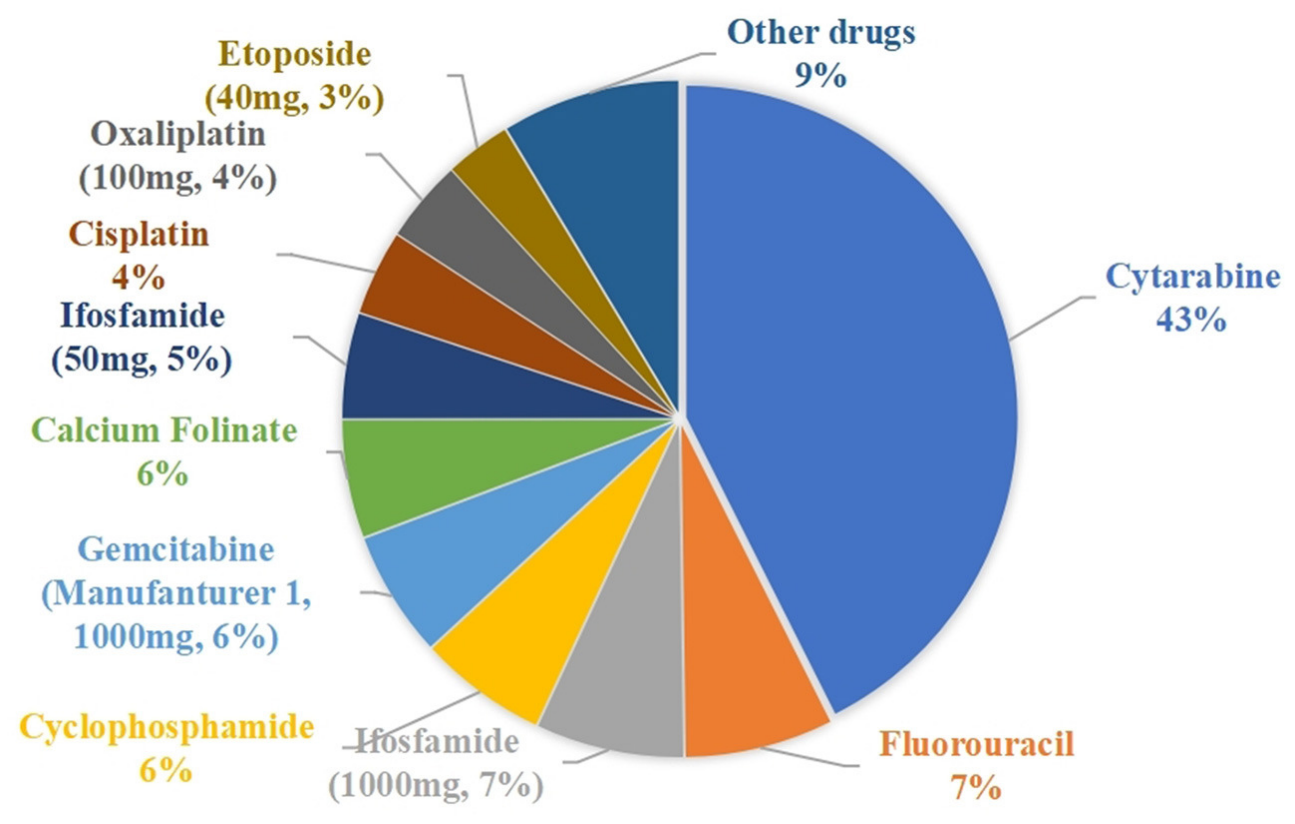
Percentage of drug wastage.

During the 3-month study period, a total of 3,509 cases of waste-generating prescriptions were collected: these included 46 different drugs that were wasted, with an average number of prescriptions per month being 1,170. Table 3 summarizes the number of prescriptions, vial specifications, the unit price per vial, amount wasted, and drug waste cost for the 46 drugs. The traditional single-dose preparation produced a total drug waste of up to 314,898.625 mg. The percentage of the amount of waste per drug is shown in Figure 2: the top five were cytarabine (42.58%), fluorouracil (7.21%), ifosfamide of 100 mg (7.18%), cyclophosphamide (6.22%), and gemcitabine of the 1000 mg from manufacturer 1 (6.10%).

**Table 3:** Summary of information on waste-generating prescribed chemotherapy drugs at PIVAS.

**No**.	**Drug**	**Specifications (mg)**	**Unit price (CNY/vial)**	**Number of prescriptions**	**Number of vials used**		**Amount wasted (mg)**		**Drug wasted cost (CNY)**	
					**Traditional** **single-dose** **preparations**	**Vial-sharing** **preparations**	**Traditional** **single-dose** **preparations**	**Vial-sharing** **preparations**	**Traditional** **single-dose** **preparations**	**Vial-sharing** **preparations**
1	Oxaliplatin	100	¥381.34	253	527	444	12,558.86	4,258.86	¥47,891.96	¥16,240.74
2	Paclitaxel	100	¥780.00	174	708	688	5,869.5	3,869.5	¥45,782.10	¥30,182.10
3	Cytarabine	500	¥132.00	376	418	211	134,075	30,575	¥35,395.80	¥8,071.80
4	Etoposide	40	¥251.95	234	509	431	4,469	1,349	¥28,149.11	¥8,497.01
5	Calcium folinate	100	¥124.20	240	316	169	17,939.3	3,239.3	¥22,280.61	¥4,023.21
6	Pemetrexed disodium	100	¥789.00	58	400	395	2,315	1,815	¥18,265.35	¥14,320.35
7	Gemcitabine/manufacture 1	1000	¥710.00	45	83	82	19,220	18,220	¥13,646.20	¥12,936.20
8	Methotrexate	10	¥174.89	89	172	133	647.125	257.125	¥11,317.57	¥4,496.86
9	Loplatin	50	¥1,766.70	33	35	34	315	265	¥11,130.21	¥9,363.51
10	Irinotecan	40	¥489.34	45	298	295	754	634	¥9,224.06	¥7,756.04
11	Cisplatin	30	¥19.12	741	1,556	1,174	12,929.4	1,469.4	¥8,240.34	¥936.50
12	Vincristine	1	¥195.00	86	147	130	42.04	25.04	¥8,197.80	¥4,882.80
13	Bevacizumab	100	¥1,500.00	8	38	38	420	420	¥6,300.00	¥6,300.00
14	Calcium levofolinate	50	¥124.20	70	112	78	2,401.8	701.8	¥5,966.07	¥1,743.27
15	Ifosfamide	1000	¥204.80	50	127	121	22,600	16,600	¥4,628.48	¥3,399.68
16	Oxaliplatin	50	¥2,100.00	6	24	24	106	106	¥4,452.00	¥4,452.00
17	Fluorouracil	250	¥49.00	189	1,199	1,150	22,697	10,447	¥4,448.61	¥2,047.61
18	Docetaxel/manufacturer 1	20	¥297.16	22	130	128	216	176	¥3,209.33	¥2,615.01
19	Oxaliplatin	50	¥236.80	29	101	95	595	295	¥2,817.92	¥1,397.12
20	Cyclophosphamide	200	¥24.15	194	734	688	19,590	10,390	¥2,365.49	¥1,254.59
21	Epirubicin/manufacturer 1	10	¥86.25	51	306	305	255	245	¥2,199.38	¥2,113.13
22	Trastuzumab	440	¥5,500.00	2	2	2	160	160	¥2,000.00	¥2,000.00
23	Pemetrexed disodium	500	¥2,735.83	2	4	4	350	350	¥1,915.08	¥1,915.08
24	Paclitaxel/manufacturer 1	30	¥228.00	20	137	137	250	250	¥1,900.00	¥1,900.00
25	Loplatin	10	¥438.04	9	39	39	42	42	¥1,839.77	¥1,839.77
26	Gemcitabine/manufacturer 1	200	¥122.61	24	92	92	2,570	2,570	¥1,575.54	¥1,575.54
27	Ifosfamide	500	¥39.10	67	312	306	16,050	13,050	¥1,255.11	¥1,020.51
28	Paclitaxel/manufacturer 2	30	¥137.65	23	164	162	352	292	¥1,615.09	¥1,339.79
29	Docetaxel/manufacturer 2	20	¥1,300.00	2	12	12	20	20	¥1,300.00	¥1,300.00
30	Ratitrexed	2	¥669.00	4	10	10	3.2	3.2	¥1,070.40	¥1,070.40
31	Homotrimoxaline	1	¥96.00	17	39	39	8.5	8.5	¥816.00	¥816.00
33	Etoposide	100	¥7.79	223	239	165	9,976.6	2,576.6	¥777.18	¥200.72
34	Bortezomib	1	¥298.95	7	16	16	2.3	2.3	¥687.59	¥687.59
34	Bleomycin	15	¥119.00	7	14	14	70	70	¥555.33	¥555.33
35	Rubidomycin	20	¥26.88	34	64	64	402.8	402.8	¥541.36	¥541.36
36	Gemcitabine/manufacturer 2	1000	¥205.63	5	10	10	2,250	2,250	¥462.67	¥462.67
37	Actinomycin D	0.2	¥119.00	6	18	18	0.7	0.7	¥416.50	¥416.50
38	Carboplatin	50	¥30.35	28	191	188	619.5	469.5	¥376.04	¥284.99
39	Dextrazoxane	250	¥336.01	5	7	7	230	230	¥309.13	¥309.13
40	Docetaxel/manufacturer 3	20	¥54.12	8	45	45	70	70	¥189.42	¥189.42
41	Nedaplatin	50	¥326.70	2	6	6	20	20	¥130.68	¥130.68
42	Nedaplatin	10	¥55.00	4	55	55	17	17	¥93.50	¥93.50
43	Gemcitabine/manufacturer 2	200	¥59.98	2	7	7	200	200	¥59.98	¥59.98
44	Doxorubicin	10	¥22.92	6	15	15	17	17	¥38.96	¥38.96
45	Mesna	400	¥8.63	6	12	10	1,200	400	¥25.89	¥8.63
46	Epirubicin/manufacturer 2	10	¥122.00	1	14	14	2	2	¥24.40	¥24.40
			Total	3,509	9,464	8,250	314,898.625	128,831.625	¥315,884.00	¥165,810.47

### Drug waste costs

**Figure 3:**
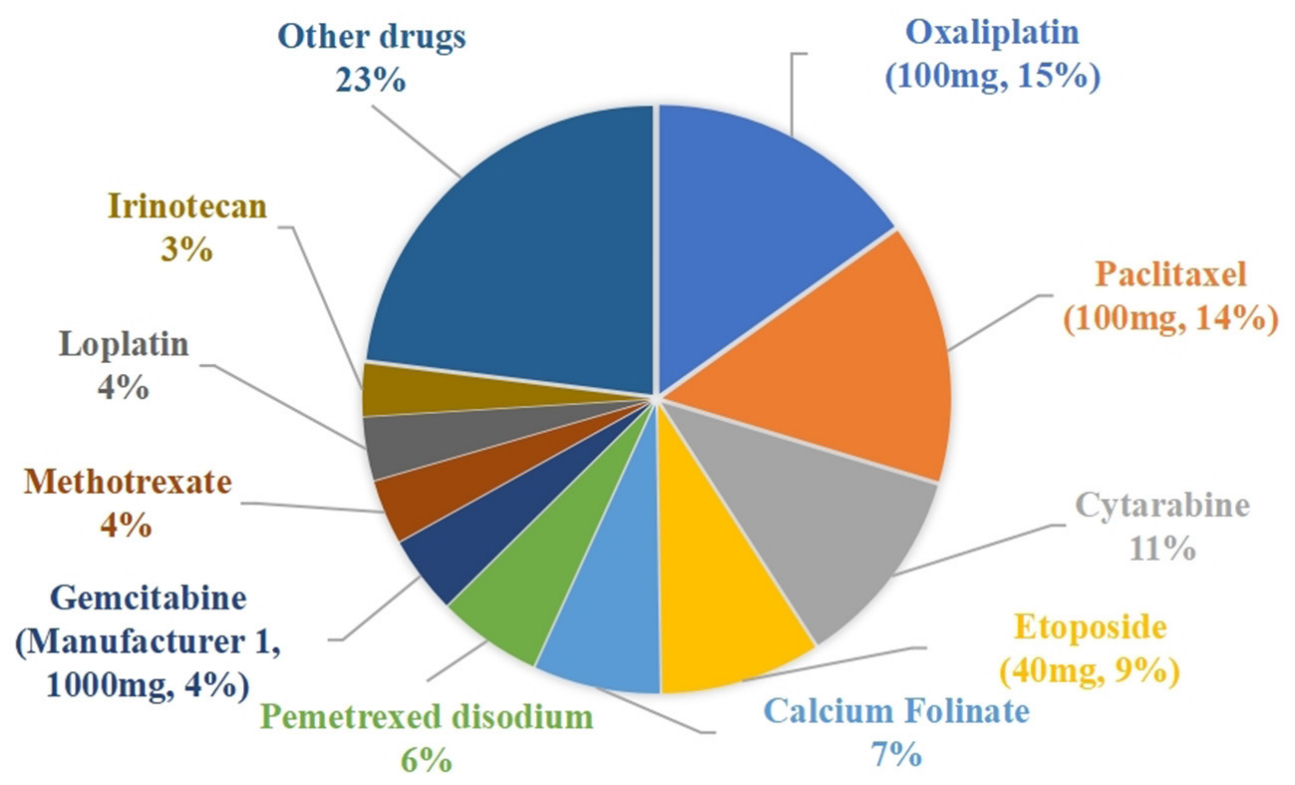
Percentage of drug waste costs.

The drug waste costs for the traditional single-dose preparations and the vial-sharing preparations were 315,884 CNY and 165,810 CNY (*p* = 0.0000194 < 0.05), respectively. The percentage of wasted expenses per drug class ranged from 0.01 to 15.16%, as shown in Figure 3. The top 5 drugs accounting for more than 56% of the total drug waste costs were oxaliplatin (100 mg, 15.16%), paclitaxel (100 mg, 14.48%), cytarabine (11.21%), etoposide (40 mg, 8.91%), and calcium folinate (7.05%).

### Number of drug prescriptions

**Figure 4:**
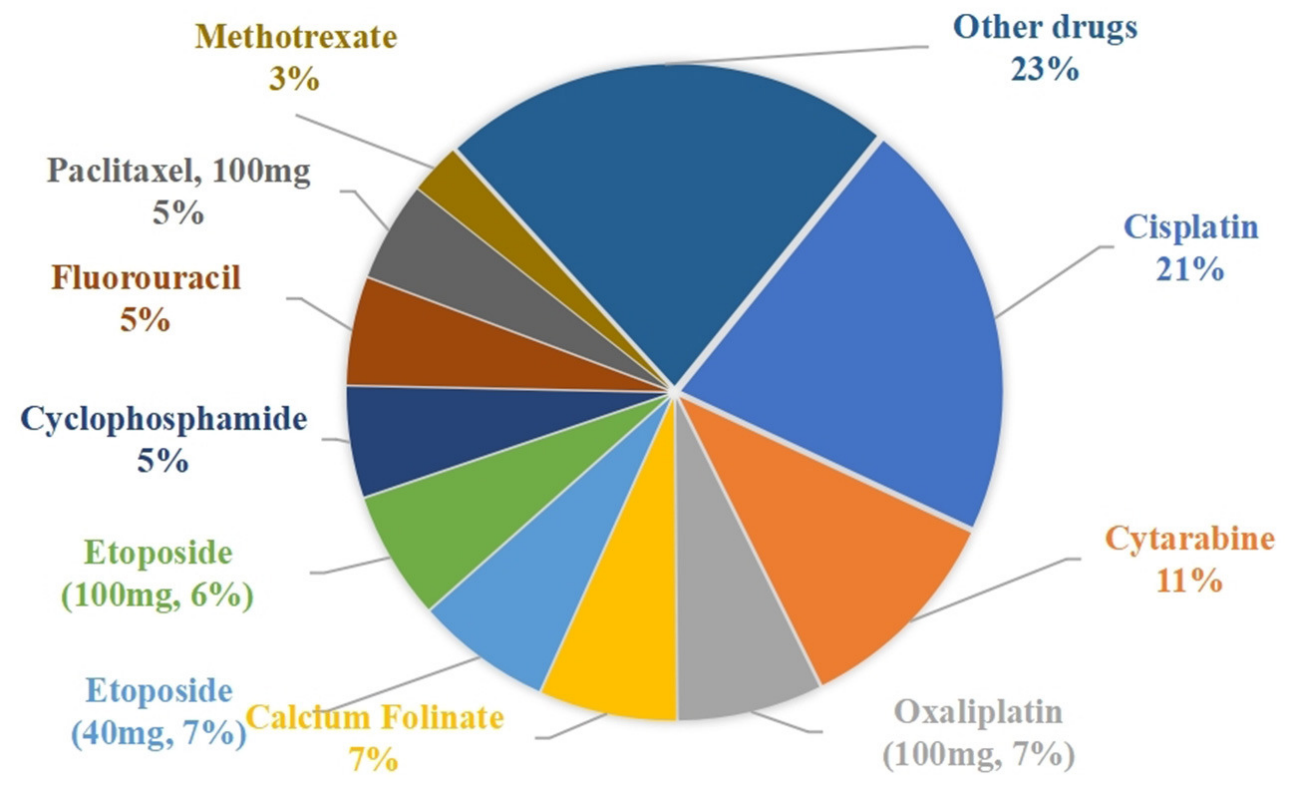
Percentage of waste-generating drug prescriptions.

The percentage of waste-generating drug prescriptions is shown in Figure 4. The top five drugs were cisplatin (21.12%), cytarabine (10.72%), oxaliplatin (100 mg, 7.21%), calcium Folinate (6.84%), and etoposide (40 mg, 6.73%).

### Cost saved

**Figure 5:**
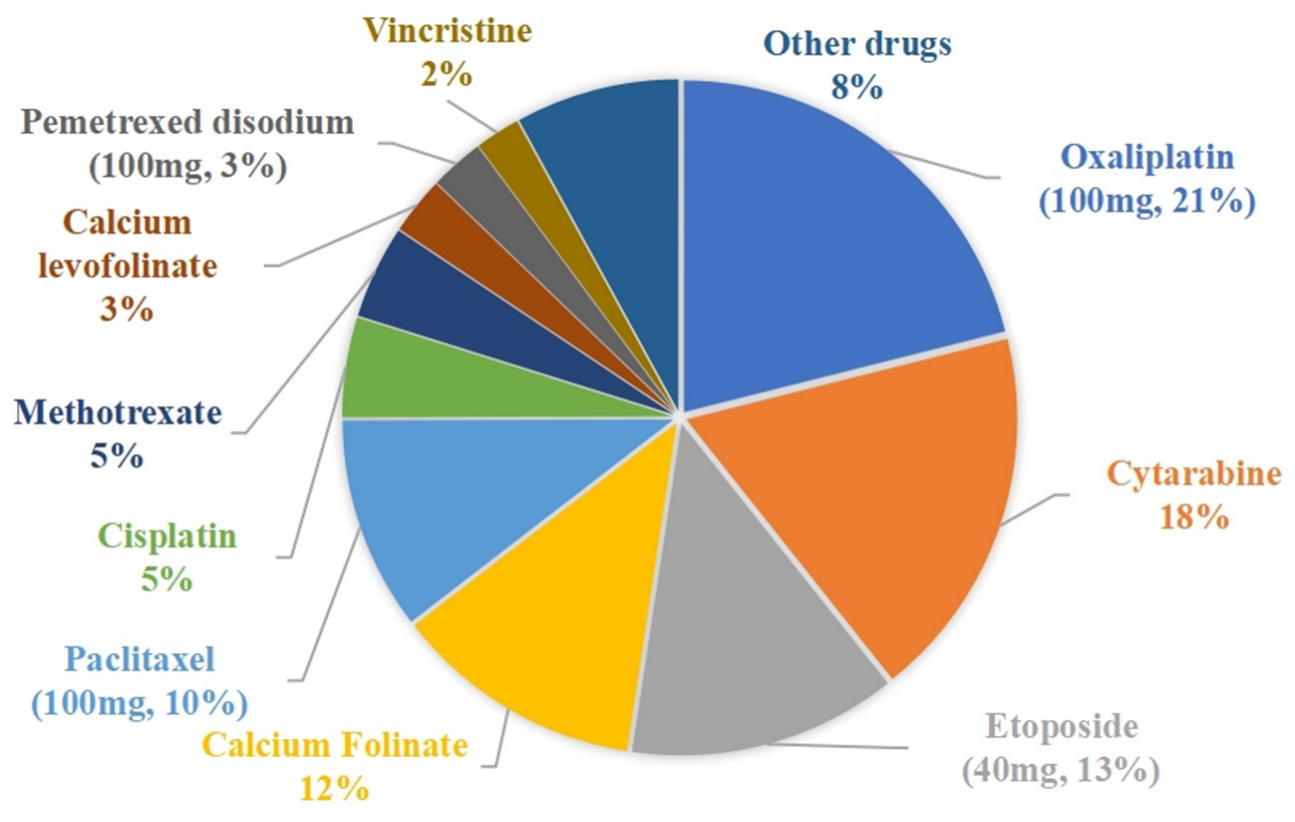
Percentage of drug costs saved.

The cost savings results are shown in Table 4. Vial-sharing achieved cost savings for 24 drugs. Using the estimates from the outcome of those 24 medications, the vial-sharing strategy saved 186,067 mg of drugs, or ~59.08% of the total amount wasted, resulting in savings of 150,073.53 CNY, or 47.51% of the cost of the total waste. The percentage of drug cost savings is shown in Figure 5: oxaliplatin (100 mg, 21.09%), cytarabine (18.21%), etoposide (40 mg, 13.09%), calcium folinate (12.17%), and paclitaxel (100 mg, 10.39%).

**Table 4:** Wasted drugs and costs saved through vial-sharing preparations.

**No**.	**Drug**	**Specifications** **(mg)**	**Unit price** **(CNY/vial)**	**Number of** **vials saved**	**Amount saved** **(mg)**	**Cost saved** **(CNY)**
1	Oxaliplatin	100	¥381.34	83	8,300	¥31,651.22
2	Cytarabine	500	¥132.00	207	103,500	¥27,324.00
3	Etoposide	40	¥251.95	78	3,120	¥19,652.10
4	Calcium folinate	100	¥124.20	147	14,700	¥18,257.40
5	Paclitaxel	100	¥780.00	20	2,000	¥15,600.00
6	Cisplatin	30	¥19.12	382	11,460	¥7,303.84
7	Methotrexate	10	¥174.89	39	390	¥6,820.71
8	Calcium levofolinate	50	¥124.20	34	1,700	¥4,222.80
9	Pemetrexed disodium	100	¥2,735.83	5	2,500	¥3,945.00
10	Vincristine	1	¥195.00	17	17	¥3,315.00
11	Fluorouracil	250	¥49.00	49	12,250	¥2,401.00
12	Loplatin	50	¥1,766.70	1	50	¥1,766.70
13	Irinotecan	40	¥489.34	3	120	¥1,468.02
14	Oxaliplatin	50	¥236.80	6	300	¥1,420.80
15	Ifosfamide	1000	¥204.80	6	6,000	¥1,228.80
16	Cyclophosphamide	200	¥24.15	46	9,200	¥1,110.90
17	Gemcitabine/manufacturer 1	1000	¥710.00	1	1,000	¥710.00
18	Docetaxel/manufacturer 1	20	¥297.16	2	40	¥594.32
19	Etoposide	100	¥7.79	74	7,400	¥576.46
20	Paclitaxel/manufacturer 2	30	¥137.65	2	60	¥275.30
21	Ifosfamide	500	¥39.10	6	3,000	¥234.60
22	Carboplatin	50	¥30.35	3	150	¥91.05
23	Epirubicin/manufacturer 1	10	¥86.25	1	10	¥86.25
24	Mesna	400	¥8.63	2	800	¥17.26
			Total	1214	186,067	¥150,073.53

## Discussion

In this study, chemotherapy drug wastage was substantial and caused a considerable economic burden. During the 3-month inquiry, a total of 314,898.625 mg of the drugs ended up as waste, with a cost analysis of 315,884 CNY. We found the real-time vial-sharing method to have significant cost advantages over the traditional single-dose preparation. Via-sharing reduced drug waste by more than half and saved 159,807.68 CNY or 50.5% of the total drug waste costs.

Only 24 of the 46 wasted drugs were cost-saved through real-time vial-sharing. The discrepancies were mainly due to the low frequency of drug use, which resulted in no prescriptions of the same drugs being generated at the same interval, or the prescribed dose was so large that the remaining drugs could not be saved even after they were shared (e.g., if two patients need 80 mg of oxaliplatin with a specification of 100 mg, the extra servings can only produce waste and would not be shared at that interval). The possibility of saving medications through real-time vial-sharing is closely associated with the frequency of administration and the differences between common doses and vial specifications in the population. For drugs with a large amount of waste that cannot adopt the vial-sharing strategy, medical institutions can optimize drug specifications to select chemotherapy drugs with smaller sizes as much as possible during drug selection. In this study, oxaliplatin, etoposide of 40 mg, calcium folinate, and paclitaxel of 100 mg were highly cost-effective when administered using the real-time vial-sharing strategy. It is recommended that hospitals carry out the vial-sharing strategy for frequently used chemotherapy drugs, expensive drugs, and drugs that generate a huge amount of waste.

Appropriate specifications of the drug are important to reduce drug waste. It is recommended that medical institutions adjust the specifications of hospital drugs, which can also effectively reduce drug waste. Taking cisplatin as an example, we compared the information collected on drug prescriptions. We found that the specification of the drug vial was 30 mg, while the common doses for the population were mostly 40 mg or 50 mg, which is why the number of drug prescriptions that generated waste was so high (741, 21.12%). This is a very clear indication that pharmaceutical companies must redesign or increase the specifications for this drug and that medical institutions should base their drug purchases on doses commonly used in the population. Redesigning the vial specification will make it easier to match the doses commonly used by the population and inevitably reduce the amount of drug waste currently in play.

The biggest percentage of wasted costs was oxaliplatin at 100 mg in the pre-study period, as it was only available in a single larger specification. In the course of the study, the country conducted a new round of centralized purchasing of 50 mg of oxaliplatin. Therefore, during the latter part of the trial data collection (from November 15), the hospital started supplying 50 mg of oxaliplatin. In our comparison of the number of prescriptions and drug waste, we found that a total of 253 prescriptions of 100 mg of oxaliplatin produced wastes of up to 12,553.86 mg, an average waste of 49.62 mg per prescription. While 29 prescriptions of 50 mg of oxaliplatin only generated 595 mg of waste, an average waste of 20.5 mg per prescription, indicating that the proportion of wasted oxaliplatin is significantly lower for smaller specifications than for larger ones. This finding also suggests that drug procurement by medical institutions based on population dose requirements can be effective in limiting drug waste and that the amount of drug wastage is much lower with the provision of smaller specifications than with larger ones.

Due to differences in the health insurance system, few studies on the economics of vial-sharing have been conducted in China. Regrettably, medical institutions do not pay attention to the chemotherapy drugs left in vials, and patients are charged for the total amount of drug per vial rather than the actual dose received. However, investigations in the UK (20) and Japan (21) have shown that using leftover vials results in significant cost-saving, especially for molecularly targeted drugs. In the UK and Japanese inquiries, vials were reused for 7 and 1 day, respectively. However, our estimates were based on the number of vials needed to achieve half-day sharing. Therefore, the proportion of potential economic savings in China may exceed the results we have obtained in this study. Under China's drug cost methodology, implementing vial-sharing means that the cost of using a CSTD or intelligent dispensing robots will be borne by medical institutions. While medical institutions using intelligent dispensing robots for drug preparation will still charge patients for preparations, the cost will be much less than the expenses on wasted drug; so if this aspect is applied to the actual process, then patients, medical institutions, and national health insurance agencies will all benefit. Additionally, it will reduce the risk of exposing medications to healthcare workers during the process of chemotherapy drug dispensing (19) as well as the cost of disposing of waste fluids in healthcare facilities (17). To implement the vial-sharing strategy more effectively, we need to get the support of professional pharmacy organizations and government bodies. Government departments should provide guidelines for the application of partially used vials and guidelines on compounding to offer recommendations for assigning the beyond-use dating (BUD) on compounded sterile injectable products. The Society of Hospital Pharmacists should support the practice of vial-sharing in specialized pharmacy aseptic manufacturing sites and licensed compounding facilities following rigorous governance frameworks and professional standards of practice. The National health system should provide opportunities for achieving financial savings. It should have appropriate reimbursement plans for these drugs. In addition, medical institutions can also use The Closed system transfer devices (CSTDs) to optimize vial sharing, which can prevent contamination of drug products and has the potential to allow the extended BUD of single-use vials.

This study is the first to propose the concept of real-time vial-sharing. Notably, the aseptic condition and stability data of the drug composite product are key factors influencing the use of the vial-sharing strategy. Using CSTD will introduce some new risks, and traditional vial-sharing will also require manual intervention for their storage, which could result in the incorrect writing of information or dose miscalculation or omission altogether (21). Rather than use CSTDs to store drugs for reuse in the same period (22), we prepared them simultaneously by employing an intelligent dispensing robot. Therefore, the introduction of an intelligent robot capable of dispensing multiple prescriptions simultaneously to run a real-time vial-sharing strategy in this investigation encountered not the traditional approach-related safety issue concerns, such as the introduction of impurities due to repeated interspersing or recording drug information incorrectly. Using our method will also provide other benefits, including simplicity of the process, reduced risk of errors, and more accurate dosing, rendering its application highly safe and feasible.

While the outcome of this study was impressive, the investigation had its limitations. The cost analysis was performed using data collected from a single facility and only for 3 months, albeit a tertiary general hospital in China. More hospitals should be included in future projects for a more generalizable outcome. Due to the limitations of the health insurance system and national policies, the multi-prescription real-time vial-sharing strategy using intelligent dispensing robots was not practically applied. This paper only presented a theoretical basis for conducting this strategy, and, therefore, in subsequent research, we will aim to examine the practical feasibility of this strategy. In addition, the cost of introducing an intelligent dispensing robot was not discussed in this study. The purchase and maintenance of robots and the use of special blending devices that assist in sharing are expensive, whereas manual operations do not have this expense. What is more, there is currently no way to avoid the wastage of chemotherapy drugs caused by the use of intelligent dispensing robots; however, we can minimize this wastage, for example, by scheduling the same prescribed drugs for the same period whenever possible. Lastly, drugs that are shared using intelligent dispensing robots are not currently charged exactly for the actual dose used, with the resulting cost of wasted drugs borne by patients. In practice, health institutions typically charge patients for the actual number of prescription vials first. During the dispensation process, if vials are shared between prescriptions, there is a corresponding saving in drugs and costs (the intelligent dispensing robot will scan each dispensed prescription, and the system can monitor and record it in real-time). This saving can subsequently be refunded to the patient's account according to the actual status of prescriptions. To ensure that this strategy works, the health insurance department must also be looped in for agreement. This strategy will also save corresponding costs for the medical insurance department. Theoretically, it will be a win-win-win situation for the patient, the health institution, and the medical insurance department.

## Conclusion

This inquiry, as far as documented evidence is concerned, is the first study from China on reducing waste and cost savings in chemotherapy. It is also the first time that the concept of a real-time vial-sharing strategy has been proposed. According to our estimates, an oncology drug waste reduction to control costs is feasible and economically beneficial. Notably, medical institutions with PIVAS can achieve waste reduction and cost savings by introducing intelligent dispensing robots to share drug vials in real-time for multi-prescription dispensing of chemotherapy drugs. This not only saves medical resources and reduces exposure risks but also eases the huge burden on patients, medical institutions, and the national medical insurance system. This certainly is a multi-win situation. Additionally, with real-time sharing, the aseptic condition and stability data of the drug composite product can be easily assured. Based on our findings, we also recommend that medical institutions prioritize scrutinizing drugs in terms of their unit price, frequency of use, prescription dose, and common population dose to determine which medications are appropriate for a real-time vial-sharing strategy.

## Data availability statement

The original contributions presented in the study are included in the article/Supplementary material, further inquiries can be directed to the corresponding author/s.

## Author contributions

HL and JY conceived and designed the study. HL, JY, LZ, and YS collected and analyzed the data. HL wrote the first draft of the manuscript. HL, JY, and LZ critically revised it. All authors checked the data and made contributions to this study.

## Funding

This work was funded by the National Key Research and Development Program of China (2020YFC2005506), the Department of Science and Technology of Sichuan Province of China (2019YFS0514), and the Personalized Drug Therapy Key Laboratory of Sichuan Province (2021YB10).

## Conflict of interest

The authors declare that the research was conducted in the absence of any commercial or financial relationships that could be construed as a potential conflict of interest.

## Publisher's note

All claims expressed in this article are solely those of the authors and do not necessarily represent those of their affiliated organizations, or those of the publisher, the editors and the reviewers. Any product that may be evaluated in this article, or claim that may be made by its manufacturer, is not guaranteed or endorsed by the publisher.
