# Supplementary material for: Cost analysis of implementing a vial-sharing strategy for chemotherapy drugs using intelligent dispensing robots in a tertiary Chinese hospital in Sichuan
Source: Front Public Health. 2022 Sep 21;10:936686. doi: 10.3389/fpubh.2022.936686 (PMC9534289; doi:10.3389/fpubh.2022.936686)
Supplement: Supplementary file 1 [file Table_1.pdf]

Supplementary Data: Detailed waste-generating prescription informations

Date	Drug	Specifications (mg)	Amount used	Number of vials used	Amount wasted（mg）
9/9 am	Oxaliplatin	100	180	2	20
9/9 am	Oxaliplatin	100	180	2	20
9/9 am	Oxaliplatin	100	150	2	50
9/8 am	Oxaliplatin	100	180	2	20
9/8 am	Oxaliplatin	100	140	2	60
9/8 am	Oxaliplatin	100	150	2	50
9/7 pm	Oxaliplatin	100	240	3	60
9/7 am	Oxaliplatin	100	240	3	60
9/4 pm	Oxaliplatin	100	150	2	50
9/4 am	Oxaliplatin	100	90	1	10
9/3 pm	Oxaliplatin	100	150	2	50
9/3 pm	Oxaliplatin	100	120	2	80
9/3 am	Oxaliplatin	100	140	2	60
9/3 am	Oxaliplatin	100	65	1	35
9/3 am	Oxaliplatin	100	65	1	35
9/3 am	Oxaliplatin	100	115	2	85
9/3 am	Oxaliplatin	100	180	2	20
9/3 am	Oxaliplatin	50	125	3	25
9/30 pm	Oxaliplatin	100	150	2	50
9/30 am	Oxaliplatin	100	150	2	50
9/30 am	Oxaliplatin	100	150	2	50
9/30 am	Oxaliplatin	100	140	2	60
9/2 pm	Oxaliplatin	100	150	2	50
9/2 am	Oxaliplatin	100	230	3	70
9/2 am	Oxaliplatin	100	150	2	50
9/2 am	Oxaliplatin	100	250	3	50
9/2 am	Oxaliplatin	100	120	2	80
9/2 am	Oxaliplatin	100	228.8	3	71.2
9/2 am	Oxaliplatin	100	220	3	80
9/29 am	Oxaliplatin	100	180	2	20
9/29 am	Oxaliplatin	100	90	1	10
9/28 am	Oxaliplatin	100	150	2	50
9/28 am	Oxaliplatin	100	150	2	50
9/27 am	Oxaliplatin	100	230	3	70
9/27 am	Oxaliplatin	100	220	3	80
9/27 am	Oxaliplatin	100	150	2	50
9/27 am	Oxaliplatin	100	120	2	80
9/27 am	Oxaliplatin	100	150	2	50
9/26 pm	Oxaliplatin	100	150	2	50
9/26 pm	Oxaliplatin	100	150	2	50
9/25 am	Oxaliplatin	100	150	2	50
9/25 am	Oxaliplatin	100	80	1	20
9/24 am	Oxaliplatin	100	240	3	60
9/24 am	Oxaliplatin	100	180	2	20
9/24 am	Oxaliplatin	100	150	2	50
9/23 am	Oxaliplatin	100	140	2	60

9/23 am	Oxaliplatir	100	110	2	90
9/23 am	Oxaliplatir	100	240	3	60
9/21 am	Oxaliplatir	100	240	3	60
9/20 am	Oxaliplatir	100	150	2	50
9/1 am	Oxaliplatir	100	135	2	65
9/1 am	Oxaliplatir	100	180	2	20
9/1 am	Oxaliplatir	100	125	2	75
9/19 pm	Oxaliplatir	100	210	3	90
9/19 am	Oxaliplatir	100	250	3	50
9/19 am	Oxaliplatir	100	65	1	35
9/19 am	Oxaliplatir	100	65	1	35
9/18 am	Oxaliplatir	100	70	1	30
9/18 am	Oxaliplatir	100	70	1	30
9/18 am	Oxaliplatir	100	236	3	64
9/18 am	Oxaliplatir	100	210	3	90
9/17 am	Oxaliplatir	100	150	2	50
9/17 am	Oxaliplatir	100	220	3	80
9/17 am	Oxaliplatir	100	150	2	50
9/17 am	Oxaliplatir	100	240	3	60
9/17 am	Oxaliplatir	100	65	1	35
9/17 am	Oxaliplatir	100	65	1	35
9/16 am	Oxaliplatir	100	150	2	50
9/16 am	Oxaliplatir	100	150	2	50
9/16 am	Oxaliplatir	100	190	2	10
9/16 am	Oxaliplatir	100	190	2	10
9/16 am	Oxaliplatir	100	220	3	80
9/16 am	Oxaliplatir	100	170	2	30
9/15 am	Oxaliplatir	100	150	2	50
9/15 am	Oxaliplatir	100	180	2	20
9/15 am	Oxaliplatir	100	240	3	60
9/15 am	Oxaliplatir	100	190	2	10
9/14 am	Oxaliplatir	100	120	2	80
9/14 am	Oxaliplatir	100	150	2	50
9/14 am	Oxaliplatir	100	150	2	50
9/14 am	Oxaliplatir	100	160	2	40
9/14 am	Oxaliplatir	100	250	3	50
9/14 am	Oxaliplatir	100	250	3	50
9/13 pm	Oxaliplatir	100	150	2	50
9/13 am	Oxaliplatir	100	65	1	35
9/13 am	Oxaliplatir	100	65	1	35
9/13 am	Oxaliplatir	100	240	3	60
9/13 am	Oxaliplatir	100	140	2	60
9/13 am	Oxaliplatir	100	50	1	50
9/11 pm	Oxaliplatir	100	150	2	50
9/11 am	Oxaliplatir	100	180	2	20
9/11 am	Oxaliplatir	100	180	2	20
9/11 am	Oxaliplatir	100	170	2	30
9/11 am	Oxaliplatir	100	150	2	50
9/10 am	Oxaliplatir	100	220	3	80
9/10 am	Oxaliplatir	100	180	2	20
11/9 am	Oxaliplatir	100	140	2	60

11/9 am	Oxaliplatir	100	240	3	60
11/9 am	Oxaliplatir	100	120	2	80
11/9 am	Oxaliplatir	100	150	2	50
11/9 am	Oxaliplatir	100	160	2	40
11/8 am	Oxaliplatir	100	120	2	80
11/7 am	Oxaliplatir	100	170	2	30
11/7 am	Oxaliplatir	100	180	2	20
11/6 am	Oxaliplatir	100	150	2	50
11/6 am	Oxaliplatir	100	150	2	50
11/6 am	Oxaliplatir	100	150	2	50
11/6 am	Oxaliplatir	100	150	2	50
11/6 am	Oxaliplatir	100	150	2	50
11/6 am	Oxaliplatir	100	150	2	50
11/6 am	Oxaliplatir	100	180	2	20
11/6 am	Oxaliplatir	100	240	3	60
11/6 am	Oxaliplatir	100	50	1	50
11/5 pm	Oxaliplatir	100	165	2	35
11/5 am	Oxaliplatir	100	140	2	60
11/5 am	Oxaliplatir	100	125	2	75
11/5 am	Oxaliplatir	100	230	3	70
11/4 pm	Oxaliplatir	100	150	2	50
11/4 am	Oxaliplatir	100	120	2	80
11/3 am	Oxaliplatir	100	150	2	50
11/2 am	Oxaliplatir	100	240	3	60
11/2 am	Oxaliplatir	100	120	2	80
11/25 pm	Oxaliplatir	100	150	2	50
11/25 am	Oxaliplatir	100	150	2	50
11/24 pm	Oxaliplatir	100	150	2	50
11/23 am	Oxaliplatir	100	150	2	50
11/23 am	Oxaliplatir	100	180	2	20
11/22 am	Oxaliplatir	100	150	2	50
11/20 am	Oxaliplatir	100	140	2	60
11/20 am	Oxaliplatir	100	240	3	60
11/1 am	Oxaliplatir	100	120	2	80
11/1 am	Oxaliplatir	100	210	3	90
11/19 pm	Oxaliplatir	100	381.34	4	18.66
11/19 am	Oxaliplatir	100	130	2	70
11/19 am	Oxaliplatir	100	240	3	60
11/18 pm	Oxaliplatir	100	150	2	50
11/18 am	Oxaliplatir	100	150	2	50
11/17 pm	Oxaliplatir	100	150	2	50
11/15 pm	Oxaliplatir	100	150	2	50
11/15 am	Oxaliplatir	100	150	2	50
11/15 am	Oxaliplatir	100	238	3	62
11/15 am	Oxaliplatir	100	150	2	50
11/14 am	Oxaliplatir	100	180	2	20
11/13 am	Oxaliplatir	100	220	3	80
11/13 am	Oxaliplatir	100	240	3	60
11/12 pm	Oxaliplatir	100	150	2	50
11/12 am	Oxaliplatir	100	140	2	60
11/12 am	Oxaliplatir	100	65	1	35

11/12 am	Oxaliplatin	100	65	1	35
11/12 am	Oxaliplatin	100	210	3	90
11/11 am	Oxaliplatin	100	240	3	60
11/10 am	Oxaliplatin	100	136	2	64
11/10 am	Oxaliplatin	100	190	2	10
11/10 am	Oxaliplatin	100	135	2	65
10/9 am	Oxaliplatin	100	150	2	50
10/9 am	Oxaliplatin	100	150	2	50
10/9 am	Oxaliplatin	100	160	2	40
10/9 am	Oxaliplatin	100	150	2	50
10/9 am	Oxaliplatin	100	150	2	50
10/8 pm	Oxaliplatin	100	150	2	50
10/8 pm	Oxaliplatin	100	150	2	50
10/8 am	Oxaliplatin	100	120	2	80
10/8 am	Oxaliplatin	100	240	3	60
10/8 am	Oxaliplatin	100	50	1	50
10/7 am	Oxaliplatin	100	150	2	50
10/7 am	Oxaliplatin	100	150	2	50
10/7 am	Oxaliplatin	100	150	2	50
10/5 am	Oxaliplatin	100	250	3	50
10/3 am	Oxaliplatin	100	180	2	20
10/3 am	Oxaliplatin	100	65	1	35
10/3 am	Oxaliplatin	100	65	1	35
10/3 am	Oxaliplatin	100	195	2	5
10/30 am	Oxaliplatin	100	180	2	20
10/30 am	Oxaliplatin	100	120	2	80
10/2 am	Oxaliplatin	100	180	2	20
10/29 am	Oxaliplatin	100	130	2	70
10/29 am	Oxaliplatin	100	150	2	50
10/28 am	Oxaliplatin	100	150	2	50
10/28 am	Oxaliplatin	100	150	2	50
10/28 am	Oxaliplatin	100	180	2	20
10/28 am	Oxaliplatin	100	220	3	80
10/28 am	Oxaliplatin	100	40	1	60
10/27 pm	Oxaliplatin	100	150	2	50
10/27 am	Oxaliplatin	100	150	2	50
10/27 am	Oxaliplatin	100	70	1	30
10/27 am	Oxaliplatin	100	70	1	30
10/26 pm	Oxaliplatin	100	150	2	50
10/26 pm	Oxaliplatin	100	150	2	50
10/26 am	Oxaliplatin	100	230	3	70
10/26 am	Oxaliplatin	100	230	3	70
10/26 am	Oxaliplatin	100	65	1	35
10/26 am	Oxaliplatin	100	250	3	50
10/26 am	Oxaliplatin	100	65	1	35
10/24 am	Oxaliplatin	100	150	2	50
10/23 am	Oxaliplatin	100	240	3	60
10/23 am	Oxaliplatin	100	180	2	20
10/23 am	Oxaliplatin	100	180	2	20
10/22 pm	Oxaliplatin	100	150	2	50
10/22 pm	Oxaliplatin	100	150	2	50

10/22 am	Oxaliplatir	100	65	1	35
10/22 am	Oxaliplatir	100	65	1	35
10/22 am	Oxaliplatir	100	120	2	80
10/22 am	Oxaliplatir	100	90	1	10
10/22 am	Oxaliplatir	100	240	3	60
10/21 pm	Oxaliplatir	100	220	3	80
10/21 pm	Oxaliplatir	100	150	2	50
10/21 am	Oxaliplatir	100	150	2	50
10/21 am	Oxaliplatir	100	50	1	50
10/21 am	Oxaliplatir	100	180	2	20
10/21 am	Oxaliplatir	100	150	2	50
10/21 am	Oxaliplatir	100	180	2	20
10/20 pm	Oxaliplatir	100	150	2	50
10/20 am	Oxaliplatir	100	140	2	60
10/20 am	Oxaliplatir	100	80	1	20
10/20 am	Oxaliplatir	100	120	2	80
10/1 am	Oxaliplatir	100	150	2	50
10/19 pm	Oxaliplatir	100	150	2	50
10/19 pm	Oxaliplatir	100	150	2	50
10/19 am	Oxaliplatir	100	240	3	60
10/19 am	Oxaliplatir	100	180	2	20
10/19 am	Oxaliplatir	100	140	2	60
10/19 am	Oxaliplatir	100	150	2	50
10/19 am	Oxaliplatir	100	120	2	80
10/19 am	Oxaliplatir	100	160	2	40
10/19 am	Oxaliplatir	100	238	3	62
10/19 am	Oxaliplatir	100	238	3	62
10/18 am	Oxaliplatir	100	190	2	10
10/18 am	Oxaliplatir	100	67.5	1	32.5
10/18 am	Oxaliplatir	100	67.5	1	32.5
10/16 am	Oxaliplatir	100	180	2	20
10/16 am	Oxaliplatir	100	110	2	90
10/16 am	Oxaliplatir	100	150	2	50
10/16 am	Oxaliplatir	100	180	2	20
10/14 pm	Oxaliplatir	100	150	2	50
10/14 am	Oxaliplatir	100	210	3	90
10/14 am	Oxaliplatir	100	170	2	30
10/14 am	Oxaliplatir	100	240	3	60
10/14 am	Oxaliplatir	100	240	3	60
10/14 am	Oxaliplatir	100	140	2	60
10/13 pm	Oxaliplatir	100	150	2	50
10/13 am	Oxaliplatir	100	240	3	60
10/13 am	Oxaliplatir	100	120	2	80
10/12 am	Oxaliplatir	100	150	2	50
10/12 am	Oxaliplatir	100	220	3	80
10/12 am	Oxaliplatir	100	70	1	30
10/12 am	Oxaliplatir	100	70	1	30
10/11 pm	Oxaliplatir	100	150	2	50
10/11 am	Oxaliplatir	100	130	2	70
10/10 am	Oxaliplatir	100	150	2	50
10/10 am	Oxaliplatir	100	150	2	50

10/10 am	Oxaliplatir	100	160	2	40
10/10 am	Oxaliplatir	100	150	2	50
10/10 am	Oxaliplatir	100	150	2	50
Total				527	12558.86
9/28 am	Bevacizum	100	450	5	50
9/18 am	Bevacizum	100	450	5	50
9/17 am	Bevacizum	100	850	9	50
9/13 am	Bevacizum	100	250	3	50
11/4 am	Bevacizum	100	450	5	50
11/1 am	Bevacizum	100	230	3	70
11/10 am	Bevacizum	100	450	5	50
10/26 am	Bevacizum	100	250	3	50
Total				38	420
9/9 am	Docetaxel	20	110	6	10
9/4 am	Docetaxel	20	50	3	10
9/3 am	Docetaxel	20	85	5	15
9/3 am	Docetaxel	20	110	6	10
9/29 am	Docetaxel	20	110	6	10
9/27 am	Docetaxel	20	129	7	11
9/27 am	Docetaxel	20	75	4	5
9/23 am	Docetaxel	20	85	5	15
9/23 am	Docetaxel	20	110	6	10
9/1 am	Docetaxel	20	150	8	10
9/15 am	Docetaxel	20	135	7	5
9/15 am	Docetaxel	20	90	5	10
9/10 am	Docetaxel	20	110	6	10
11/1 pm	Docetaxel	20	150	8	10
10/4 pm	Docetaxel	20	110	6	10
10/27 am	Docetaxel	20	110	6	10
10/26 am	Docetaxel	20	130	7	10
10/26 am	Docetaxel	20	110	6	10
10/20 am	Docetaxel	20	75	4	5
10/19 am	Docetaxel	20	110	6	10
10/15 am	Docetaxel	20	130	7	10
10/11 am	Docetaxel	20	110	6	10
				130	216
11/6 am	Docetaxel	20	135	7	5
11/23 am	Docetaxel	20	110	6	10
11/23 am	Docetaxel	20	90	5	10
11/18 am	Docetaxel	20	130	7	10
11/16 am	Docetaxel	20	75	4	5
11/13 am	Docetaxel	20	110	6	10
11/12 am	Docetaxel	20	85	5	15
11/10 am	Docetaxel	20	95	5	5
				45	70
9/23 pm	Docetaxel	20	110	6	10
10/28 am	Docetaxel	20	110	6	10
				12	20
9/9 am	Fluorourac	0.25	1.9	8	100
9/8 am	Fluorourac	0.25	1.9	8	100
9/8 am	Fluorourac	0.25	0.6	3	150

9/8 am	Fluorourac	0.25	0.6	3	150
9/5 pm	Fluorourac	0.25	1.9	8	100
9/4 pm	Fluorourac	0.25	1.9	8	100
9/4 am	Fluorourac	0.25	1.95	8	50
9/4 am	Fluorourac	0.25	0.875	4	125
9/4 am	Fluorourac	0.25	0.875	4	125
9/4 am	Fluorourac	0.25	0.6	3	150
9/3 pm	Fluorourac	0.25	0.937	4	63
9/3 pm	Fluorourac	0.25	0.937	4	63
9/3 am	Fluorourac	0.25	0.7	3	50
9/3 am	Fluorourac	0.25	1.95	8	50
9/3 am	Fluorourac	0.25	0.625	3	125
9/3 am	Fluorourac	0.25	0.65	3	100
9/3 am	Fluorourac	0.25	0.6	3	150
9/3 am	Fluorourac	0.25	0.6	3	150
9/3 am	Fluorourac	0.25	3.6	15	150
9/30 am	Fluorourac	0.25	1.36	6	140
9/2 am	Fluorourac	0.25	2.6	11	150
9/29 pm	Fluorourac	0.25	1.9	8	100
9/29 am	Fluorourac	0.25	1.36	6	140
9/29 am	Fluorourac	0.25	0.6	3	150
9/29 am	Fluorourac	0.25	3.74	15	10
9/28 am	Fluorourac	0.25	1.9	8	100
9/28 am	Fluorourac	0.25	1.36	6	140
9/26 am	Fluorourac	0.25	1.9	8	100
9/25 am	Fluorourac	0.25	0.64	3	110
9/25 am	Fluorourac	0.25	1.9	8	100
9/25 am	Fluorourac	0.25	3.8	16	200
9/24 am	Fluorourac	0.25	1.9	8	100
9/23 am	Fluorourac	0.25	1.9	8	100
9/23 am	Fluorourac	0.25	3.48	14	20
9/23 am	Fluorourac	0.25	0.7	3	50
9/20 am	Fluorourac	0.25	0.875	4	125
9/20 am	Fluorourac	0.25	0.875	4	125
9/20 am	Fluorourac	0.25	0.6	3	150
9/19 am	Fluorourac	0.25	0.625	3	125
9/19 am	Fluorourac	0.25	0.875	4	125
9/19 am	Fluorourac	0.25	0.875	4	125
9/19 am	Fluorourac	0.25	0.937	4	63
9/19 am	Fluorourac	0.25	0.937	4	63
9/18 pm	Fluorourac	0.25	0.6	3	150
9/18 am	Fluorourac	0.25	0.625	3	125
9/18 am	Fluorourac	0.25	0.875	4	125
9/18 am	Fluorourac	0.25	0.875	4	125
9/18 am	Fluorourac	0.25	1.58	7	170
9/17 pm	Fluorourac	0.25	2.06	9	190
9/17 am	Fluorourac	0.25	0.6	3	150
9/17 am	Fluorourac	0.25	0.625	3	125
9/17 am	Fluorourac	0.25	0.875	4	125
9/17 am	Fluorourac	0.25	0.68	3	70
9/16 am	Fluorourac	0.25	1.8	8	200

9/16 am	Fluorourac	0.25	3.3	14	200
9/15 am	Fluorourac	0.25	3.3	14	200
9/15 am	Fluorourac	0.25	0.6	3	150
9/15 am	Fluorourac	0.25	0.4	2	100
9/14 am	Fluorourac	0.25	3.3	14	200
9/13 am	Fluorourac	0.25	0.625	3	125
9/13 am	Fluorourac	0.25	0.6	3	150
9/11 am	Fluorourac	0.25	0.66	3	90
9/11 am	Fluorourac	0.25	0.6	3	150
11/9 am	Fluorourac	0.25	3.3	14	200
11/9 am	Fluorourac	0.25	0.64	3	110
11/9 am	Fluorourac	0.25	1.9	8	100
11/8 am	Fluorourac	0.25	3.3	14	200
11/7 am	Fluorourac	0.25	3.3	14	200
11/6 am	Fluorourac	0.25	3.4	14	100
11/5 am	Fluorourac	0.25	0.625	3	125
11/4 am	Fluorourac	0.25	4.35	18	150
11/4 am	Fluorourac	0.25	1.7	8	300
11/4 am	Fluorourac	0.25	0.56	3	190
11/3 am	Fluorourac	0.25	1.8	8	200
11/30 pm	Fluorourac	0.25	0.6	3	150
11/2 am	Fluorourac	0.25	1.95	8	50
11/2 am	Fluorourac	0.25	1.8	8	200
11/2 am	Fluorourac	0.25	0.6	3	150
11/29 am	Fluorourac	0.25	0.72	3	30
11/29 am	Fluorourac	0.25	0.6	3	150
11/29 am	Fluorourac	0.25	3.74	15	10
11/27 am	Fluorourac	0.25	1.95	8	50
11/27 am	Fluorourac	0.25	0.6	3	150
11/27 am	Fluorourac	0.25	0.5	3	250
11/26 am	Fluorourac	0.25	1.95	8	50
11/26 am	Fluorourac	0.25	1.8	8	200
11/26 am	Fluorourac	0.25	0.65	3	100
11/26 am	Fluorourac	0.25	0.625	3	125
11/26 am	Fluorourac	0.25	0.973	4	27
11/26 am	Fluorourac	0.25	0.973	4	27
11/25 am	Fluorourac	0.25	1.9	8	100
11/25 am	Fluorourac	0.25	1.8	8	200
11/25 am	Fluorourac	0.25	0.6	3	150
11/25 am	Fluorourac	0.25	1.9	8	100
11/24 am	Fluorourac	0.25	0.9	4	100
11/24 am	Fluorourac	0.25	0.9	4	100
11/24 am	Fluorourac	0.25	0.64	3	110
11/24 am	Fluorourac	0.25	1.9	8	100
11/23 am	Fluorourac	0.25	0.9	4	100
11/23 am	Fluorourac	0.25	0.9	4	100
11/23 am	Fluorourac	0.25	0.625	3	125
11/23 am	Fluorourac	0.25	4.35	18	150
11/22 am	Fluorourac	0.25	3.6	15	150
11/21 am	Fluorourac	0.25	0.9	4	100
11/21 am	Fluorourac	0.25	0.9	4	100

11/20 am	Fluorourac	0.25	0.9	4	100
11/20 am	Fluorourac	0.25	0.9	4	100
11/20 am	Fluorourac	0.25	0.66	3	90
11/20 am	Fluorourac	0.25	0.6	3	150
11/20 am	Fluorourac	0.25	0.625	3	125
11/1 am	Fluorourac	0.25	1.95	8	50
11/1 am	Fluorourac	0.25	3.48	14	20
11/1 am	Fluorourac	0.25	0.65	3	100
11/19 am	Fluorourac	0.25	2.4	10	100
11/19 am	Fluorourac	0.25	0.66	3	90
11/18 am	Fluorourac	0.25	0.625	3	125
11/18 am	Fluorourac	0.25	0.625	3	125
11/18 am	Fluorourac	0.25	1.9	8	100
11/17 am	Fluorourac	0.25	0.64	3	110
11/17 am	Fluorourac	0.25	1.9	8	100
11/17 am	Fluorourac	0.25	1.9	8	100
11/17 am	Fluorourac	0.25	0.64	3	110
11/14 am	Fluorourac	0.25	1.8	8	200
11/13 am	Fluorourac	0.25	0.7	3	50
11/13 am	Fluorourac	0.25	3.8	16	200
11/13 am	Fluorourac	0.25	0.6	3	150
11/13 am	Fluorourac	0.25	1.8	8	200
11/13 am	Fluorourac	0.25	3.74	15	10
11/12 am	Fluorourac	0.25	0.625	3	125
11/12 am	Fluorourac	0.25	0.937	4	63
11/12 am	Fluorourac	0.25	0.937	4	63
11/11 am	Fluorourac	0.25	1.9	8	100
11/11 am	Fluorourac	0.25	2.6	11	150
11/11 am	Fluorourac	0.25	0.8	4	200
11/11 am	Fluorourac	0.25	3.8	16	200
11/10 am	Fluorourac	0.25	1.9	8	100
11/10 am	Fluorourac	0.25	2.6	11	150
11/10 am	Fluorourac	0.25	1.9	8	100
10/9 am	Fluorourac	0.25	1.9	8	100
10/6 am	Fluorourac	0.25	0.72	3	30
10/5 am	Fluorourac	0.25	1.8	8	200
10/4 am	Fluorourac	0.25	1.8	8	200
10/4 am	Fluorourac	0.25	0.6	3	150
10/3 am	Fluorourac	0.25	0.937	4	63
10/3 am	Fluorourac	0.25	0.625	3	125
10/3 am	Fluorourac	0.25	0.937	4	63
10/30 am	Fluorourac	0.25	3.74	15	10
10/2 am	Fluorourac	0.25	1.95	8	50
10/28 am	Fluorourac	0.25	0.875	4	125
10/28 am	Fluorourac	0.25	0.875	4	125
10/28 am	Fluorourac	0.25	0.72	3	30
10/27 am	Fluorourac	0.25	0.625	3	125
10/26 am	Fluorourac	0.25	0.4	2	100
10/26 am	Fluorourac	0.25	0.625	3	125
10/26 am	Fluorourac	0.25	1.9	8	100
10/25 am	Fluorourac	0.25	0.64	3	110

10/25 am	Fluorourac	0.25	1.9	8	100
10/24 am	Fluorourac	0.25	2.2	9	50
10/23 am	Fluorourac	0.25	1.9	8	100
10/22 am	Fluorourac	0.25	1.9	8	100
10/22 am	Fluorourac	0.25	2.2	9	50
10/22 am	Fluorourac	0.25	0.625	3	125
10/22 am	Fluorourac	0.25	0.973	4	27
10/22 am	Fluorourac	0.25	0.973	4	27
10/22 am	Fluorourac	0.25	0.8	4	200
10/22 am	Fluorourac	0.25	0.6	3	150
10/21 pm	Fluorourac	0.25	0.68	3	70
10/21 pm	Fluorourac	0.25	2.06	9	190
10/21 am	Fluorourac	0.25	3.8	16	200
10/20 am	Fluorourac	0.25	1.8	8	200
10/20 am	Fluorourac	0.25	0.6	3	150
10/1 am	Fluorourac	0.25	1.95	8	50
10/1 am	Fluorourac	0.25	0.65	3	100
10/1 am	Fluorourac	0.25	0.8	4	200
10/19 pm	Fluorourac	0.25	0.875	4	125
10/19 pm	Fluorourac	0.25	0.875	4	125
10/19 am	Fluorourac	0.25	3.8	16	200
10/19 am	Fluorourac	0.25	1.8	8	200
10/19 am	Fluorourac	0.25	0.6	3	150
10/19 am	Fluorourac	0.25	0.6	3	150
10/18 am	Fluorourac	0.25	0.6	3	150
10/18 am	Fluorourac	0.25	0.625	3	125
10/16 am	Fluorourac	0.25	0.6	3	150
10/16 am	Fluorourac	0.25	3.74	15	10
10/15 am	Fluorourac	0.25	3.3	14	200
10/15 am	Fluorourac	0.25	0.7	3	50
10/14 am	Fluorourac	0.25	3.3	14	200
10/12 am	Fluorourac	0.25	0.625	3	125
10/10 am	Fluorourac	0.25	1.9	8	100
				1199	22697
9/30 am	Homotrim	1	1.5	2	0.5
9/15 am	Homotrim	1	1.5	2	0.5
9/14 am	Homotrim	1	1.5	2	0.5
9/13 am	Homotrim	1	1.5	2	0.5
9/12 am	Homotrim	1	1.5	2	0.5
9/11 am	Homotrim	1	1.5	2	0.5
10/6 am	Homotrim	1	1.5	2	0.5
10/5 pm	Homotrim	1	2.5	3	0.5
10/5 am	Homotrim	1	1.5	2	0.5
10/4 pm	Homotrim	1	2.5	3	0.5
10/4 am	Homotrim	1	1.5	2	0.5
10/3 pm	Homotrim	1	2.5	3	0.5
10/3 am	Homotrim	1	1.5	2	0.5
10/2 pm	Homotrim	1	2.5	3	0.5
10/2 am	Homotrim	1	1.5	2	0.5
10/1 pm	Homotrim	1	2.5	3	0.5
10/1 am	Homotrim	1	1.5	2	0.5

				39	8.5
11/30 pm	Oxaliplatin	50	180	4	20
11/29 am	Oxaliplatin	50	170	4	30
11/28 am	Oxaliplatin	50	180	4	20
11/27 am	Oxaliplatin	50	190	4	10
11/27 am	Oxaliplatin	50	125	3	25
11/27 am	Oxaliplatin	50	130	3	20
11/27 am	Oxaliplatin	50	140	3	10
11/27 am	Oxaliplatin	50	180	4	20
11/27 am	Oxaliplatin	50	130	3	20
11/26 am	Oxaliplatin	50	130	3	20
11/25 am	Oxaliplatin	50	160	4	40
11/25 am	Oxaliplatin	50	120	3	30
11/25 am	Oxaliplatin	50	180	4	20
11/23 am	Oxaliplatin	50	230	5	20
11/23 am	Oxaliplatin	50	135	3	15
11/23 am	Oxaliplatin	50	180	4	20
11/22 am	Oxaliplatin	50	190	4	10
11/20 am	Oxaliplatin	50	190	4	10
11/20 am	Oxaliplatin	50	130	3	20
11/20 am	Oxaliplatin	50	140	3	10
11/20 am	Oxaliplatin	50	25	1	25
11/19 am	Oxaliplatin	50	130	3	20
11/18 am	Oxaliplatin	50	180	4	20
11/18 am	Oxaliplatin	50	140	3	10
11/17 am	Oxaliplatin	50	190	4	10
11/16 am	Oxaliplatin	50	160	4	40
11/16 am	Oxaliplatin	50	120	3	30
11/16 am	Oxaliplatin	50	120	3	30
11/15 am	Oxaliplatin	50	180	4	20
				101	595
11/27 am	Gemcitabi	0.2	0.7	4	100
11/20 am	Gemcitabi	0.2	0.5	3	100
				7	200
11/30 pm	Gemcitabi	1	1.49	2	510
11/27 pm	Gemcitabi	1	1.47	2	530
11/22 pm	Gemcitabi	1	1.77	2	230
11/18 am	Gemcitabi	1	1.55	2	450
11/16 am	Gemcitabi	1	1.47	2	530
				10	2250
9/7 am	Methotrex	10	36	4	4
9/3 am	Methotrex	10	3.8	1	6.2
9/3 am	Methotrex	10	34.9	4	5.1
9/3 am	Methotrex	10	1.8	1	8.2
9/3 am	Methotrex	10	16.2	2	3.8
9/30 am	Methotrex	10	1.5	1	8.5
9/30 am	Methotrex	10	1.5	1	8.5
9/30 am	Methotrex	10	1.5	1	8.5
9/2 pm	Methotrex	10	1.5	1	8.5
9/2 pm	Methotrex	10	1.5	1	8.5
9/27 am	Methotrex	10	2.6	1	7.4

9/27 am	Methotrex	10	23.8	3	6.2
9/27 am	Methotrex	10	3.8	1	6.2
9/27 am	Methotrex	10	34.3	4	5.7
9/23 pm	Methotrex	10	1.5	1	8.5
9/23 pm	Methotrex	10	1.5	1	8.5
9/23 pm	Methotrex	10	1.5	1	8.5
9/20 am	Methotrex	10	55	6	5
9/16 am	Methotrex	10	5	1	5
9/16 am	Methotrex	10	45	5	5
9/15 am	Methotrex	10	3	1	7
9/15 am	Methotrex	10	8	1	2
9/15 am	Methotrex	10	7	1	3
9/15 am	Methotrex	10	1.5	1	8.5
9/15 am	Methotrex	10	1.5	1	8.5
9/15 am	Methotrex	10	1.5	1	8.5
9/13 pm	Methotrex	10	1.5	1	8.5
9/13 pm	Methotrex	10	1.5	1	8.5
9/13 pm	Methotrex	10	1.5	1	8.5
9/13 am	Methotrex	10	2.6	1	7.4
9/13 am	Methotrex	10	23.8	3	6.2
11/9 am	Methotrex	10	3	1	7
11/8 am	Methotrex	10	4.7	1	5.3
11/8 am	Methotrex	10	42.3	5	7.7
11/30 pm	Methotrex	10	66	7	4
11/30 am	Methotrex	10	1	1	9
11/30 am	Methotrex	10	1.5	1	8.5
11/30 am	Methotrex	10	1.5	1	8.5
11/30 am	Methotrex	10	1.4	1	8.6
11/30 am	Methotrex	10	1.3	1	8.7
11/2 am	Methotrex	10	5	1	5
11/2 am	Methotrex	10	45	5	5
11/29 am	Methotrex	10	3	1	7
11/29 am	Methotrex	10	42	5	8
11/27 pm	Methotrex	10	1	3	29
11/27 pm	Methotrex	10	1.5	1	8.5
11/23 pm	Methotrex	10	0.25	1	9.75
11/23 am	Methotrex	10	3.7	1	6.3
11/23 am	Methotrex	10	33.9	4	6.1
11/22 pm	Methotrex	10	0.25	1	9.75
11/18 pm	Methotrex	10	5	1	5
11/18 pm	Methotrex	10	15	2	5
11/17 am	Methotrex	10	5	1	5
11/17 am	Methotrex	10	45	5	5
11/15 pm	Methotrex	10	0.3	1	9.7
11/15 pm	Methotrex	10	29.7	3	0.3
11/14 pm	Methotrex	10	3	1	7
11/14 pm	Methotrex	10	57	6	3
11/13 pm	Methotrex	10	1	1	9
11/13 pm	Methotrex	10	1.5	1	8.5
11/13 pm	Methotrex	10	1.5	1	8.5
11/12 pm	Methotrex	10	1.8	1	8.2

11/12 pm	Methotrex	10	16.2	2	3.8
10/9 am	Methotrex	10	0.2	1	9.8
10/8 am	Methotrex	10	0.2	1	9.8
10/5 am	Methotrex	10	45	5	5
10/30 pm	Methotrex	10	1	1	9
10/30 pm	Methotrex	10	1.5	1	8.5
10/30 pm	Methotrex	10	1.5	1	8.5
10/29 am	Methotrex	10	5	1	5
10/29 am	Methotrex	10	27	3	3
10/29 am	Methotrex	10	1.4	1	8.6
10/29 am	Methotrex	10	1.4	1	8.6
10/29 am	Methotrex	10	1.3	1	8.7
10/28 am	Methotrex	10	3	1	7
10/28 am	Methotrex	10	32	4	8
10/27 am	Methotrex	10	55	6	5
10/19 am	Methotrex	10	45	5	5
10/19 am	Methotrex	10	45	5	5
10/19 am	Methotrex	10	5	1	5
10/19 am	Methotrex	10	5	1	5
10/15 pm	Methotrex	10	1.25	1	8.75
10/15 pm	Methotrex	10	1	1	9
10/15 pm	Methotrex	10	1.5	1	8.5
10/11 pm	Methotrex	10	22.7	3	7.3
10/11 pm	Methotrex	10	0.125	1	9.875
10/11 am	Methotrex	10	0.2	1	9.8
10/11 am	Methotrex	10	2.5	1	7.5
10/10 am	Methotrex	10	0.2	1	9.8
				172	647.125
9/9 am	Carboplati	50	370	8	30
9/7 am	Carboplati	50	340	7	10
9/4 pm	Carboplati	50	430	9	20
9/4 am	Carboplati	50	310	7	40
9/3 pm	Carboplati	50	360	8	40
9/3 am	Carboplati	50	249	5	1
9/30 pm	Carboplati	50	430	9	20
9/27 pm	Carboplati	50	420	9	30
9/27 am	Carboplati	50	230	5	20
9/25 am	Carboplati	50	92	2	8
9/24 am	Carboplati	50	92	2	8
9/17 am	Carboplati	50	580	12	20
9/17 am	Carboplati	50	340	7	10
9/11 am	Carboplati	50	438	9	12
9/11 am	Carboplati	50	538	11	12
11/2 am	Carboplati	50	5.5	1	44.5
11/2 am	Carboplati	50	55	2	45
11/2 am	Carboplati	50	120	3	30
11/29 pm	Carboplati	50	480	10	20
11/29 am	Carboplati	50	135	3	15
11/20 am	Carboplati	50	380	8	20
11/1 am	Carboplati	50	580	12	20
11/17 am	Carboplati	50	5	1	45

11/15 am	Carboplati	50	135	3	15
11/13 am	Carboplati	50	278	6	22
11/11 am	Carboplati	50	478	10	22
10/8 am	Carboplati	50	580	12	20
10/6 pm	Carboplati	50	480	10	20
				191	619.5
10/2 am	Mesna	0.4	0.6	2	200
10/2 am	Mesna	0.4	0.6	2	200
10/2 am	Mesna	0.4	0.6	2	200
10/1 am	Mesna	0.4	0.6	2	200
10/1 am	Mesna	0.4	0.6	2	200
10/1 am	Mesna	0.4	0.6	2	200
				12	1200
9/9 pm	Cisplatin	30	50	2	10
9/9 am	Cisplatin	30	40	2	20
9/9 am	Cisplatin	30	40	2	20
9/9 am	Cisplatin	30	40	2	20
9/9 am	Cisplatin	30	45	2	15
9/9 am	Cisplatin	30	40	2	20
9/9 am	Cisplatin	30	40	2	20
9/9 am	Cisplatin	30	50	2	10
9/8 pm	Cisplatin	30	45	2	15
9/8 pm	Cisplatin	30	50	2	10
9/8 am	Cisplatin	30	40	2	20
9/8 am	Cisplatin	30	40	2	20
9/8 am	Cisplatin	30	40	2	20
9/8 am	Cisplatin	30	40	2	20
9/8 am	Cisplatin	30	40	2	20
9/8 am	Cisplatin	30	40	2	20
9/7 pm	Cisplatin	30	50	2	10
9/7 am	Cisplatin	30	40	2	20
9/7 am	Cisplatin	30	40	2	20
9/7 am	Cisplatin	30	40	2	20
9/7 am	Cisplatin	30	40	2	20
9/7 am	Cisplatin	30	40	2	20
9/7 am	Cisplatin	30	80	3	10
9/7 am	Cisplatin	30	40	2	20
9/7 am	Cisplatin	30	40	2	20
9/7 am	Cisplatin	30	105	4	15
9/6 pm	Cisplatin	30	40	2	20
9/6 am	Cisplatin	30	40	2	20
9/6 am	Cisplatin	30	40	2	20
9/6 am	Cisplatin	30	40	2	20
9/6 am	Cisplatin	30	40	2	20
9/5 am	Cisplatin	30	40	2	20
9/5 am	Cisplatin	30	40	2	20
9/5 am	Cisplatin	30	40	2	20
9/5 am	Cisplatin	30	40	2	20
9/5 am	Cisplatin	30	40	2	20
9/5 am	Cisplatin	30	40	2	20
9/5 am	Cisplatin	30	40	2	20

9/4 am	Cisplatin	30	40	2	20
9/4 am	Cisplatin	30	40	2	20
9/4 am	Cisplatin	30	40	2	20
9/4 am	Cisplatin	30	40	2	20
9/4 am	Cisplatin	30	50	2	10
9/4 am	Cisplatin	30	40	2	20
9/4 am	Cisplatin	30	40	2	20
9/4 am	Cisplatin	30	40	2	20
9/4 am	Cisplatin	30	40	2	20
9/3 am	Cisplatin	30	40	2	20
9/3 am	Cisplatin	30	40	2	20
9/3 am	Cisplatin	30	40	2	20
9/3 am	Cisplatin	30	45	2	15
9/3 am	Cisplatin	30	50	2	10
9/3 am	Cisplatin	30	40	2	20
9/3 am	Cisplatin	30	40	2	20
9/3 am	Cisplatin	30	50	2	10
9/30 pm	Cisplatin	30	80	3	10
9/30 pm	Cisplatin	30	73	3	17
9/30 am	Cisplatin	30	80	3	10
9/30 am	Cisplatin	30	40	2	20
9/30 am	Cisplatin	30	40	2	20
9/30 am	Cisplatin	30	50	2	10
9/2 am	Cisplatin	30	45	2	15
9/2 am	Cisplatin	30	50	2	10
9/2 am	Cisplatin	30	50	2	10
9/2 am	Cisplatin	30	40	2	20
9/2 am	Cisplatin	30	40	2	20
9/2 am	Cisplatin	30	40	2	20
9/29 am	Cisplatin	30	40	2	20
9/29 am	Cisplatin	30	40	2	20
9/29 am	Cisplatin	30	50	2	10
9/29 am	Cisplatin	30	50	2	10
9/29 am	Cisplatin	30	50	2	10
9/29 am	Cisplatin	30	40	2	20
9/28 pm	Cisplatin	30	50	2	10
9/28 pm	Cisplatin	30	50	2	10
9/28 pm	Cisplatin	30	40	2	20
9/28 pm	Cisplatin	30	40	2	20
9/28 am	Cisplatin	30	40	2	20
9/28 am	Cisplatin	30	50	2	10
9/27 am	Cisplatin	30	50	2	10
9/27 am	Cisplatin	30	40	2	20
9/27 am	Cisplatin	30	40	2	20
9/27 am	Cisplatin	30	50	2	10
9/27 am	Cisplatin	30	40	2	20
9/27 am	Cisplatin	30	40	2	20
9/27 am	Cisplatin	30	40	2	20
9/26 pm	Cisplatin	30	40	2	20
9/26 am	Cisplatin	30	40	2	20
9/26 am	Cisplatin	30	40	2	20

9/26 am	Cisplatin	30	40	2	20
9/26 am	Cisplatin	30	40	2	20
9/25 am	Cisplatin	30	40	2	20
9/25 am	Cisplatin	30	40	2	20
9/25 am	Cisplatin	30	40	2	20
9/25 am	Cisplatin	30	40	2	20
9/25 am	Cisplatin	30	50	2	10
9/25 am	Cisplatin	30	40	2	20
9/25 am	Cisplatin	30	40	2	20
9/25 am	Cisplatin	30	40	2	20
9/25 am	Cisplatin	30	40	2	20
9/25 am	Cisplatin	30	100	4	20
9/24 am	Cisplatin	30	40	2	20
9/24 am	Cisplatin	30	40	2	20
9/24 am	Cisplatin	30	40	2	20
9/24 am	Cisplatin	30	27	1	3
9/24 am	Cisplatin	30	40	2	20
9/24 am	Cisplatin	30	50	2	10
9/24 am	Cisplatin	30	40	2	20
9/24 am	Cisplatin	30	40	2	20
9/23 pm	Cisplatin	30	27	1	3
9/23 am	Cisplatin	30	40	2	20
9/23 am	Cisplatin	30	40	2	20
9/23 am	Cisplatin	30	40	2	20
9/23 am	Cisplatin	30	40	2	20
9/23 am	Cisplatin	30	40	2	20
9/23 am	Cisplatin	30	40	2	20
9/23 am	Cisplatin	30	40	2	20
9/23 am	Cisplatin	30	100	4	20
9/22 pm	Cisplatin	30	27	1	3
9/22 am	Cisplatin	30	40	2	20
9/22 am	Cisplatin	30	50	2	10
9/22 am	Cisplatin	30	50	2	10
9/21 pm	Cisplatin	30	27	1	3
9/21 am	Cisplatin	30	40	2	20
9/21 am	Cisplatin	30	40	2	20
9/21 am	Cisplatin	30	40	2	20
9/21 am	Cisplatin	30	40	2	20
9/21 am	Cisplatin	30	40	2	20
9/21 am	Cisplatin	30	40	2	20
9/21 am	Cisplatin	30	40	2	20
9/21 am	Cisplatin	30	40	2	20
9/21 am	Cisplatin	30	40	2	20
9/21 am	Cisplatin	30	40	2	20
9/21 am	Cisplatin	30	40	2	20
9/21 am	Cisplatin	30	40	2	20
9/21 am	Cisplatin	30	50	2	10
9/21 am	Cisplatin	30	50	2	10
9/21 am	Cisplatin	30	80	3	10
9/20 am	Cisplatin	30	40	2	20
9/20 am	Cisplatin	30	25	1	5

9/20 am	Cisplatin	30	40	2	20
9/20 am	Cisplatin	30	40	2	20
9/20 am	Cisplatin	30	40	2	20
9/20 am	Cisplatin	30	40	2	20
9/20 am	Cisplatin	30	40	2	20
9/20 am	Cisplatin	30	25	1	5
9/20 am	Cisplatin	30	40	2	20
9/20 am	Cisplatin	30	40	2	20
9/20 am	Cisplatin	30	40	2	20
9/20 am	Cisplatin	30	40	2	20
9/20 am	Cisplatin	30	27	1	3
9/20 am	Cisplatin	30	40	2	20
9/20 am	Cisplatin	30	40	2	20
9/20 am	Cisplatin	30	40	2	20
9/20 am	Cisplatin	30	40	2	20
9/20 am	Cisplatin	30	100	4	20
9/1 am	Cisplatin	30	40	2	20
9/1 am	Cisplatin	30	40	2	20
9/19 am	Cisplatin	30	40	2	20
9/19 am	Cisplatin	30	25	1	5
9/19 am	Cisplatin	30	40	2	20
9/19 am	Cisplatin	30	40	2	20
9/19 am	Cisplatin	30	40	2	20
9/19 am	Cisplatin	30	40	2	20
9/19 am	Cisplatin	30	40	2	20
9/19 am	Cisplatin	30	40	2	20
9/19 am	Cisplatin	30	40	2	20
9/19 am	Cisplatin	30	40	2	20
9/19 am	Cisplatin	30	40	2	20
9/19 am	Cisplatin	30	40	2	20
9/19 am	Cisplatin	30	40	2	20
9/19 am	Cisplatin	30	40	2	20
9/19 am	Cisplatin	30	40	2	20
9/19 am	Cisplatin	30	40	2	20
9/19 am	Cisplatin	30	40	2	20
9/19 am	Cisplatin	30	40	2	20
9/19 am	Cisplatin	30	40	2	20
9/19 am	Cisplatin	30	40	2	20
9/19 am	Cisplatin	30	40	2	20
9/19 am	Cisplatin	30	40	2	20
9/19 am	Cisplatin	30	40	2	20
9/19 am	Cisplatin	30	50	2	10
9/19 am	Cisplatin	30	50	2	10
9/19 am	Cisplatin	30	80	3	10
9/18 am	Cisplatin	30	25	1	5
9/18 am	Cisplatin	30	40	2	20
9/18 am	Cisplatin	30	40	2	20
9/18 am	Cisplatin	30	40	2	20
9/18 am	Cisplatin	30	40	2	20
9/18 am	Cisplatin	30	40	2	20
9/18 am	Cisplatin	30	40	2	20

9/18 am	Cisplatin	30	50	2	10
9/17 pm	Cisplatin	30	44	2	16
9/17 am	Cisplatin	30	40	2	20
9/17 am	Cisplatin	30	40	2	20
9/17 am	Cisplatin	30	40	2	20
9/17 am	Cisplatin	30	40	2	20
9/17 am	Cisplatin	30	40	2	20
9/17 am	Cisplatin	30	45	2	15
9/17 am	Cisplatin	30	40	2	20
9/17 am	Cisplatin	30	80	3	10
9/16 am	Cisplatin	30	40	2	20
9/16 am	Cisplatin	30	40	2	20
9/16 am	Cisplatin	30	40	2	20
9/16 am	Cisplatin	30	50	2	10
9/16 am	Cisplatin	30	100	4	20
9/15 am	Cisplatin	30	40	2	20
9/15 am	Cisplatin	30	40	2	20
9/15 am	Cisplatin	30	103.9	4	16.1
9/15 am	Cisplatin	30	40	2	20
9/15 am	Cisplatin	30	50	2	10
9/14 pm	Cisplatin	30	100	4	20
9/14 am	Cisplatin	30	40	2	20
9/14 am	Cisplatin	30	40	2	20
9/14 am	Cisplatin	30	40	2	20
9/14 am	Cisplatin	30	40	2	20
9/13 am	Cisplatin	30	40	2	20
9/13 am	Cisplatin	30	50	2	10
9/13 am	Cisplatin	30	40	2	20
9/13 am	Cisplatin	30	40	2	20
9/13 am	Cisplatin	30	40	2	20
9/13 am	Cisplatin	30	50	2	10
9/12 am	Cisplatin	30	40	2	20
9/12 am	Cisplatin	30	50	2	10
9/12 am	Cisplatin	30	40	2	20
9/12 am	Cisplatin	30	50	2	10
9/12 am	Cisplatin	30	50	2	10
9/12 am	Cisplatin	30	40	2	20
9/12 am	Cisplatin	30	40	2	20
9/12 am	Cisplatin	30	40	2	20
9/12 am	Cisplatin	30	40	2	20
9/12 am	Cisplatin	30	40	2	20
9/12 am	Cisplatin	30	40	2	20
9/11 am	Cisplatin	30	40	2	20
9/11 am	Cisplatin	30	50	2	10
9/11 am	Cisplatin	30	40	2	20
9/11 am	Cisplatin	30	40	2	20
9/11 am	Cisplatin	30	40	2	20
9/11 am	Cisplatin	30	50	2	10
9/11 am	Cisplatin	30	40	2	20
9/11 am	Cisplatin	30	40	2	20
9/11 am	Cisplatin	30	40	2	20

9/11 am	Cisplatin	30	40	2	20
9/11 am	Cisplatin	30	40	2	20
9/11 am	Cisplatin	30	50	2	10
9/11 am	Cisplatin	30	50	2	10
9/11 am	Cisplatin	30	40	2	20
9/10 pm	Cisplatin	30	40	2	20
9/10 am	Cisplatin	30	40	2	20
9/10 am	Cisplatin	30	40	2	20
9/10 am	Cisplatin	30	50	2	10
9/10 am	Cisplatin	30	40	2	20
9/10 am	Cisplatin	30	40	2	20
9/10 am	Cisplatin	30	50	2	10
9/10 am	Cisplatin	30	45	2	15
9/10 am	Cisplatin	30	40	2	20
9/10 am	Cisplatin	30	110	4	10
12/1 am	Cisplatin	30	40	2	20
12/1 am	Cisplatin	30	35	2	25
12/1 am	Cisplatin	30	40	2	20
12/1 am	Cisplatin	30	40	2	20
11/9 am	Cisplatin	30	40	2	20
11/9 am	Cisplatin	30	40	2	20
11/9 am	Cisplatin	30	40	2	20
11/9 am	Cisplatin	30	40	2	20
11/9 am	Cisplatin	30	40	2	20
11/9 am	Cisplatin	30	95	4	25
11/9 am	Cisplatin	30	50	2	10
11/9 am	Cisplatin	30	45	2	15
11/9 am	Cisplatin	30	40	2	20
11/8 am	Cisplatin	30	40	2	20
11/8 am	Cisplatin	30	40	2	20
11/8 am	Cisplatin	30	160	6	20
11/8 am	Cisplatin	30	34.4	2	25.6
11/8 am	Cisplatin	30	40	2	20
11/7 am	Cisplatin	30	40	2	20
11/7 am	Cisplatin	30	40	2	20
11/7 am	Cisplatin	30	40	2	20
11/7 am	Cisplatin	30	40	2	20
11/7 am	Cisplatin	30	40	2	20
11/7 am	Cisplatin	30	50	2	10
11/7 am	Cisplatin	30	68.8	3	21.2
11/7 am	Cisplatin	30	40	2	20
11/6 pm	Cisplatin	30	40	2	20
11/6 am	Cisplatin	30	35	2	25
11/6 am	Cisplatin	30	40	2	20
11/6 am	Cisplatin	30	40	2	20
11/6 am	Cisplatin	30	40	2	20
11/6 am	Cisplatin	30	40	2	20
11/6 am	Cisplatin	30	40	2	20
11/6 am	Cisplatin	30	50	2	10
11/6 am	Cisplatin	30	40	2	20
11/5 pm	Cisplatin	30	102.9	4	17.1

11/5 am	Cisplatin	30	35	2	25
11/5 am	Cisplatin	30	40	2	20
11/5 am	Cisplatin	30	40	2	20
11/5 am	Cisplatin	30	40	2	20
11/5 am	Cisplatin	30	40	2	20
11/5 am	Cisplatin	30	40	2	20
11/5 am	Cisplatin	30	40	2	20
11/5 am	Cisplatin	30	50	2	10
11/5 am	Cisplatin	30	40	2	20
11/5 am	Cisplatin	30	40	2	20
11/5 am	Cisplatin	30	40	2	20
11/4 am	Cisplatin	30	35	2	25
11/4 am	Cisplatin	30	40	2	20
11/4 am	Cisplatin	30	40	2	20
11/4 am	Cisplatin	30	50	2	10
11/4 am	Cisplatin	30	50	2	10
11/4 am	Cisplatin	30	45	2	15
11/4 am	Cisplatin	30	50	2	10
11/4 am	Cisplatin	30	100	4	20
11/3 am	Cisplatin	30	40	2	20
11/3 am	Cisplatin	30	40	2	20
11/3 am	Cisplatin	30	50	2	10
11/3 am	Cisplatin	30	40	2	20
11/3 am	Cisplatin	30	40	2	20
11/30 pm	Cisplatin	30	40	2	20
11/30 am	Cisplatin	30	35	2	25
11/30 am	Cisplatin	30	40	2	20
11/30 am	Cisplatin	30	45	2	15
11/30 am	Cisplatin	30	40	2	20
11/30 am	Cisplatin	30	50	2	10
11/30 am	Cisplatin	30	50	2	10
11/30 am	Cisplatin	30	40	2	20
11/30 am	Cisplatin	30	95	4	25
11/30 am	Cisplatin	30	45	2	15
11/30 am	Cisplatin	30	0.9	1	29.1
11/30 am	Cisplatin	30	9	1	21
11/30 am	Cisplatin	30	80	3	10
11/30 am	Cisplatin	30	10	1	20
11/2 am	Cisplatin	30	45	2	15
11/2 am	Cisplatin	30	40	2	20
11/2 am	Cisplatin	30	40	2	20
11/2 am	Cisplatin	30	50	2	10
11/29 pm	Cisplatin	30	50	2	10
11/29 pm	Cisplatin	30	10	1	20
11/29 am	Cisplatin	30	40	2	20
11/29 am	Cisplatin	30	50	2	10
11/28 am	Cisplatin	30	40	2	20
11/28 am	Cisplatin	30	40	2	20
11/28 am	Cisplatin	30	40	2	20
11/28 am	Cisplatin	30	45	2	15
11/28 am	Cisplatin	30	50	2	10

11/28 am	Cisplatin	30	50	2	10
11/28 am	Cisplatin	30	50	2	10
11/28 am	Cisplatin	30	102.9	4	17.1
11/27 am	Cisplatin	30	40	2	20
11/27 am	Cisplatin	30	40	2	20
11/27 am	Cisplatin	30	50	2	10
11/27 am	Cisplatin	30	40	2	20
11/27 am	Cisplatin	30	50	2	10
11/27 am	Cisplatin	30	50	2	10
11/27 am	Cisplatin	30	40	2	20
11/26 am	Cisplatin	30	40	2	20
11/26 am	Cisplatin	30	40	2	20
11/26 am	Cisplatin	30	40	2	20
11/26 am	Cisplatin	30	50	2	10
11/26 am	Cisplatin	30	34.9	2	25.1
11/26 am	Cisplatin	30	40	2	20
11/26 am	Cisplatin	30	100	4	20
11/25 am	Cisplatin	30	50	2	10
11/25 am	Cisplatin	30	40	2	20
11/25 am	Cisplatin	30	40	2	20
11/25 am	Cisplatin	30	68	3	22
11/25 am	Cisplatin	30	45	2	15
11/24 am	Cisplatin	30	50	2	10
11/24 am	Cisplatin	30	50	2	10
11/24 am	Cisplatin	30	40	2	20
11/24 am	Cisplatin	30	40	2	20
11/24 am	Cisplatin	30	50	2	10
11/24 am	Cisplatin	30	40	2	20
11/24 am	Cisplatin	30	40	2	20
11/23 am	Cisplatin	30	50	2	10
11/23 am	Cisplatin	30	40	2	20
11/23 am	Cisplatin	30	100	4	20
11/23 am	Cisplatin	30	50	2	10
11/23 am	Cisplatin	30	50	2	10
11/22 pm	Cisplatin	30	100	4	20
11/22 am	Cisplatin	30	40	2	20
11/22 am	Cisplatin	30	50	2	10
11/21 am	Cisplatin	30	50	2	10
11/21 am	Cisplatin	30	40	2	20
11/21 am	Cisplatin	30	40	2	20
11/21 am	Cisplatin	30	50	2	10
11/21 am	Cisplatin	30	50	2	10
11/21 am	Cisplatin	30	40	2	20
11/20 am	Cisplatin	30	40	2	20
11/20 am	Cisplatin	30	40	2	20
11/20 am	Cisplatin	30	50	2	10
11/20 am	Cisplatin	30	50	2	10
11/20 am	Cisplatin	30	67	3	23
11/1 am	Cisplatin	30	40	2	20
11/1 am	Cisplatin	30	45	2	15
11/1 am	Cisplatin	30	40	2	20

11/1 am	Cisplatin	30	45	2	15
11/19 am	Cisplatin	30	40	2	20
11/19 am	Cisplatin	30	40	2	20
11/19 am	Cisplatin	30	170	6	10
11/18 am	Cisplatin	30	40	2	20
11/18 am	Cisplatin	30	40	2	20
11/18 am	Cisplatin	30	50	2	10
11/18 am	Cisplatin	30	40	2	20
11/18 am	Cisplatin	30	50	2	10
11/18 am	Cisplatin	30	80	3	10
11/17 pm	Cisplatin	30	103.9	4	16.1
11/17 am	Cisplatin	30	40	2	20
11/17 am	Cisplatin	30	50	2	10
11/17 am	Cisplatin	30	45	2	15
11/17 am	Cisplatin	30	40	2	20
11/17 am	Cisplatin	30	40	2	20
11/17 am	Cisplatin	30	50	2	10
11/17 am	Cisplatin	30	45	2	15
11/17 am	Cisplatin	30	50	2	10
11/17 am	Cisplatin	30	40	2	20
11/16 pm	Cisplatin	30	27	1	3
11/16 am	Cisplatin	30	50	2	10
11/16 am	Cisplatin	30	45	2	15
11/16 am	Cisplatin	30	40	2	20
11/16 am	Cisplatin	30	40	2	20
11/16 am	Cisplatin	30	86	3	4
11/16 am	Cisplatin	30	40	2	20
11/16 am	Cisplatin	30	40	2	20
11/15 pm	Cisplatin	30	27	1	3
11/15 pm	Cisplatin	30	100	4	20
11/15 am	Cisplatin	30	40	2	20
11/15 am	Cisplatin	30	40	2	20
11/15 am	Cisplatin	30	50	2	10
11/15 am	Cisplatin	30	40	2	20
11/15 am	Cisplatin	30	20	1	10
11/15 am	Cisplatin	30	40	2	20
11/14 pm	Cisplatin	30	27	1	3
11/14 am	Cisplatin	30	50	2	10
11/14 am	Cisplatin	30	40	2	20
11/14 am	Cisplatin	30	40	2	20
11/14 am	Cisplatin	30	40	2	20
11/14 am	Cisplatin	30	40	2	20
11/14 am	Cisplatin	30	40	2	20
11/14 am	Cisplatin	30	40	2	20
11/14 am	Cisplatin	30	50	2	10
11/14 am	Cisplatin	30	45	2	15
11/14 am	Cisplatin	30	45	2	15
11/13 pm	Cisplatin	30	27	1	3
11/13 am	Cisplatin	30	50	2	10
11/13 am	Cisplatin	30	40	2	20
11/13 am	Cisplatin	30	45	2	15

11/13 am	Cisplatin	30	40	2	20
11/13 am	Cisplatin	30	40	2	20
11/13 am	Cisplatin	30	40	2	20
11/13 am	Cisplatin	30	40	2	20
11/13 am	Cisplatin	30	35	2	25
11/13 am	Cisplatin	30	40	2	20
11/13 am	Cisplatin	30	40	2	20
11/13 am	Cisplatin	30	40	2	20
11/13 am	Cisplatin	30	50	2	10
11/13 am	Cisplatin	30	40	2	20
11/13 am	Cisplatin	30	40	2	20
11/13 am	Cisplatin	30	45	2	15
11/13 am	Cisplatin	30	50	2	10
11/12 pm	Cisplatin	30	27	1	3
11/12 pm	Cisplatin	30	110	4	10
11/12 am	Cisplatin	30	45	2	15
11/12 am	Cisplatin	30	40	2	20
11/12 am	Cisplatin	30	40	2	20
11/12 am	Cisplatin	30	40	2	20
11/12 am	Cisplatin	30	35	2	25
11/12 am	Cisplatin	30	40	2	20
11/12 am	Cisplatin	30	40	2	20
11/12 am	Cisplatin	30	40	2	20
11/12 am	Cisplatin	30	50	2	10
11/12 am	Cisplatin	30	40	2	20
11/12 am	Cisplatin	30	40	2	20
11/12 am	Cisplatin	30	80	3	10
11/12 am	Cisplatin	30	100	4	20
11/11 am	Cisplatin	30	35	2	25
11/11 am	Cisplatin	30	40	2	20
11/11 am	Cisplatin	30	50	2	10
11/11 am	Cisplatin	30	40	2	20
11/11 am	Cisplatin	30	110	4	10
11/11 am	Cisplatin	30	40	2	20
11/11 am	Cisplatin	30	45	2	15
11/11 am	Cisplatin	30	40	2	20
11/11 am	Cisplatin	30	40	2	20
11/11 am	Cisplatin	30	40	2	20
11/11 am	Cisplatin	30	40	2	20
11/10 pm	Cisplatin	30	40	2	20
11/10 pm	Cisplatin	30	80	3	10
11/10 am	Cisplatin	30	40	2	20
11/10 am	Cisplatin	30	40	2	20
11/10 am	Cisplatin	30	40	2	20
11/10 am	Cisplatin	30	50	2	10
11/10 am	Cisplatin	30	40	2	20
11/10 am	Cisplatin	30	48.5	2	11.5
11/10 am	Cisplatin	30	40	2	20
10/9 am	Cisplatin	30	40	2	20
10/9 am	Cisplatin	30	50	2	10
10/9 am	Cisplatin	30	50	2	10

10/9 am	Cisplatin	30	40	2	20
10/9 am	Cisplatin	30	40	2	20
10/8 pm	Cisplatin	30	50	2	10
10/5 am	Cisplatin	30	40	2	20
10/5 am	Cisplatin	30	40	2	20
10/5 am	Cisplatin	30	40	2	20
10/5 am	Cisplatin	30	50	2	10
10/4 pm	Cisplatin	30	110	4	10
10/4 am	Cisplatin	30	40	2	20
10/4 am	Cisplatin	30	40	2	20
10/4 am	Cisplatin	30	40	2	20
10/4 am	Cisplatin	30	40	2	20
10/3 am	Cisplatin	30	50	2	10
10/3 am	Cisplatin	30	40	2	20
10/3 am	Cisplatin	30	40	2	20
10/3 am	Cisplatin	30	40	2	20
10/3 am	Cisplatin	30	40	2	20
10/3 am	Cisplatin	30	40	2	20
10/3 am	Cisplatin	30	40	2	20
10/3 am	Cisplatin	30	50	2	10
10/3 am	Cisplatin	30	40	2	20
10/3 am	Cisplatin	30	40	2	20
10/3 am	Cisplatin	30	40	2	20
10/3 am	Cisplatin	30	40	2	20
10/3 am	Cisplatin	30	40	2	20
10/31 am	Cisplatin	30	40	2	20
10/31 am	Cisplatin	30	40	2	20
10/31 am	Cisplatin	30	40	2	20
10/31 am	Cisplatin	30	50	2	10
10/31 am	Cisplatin	30	40	2	20
10/31 am	Cisplatin	30	40	2	20
10/31 am	Cisplatin	30	40	2	20
10/31 am	Cisplatin	30	50	2	10
10/31 am	Cisplatin	30	40	2	20
10/31 am	Cisplatin	30	50	2	10
10/31 am	Cisplatin	30	40	2	20
10/31 am	Cisplatin	30	34.9	2	25.1
10/31 am	Cisplatin	30	40	2	20
10/30 pm	Cisplatin	30	50	2	10
10/30 am	Cisplatin	30	40	2	20
10/30 am	Cisplatin	30	40	2	20
10/30 am	Cisplatin	30	40	2	20
10/30 am	Cisplatin	30	40	2	20
10/30 am	Cisplatin	30	40	2	20
10/30 am	Cisplatin	30	40	2	20
10/30 am	Cisplatin	30	50	2	10
10/30 am	Cisplatin	30	40	2	20
10/30 am	Cisplatin	30	50	2	10
10/30 am	Cisplatin	30	40	2	20
10/30 am	Cisplatin	30	40	2	20
10/30 am	Cisplatin	30	40	2	20

10/30 am	Cisplatin	30	40	2	20
10/30 am	Cisplatin	30	68	3	22
10/2 am	Cisplatin	30	50	2	10
10/2 am	Cisplatin	30	40	2	20
10/2 am	Cisplatin	30	40	2	20
10/2 am	Cisplatin	30	40	2	20
10/2 am	Cisplatin	30	40	2	20
10/2 am	Cisplatin	30	40	2	20
10/2 am	Cisplatin	30	40	2	20
10/2 am	Cisplatin	30	40	2	20
10/2 am	Cisplatin	30	50	2	10
10/2 am	Cisplatin	30	40	2	20
10/2 am	Cisplatin	30	40	2	20
10/2 am	Cisplatin	30	50	2	10
10/2 am	Cisplatin	30	40	2	20
10/29 am	Cisplatin	30	40	2	20
10/29 am	Cisplatin	30	40	2	20
10/29 am	Cisplatin	30	50	2	10
10/29 am	Cisplatin	30	40	2	20
10/29 am	Cisplatin	30	50	2	10
10/29 am	Cisplatin	30	50	2	10
10/29 am	Cisplatin	30	100	4	20
10/28 am	Cisplatin	30	50	2	10
10/28 am	Cisplatin	30	40	2	20
10/28 am	Cisplatin	30	40	2	20
10/28 am	Cisplatin	30	50	2	10
10/27 am	Cisplatin	30	50	2	10
10/27 am	Cisplatin	30	50	2	10
10/27 am	Cisplatin	30	50	2	10
10/27 am	Cisplatin	30	103.9	4	16.1
10/27 am	Cisplatin	30	40	2	20
10/27 am	Cisplatin	30	50	2	10
10/27 am	Cisplatin	30	50	2	10
10/26 pm	Cisplatin	30	100	4	20
10/26 am	Cisplatin	30	50	2	10
10/26 am	Cisplatin	30	50	2	10
10/26 am	Cisplatin	30	40	2	20
10/26 am	Cisplatin	30	50	2	10
10/26 am	Cisplatin	30	40	2	20
10/26 am	Cisplatin	30	40	2	20
10/26 am	Cisplatin	30	40	2	20
10/26 am	Cisplatin	30	40	2	20
10/26 am	Cisplatin	30	50	2	10
10/25 am	Cisplatin	30	40	2	20
10/24 pm	Cisplatin	30	100	4	20
10/24 am	Cisplatin	30	50	2	10
10/24 am	Cisplatin	30	40	2	20
10/24 am	Cisplatin	30	40	2	20
10/24 am	Cisplatin	30	40	2	20
10/24 am	Cisplatin	30	40	2	20
10/24 am	Cisplatin	30	100	4	20

10/23 pm	Cisplatin	30	40	2	20
10/23 pm	Cisplatin	30	100	4	20
10/23 am	Cisplatin	30	50	2	10
10/23 am	Cisplatin	30	40	2	20
10/23 am	Cisplatin	30	40	2	20
10/23 am	Cisplatin	30	40	2	20
10/23 am	Cisplatin	30	40	2	20
10/23 am	Cisplatin	30	40	2	20
10/23 am	Cisplatin	30	50	2	10
10/23 am	Cisplatin	30	40	2	20
10/23 am	Cisplatin	30	45	2	15
10/23 am	Cisplatin	30	40	2	20
10/22 pm	Cisplatin	30	40	2	20
10/22 pm	Cisplatin	30	100	4	20
10/22 pm	Cisplatin	30	100	4	20
10/22 am	Cisplatin	30	50	2	10
10/22 am	Cisplatin	30	40	2	20
10/22 am	Cisplatin	30	27	1	3
10/22 am	Cisplatin	30	40	2	20
10/22 am	Cisplatin	30	40	2	20
10/22 am	Cisplatin	30	40	2	20
10/22 am	Cisplatin	30	40	2	20
10/22 am	Cisplatin	30	80	3	10
10/21 am	Cisplatin	30	40	2	20
10/21 am	Cisplatin	30	40	2	20
10/21 am	Cisplatin	30	40	2	20
10/21 am	Cisplatin	30	40	2	20
10/21 am	Cisplatin	30	40	2	20
10/21 am	Cisplatin	30	40	2	20
10/21 am	Cisplatin	30	27	1	3
10/21 am	Cisplatin	30	40	2	20
10/21 am	Cisplatin	30	40	2	20
10/21 am	Cisplatin	30	40	2	20
10/20 am	Cisplatin	30	40	2	20
10/20 am	Cisplatin	30	40	2	20
10/20 am	Cisplatin	30	40	2	20
10/20 am	Cisplatin	30	40	2	20
10/20 am	Cisplatin	30	40	2	20
10/20 am	Cisplatin	30	27	1	3
10/20 am	Cisplatin	30	40	2	20
10/20 am	Cisplatin	30	40	2	20
10/20 am	Cisplatin	30	48.5	2	11.5
10/1 am	Cisplatin	30	50	2	10
10/1 am	Cisplatin	30	40	2	20
10/1 am	Cisplatin	30	40	2	20
10/1 am	Cisplatin	30	40	2	20
10/1 am	Cisplatin	30	50	2	10
10/1 am	Cisplatin	30	40	2	20
10/1 am	Cisplatin	30	40	2	20
10/1 am	Cisplatin	30	40	2	20
10/1 am	Cisplatin	30	40	2	20

10/1 am	Cisplatin	30	40	2	20
10/1 am	Cisplatin	30	50	2	10
10/1 am	Cisplatin	30	45	2	15
10/1 am	Cisplatin	30	40	2	20
10/19 am	Cisplatin	30	40	2	20
10/19 am	Cisplatin	30	40	2	20
10/19 am	Cisplatin	30	40	2	20
10/19 am	Cisplatin	30	40	2	20
10/19 am	Cisplatin	30	40	2	20
10/19 am	Cisplatin	30	27	1	3
10/19 am	Cisplatin	30	40	2	20
10/19 am	Cisplatin	30	40	2	20
10/19 am	Cisplatin	30	95	4	25
10/19 am	Cisplatin	30	40	2	20
10/19 am	Cisplatin	30	50	2	10
10/18 am	Cisplatin	30	40	2	20
10/18 am	Cisplatin	30	40	2	20
10/18 am	Cisplatin	30	40	2	20
10/18 am	Cisplatin	30	40	2	20
10/18 am	Cisplatin	30	40	2	20
10/18 am	Cisplatin	30	27	1	3
10/18 am	Cisplatin	30	40	2	20
10/17 am	Cisplatin	30	40	2	20
10/17 am	Cisplatin	30	40	2	20
10/17 am	Cisplatin	30	40	2	20
10/17 am	Cisplatin	30	40	2	20
10/16 pm	Cisplatin	30	44	2	16
10/16 pm	Cisplatin	30	34.4	2	25.6
10/16 am	Cisplatin	30	40	2	20
10/16 am	Cisplatin	30	40	2	20
10/16 am	Cisplatin	30	140	5	10
10/16 am	Cisplatin	30	40	2	20
10/16 am	Cisplatin	30	40	2	20
10/16 am	Cisplatin	30	40	2	20
10/16 am	Cisplatin	30	40	2	20
10/16 am	Cisplatin	30	40	2	20
10/16 am	Cisplatin	30	50	2	10
10/16 am	Cisplatin	30	40	2	20
10/16 am	Cisplatin	30	40	2	20
10/16 am	Cisplatin	30	40	2	20
10/15 am	Cisplatin	30	75	3	15
10/15 am	Cisplatin	30	40	2	20
10/15 am	Cisplatin	30	40	2	20
10/15 am	Cisplatin	30	40	2	20
10/15 am	Cisplatin	30	40	2	20
10/15 am	Cisplatin	30	40	2	20
10/15 am	Cisplatin	30	68.8	3	21.2
10/15 am	Cisplatin	30	40	2	20
10/14 pm	Cisplatin	30	75	3	15
10/14 am	Cisplatin	30	40	2	20
10/14 am	Cisplatin	30	40	2	20

10/14 am	Cisplatin	30	40	2	20
10/14 am	Cisplatin	30	40	2	20
10/14 am	Cisplatin	30	40	2	20
10/14 am	Cisplatin	30	40	2	20
10/13 am	Cisplatin	30	40	2	20
10/13 am	Cisplatin	30	40	2	20
10/13 am	Cisplatin	30	50	2	10
10/13 am	Cisplatin	30	40	2	20
10/13 am	Cisplatin	30	40	2	20
10/13 am	Cisplatin	30	100	4	20
10/13 am	Cisplatin	30	65	3	25
10/13 am	Cisplatin	30	45	2	15
10/12 am	Cisplatin	30	40	2	20
10/12 am	Cisplatin	30	40	2	20
10/12 am	Cisplatin	30	40	2	20
10/12 am	Cisplatin	30	40	2	20
10/12 am	Cisplatin	30	40	2	20
10/12 am	Cisplatin	30	40	2	20
10/12 am	Cisplatin	30	40	2	20
10/12 am	Cisplatin	30	40	2	20
10/12 am	Cisplatin	30	45	2	15
10/12 am	Cisplatin	30	40	2	20
10/11 am	Cisplatin	30	35	2	25
10/11 am	Cisplatin	30	40	2	20
10/11 am	Cisplatin	30	40	2	20
10/11 am	Cisplatin	30	40	2	20
10/11 am	Cisplatin	30	40	2	20
10/11 am	Cisplatin	30	40	2	20
10/11 am	Cisplatin	30	40	2	20
10/11 am	Cisplatin	30	40	2	20
10/11 am	Cisplatin	30	40	2	20
10/11 am	Cisplatin	30	100	4	20
10/10 am	Cisplatin	30	40	2	20
10/10 am	Cisplatin	30	50	2	10
10/10 am	Cisplatin	30	50	2	10
10/10 am	Cisplatin	30	40	2	20
10/10 am	Cisplatin	30	40	2	20
10/10 am	Cisplatin	30	35	2	25
10/10 am	Cisplatin	30	40	2	20
10/10 am	Cisplatin	30	40	2	20
				1556	12929.4
9/30 am	Calcium F	100	10	1	90
9/30 am	Calcium F	100	10	1	90
9/30 am	Calcium F	100	10	1	90
9/30 am	Calcium F	100	5	1	95
9/2 am	Calcium F	100	5	1	95
9/29 pm	Calcium F	100	10	1	90
9/28 pm	Calcium F	100	10	1	90
9/28 pm	Calcium F	100	10	1	90
9/28 pm	Calcium F	100	10	1	90
9/28 pm	Calcium F	100	10	1	90

9/28 pm	Calcium F	100	5	1	95
9/23 am	Calcium F	100	580	6	20
9/21 am	Calcium F	100	80	1	20
9/21 am	Calcium F	100	80	1	20
9/21 am	Calcium F	100	80	1	20
9/21 am	Calcium F	100	80	1	20
9/21 am	Calcium F	100	80	1	20
9/21 am	Calcium F	100	80	1	20
9/21 am	Calcium F	100	80	1	20
9/19 pm	Calcium F	100	15	1	85
9/19 am3	Calcium F	100	15	1	85
9/19 am	Calcium F	100	15	1	85
9/19 am	Calcium F	100	15	1	85
9/19 am	Calcium F	100	5	1	95
9/19 am	Calcium F	100	5	1	95
9/18 am3	Calcium F	100	15	1	85
9/18 am	Calcium F	100	15	1	85
9/18 am	Calcium F	100	15	1	85
9/18 am	Calcium F	100	5	1	95
9/17 am	Calcium F	100	20	1	80
9/17 am	Calcium F	100	20	1	80
9/17 am	Calcium F	100	20	1	80
9/17 am	Calcium F	100	20	1	80
9/17 am	Calcium F	100	20	1	80
9/17 am	Calcium F	100	680	7	20
9/14 am	Calcium F	100	80	2	120
11/9 am	Calcium F	100	5	1	95
11/9 am	Calcium F	100	5	1	95
11/9 am	Calcium F	100	5	1	95
11/9 am	Calcium F	100	5	1	95
11/9 am	Calcium F	100	5	1	95
11/9 am	Calcium F	100	80	1	20
11/8 am	Calcium F	100	5	1	95
11/7 am	Calcium F	100	5	1	95
11/6 am	Calcium F	100	5	1	95
11/5 pm	Calcium F	100	750	8	50
11/5 am	Calcium F	100	15	1	85
11/5 am	Calcium F	100	15	1	85
11/5 am	Calcium F	100	15	1	85
11/5 am	Calcium F	100	15	1	85
11/5 am	Calcium F	100	5	1	95
11/5 am	Calcium F	100	680	7	20
11/4 am	Calcium F	100	15	1	85
11/4 am	Calcium F	100	15	1	85
11/4 am	Calcium F	100	15	1	85
11/4 am	Calcium F	100	15	1	85
11/4 am	Calcium F	100	5	1	95
11/4 am	Calcium F	100	5	1	95
11/3 am	Calcium F	100	5	1	95
11/3 am	Calcium F	100	80	1	20
11/3 am	Calcium F	100	80	1	20

11/3 am	Calcium F	100	80	1	20
11/3 am	Calcium F	100	80	1	20
11/2 pm	Calcium F	100	80	1	20
11/2 pm	Calcium F	100	80	1	20
11/2 pm	Calcium F	100	80	1	20
11/2 pm	Calcium F	100	80	1	20
11/2 am	Calcium F	100	5	1	95
11/29 am	Calcium F	100	80	1	20
11/29 am	Calcium F	100	5	1	95
11/28 am	Calcium F	100	5	1	95
11/26 am	Calcium F	100	5	1	95
11/26 am	Calcium F	100	5	1	95
11/26 am	Calcium F	100	5	1	95
11/26 am	Calcium F	100	5	1	95
11/26 am	Calcium F	100	5	1	95
11/26 am	Calcium F	100	5	1	95
11/26 am	Calcium F	100	5	1	95
11/26 am	Calcium F	100	14	1	86
11/26 am	Calcium F	100	14	1	86
11/26 am	Calcium F	100	14	1	86
11/26 am	Calcium F	100	14	1	86
11/24 am	Calcium F	100	50	1	50
11/24 am	Calcium F	100	50	1	50
11/24 am	Calcium F	100	50	1	50
11/24 am	Calcium F	100	50	1	50
11/24 am	Calcium F	100	80	1	20
11/24 am	Calcium F	100	80	1	20
11/24 am	Calcium F	100	80	1	20
11/24 am	Calcium F	100	80	1	20
11/22 pm	Calcium F	100	50	1	50
11/22 pm	Calcium F	100	50	1	50
11/22 pm	Calcium F	100	50	1	50
11/22 pm	Calcium F	100	50	1	50
11/22 am	Calcium F	100	5	1	95
11/22 am	Calcium F	100	50	1	50
11/22 am	Calcium F	100	50	1	50
11/22 am	Calcium F	100	50	1	50
11/22 am	Calcium F	100	50	1	50
11/21 am	Calcium F	100	5	1	95
11/21 am	Calcium F	100	5	1	95
11/21 am	Calcium F	100	5	1	95
11/21 am	Calcium F	100	50	1	50
11/21 am	Calcium F	100	50	1	50
11/21 am	Calcium F	100	50	1	50
11/21 am	Calcium F	100	50	1	50
11/20 pm	Calcium F	100	50	1	50
11/20 pm	Calcium F	100	50	1	50
11/20 pm	Calcium F	100	50	1	50
11/20 pm	Calcium F	100	50	1	50
11/20 am	Calcium F	100	5	1	95
11/20 am	Calcium F	100	15	1	85

11/20 am	Calcium F	100	5	1	95
11/20 am	Calcium F	100	5	1	95
11/20 am	Calcium F	100	50	1	50
11/20 am	Calcium F	100	50	1	50
11/20 am	Calcium F	100	50	1	50
11/20 am	Calcium F	100	50	1	50
11/20 am	Calcium F	100	330	4	70
11/1 am	Calcium F	100	580	6	20
11/19 am	Calcium F	100	80	1	20
11/19 am	Calcium F	100	5	1	95
11/19 am	Calcium F	100	15	1	85
11/19 am	Calcium F	100	15	1	85
11/19 am	Calcium F	100	15	1	85
11/19 am	Calcium F	100	5	1	95
11/19 am	Calcium F	100	5	1	95
11/19 am	Calcium F	100	6.7	1	93.3
11/19 am	Calcium F	100	5	1	95
11/19 am	Calcium F	100	15	1	85
11/19 am	Calcium F	100	330	4	70
11/19 am	Calcium F	100	6.7	1	93.3
11/19 am	Calcium F	100	6.7	1	93.3
11/19 am	Calcium F	100	6.7	1	93.3
11/18 am	Calcium F	100	6.7	1	93.3
11/18 am	Calcium F	100	10	1	90
11/18 am	Calcium F	100	10	1	90
11/18 am	Calcium F	100	10	1	90
11/18 am	Calcium F	100	10	1	90
11/18 am	Calcium F	100	5	1	95
11/18 am	Calcium F	100	5	1	95
11/17 am	Calcium F	100	6.7	1	93.3
11/17 am	Calcium F	100	10	1	90
11/17 am	Calcium F	100	10	1	90
11/17 am	Calcium F	100	10	1	90
11/17 am	Calcium F	100	10	1	90
11/17 am	Calcium F	100	5	1	95
11/17 am	Calcium F	100	5	1	95
11/17 am	Calcium F	100	6.7	1	93.3
11/17 am	Calcium F	100	6.7	1	93.3
11/17 am	Calcium F	100	6.7	1	93.3
11/17 am	Calcium F	100	640	7	60
11/16 am	Calcium F	100	5	1	95
11/16 am	Calcium F	100	6.7	1	93.3
11/16 am	Calcium F	100	6.7	1	93.3
11/16 am	Calcium F	100	6.7	1	93.3
11/15 am	Calcium F	100	6.7	1	93.3
11/15 am	Calcium F	100	6.7	1	93.3
11/15 am	Calcium F	100	6.7	1	93.3
11/15 am	Calcium F	100	6.7	1	93.3
11/14 am	Calcium F	100	6.7	1	93.3
11/13 am	Calcium F	100	5	1	95
11/13 am	Calcium F	100	6.7	1	93.3

11/13 am	Calcium F	100	6.7	1	93.3
11/13 am	Calcium F	100	6.7	1	93.3
11/13 am	Calcium F	100	6.7	1	93.3
11/12 pm	Calcium F	100	360	4	40
11/12 am	Calcium F	100	5	1	95
11/12 am	Calcium F	100	5	1	95
11/12 am	Calcium F	100	5	1	95
11/12 am	Calcium F	100	5	1	95
11/12 am	Calcium F	100	5	1	95
11/12 am	Calcium F	100	5	1	95
11/12 am	Calcium F	100	14	1	86
11/11 am	Calcium F	100	80	1	20
11/10 am	Calcium F	100	5	1	95
11/10 am	Calcium F	100	5	1	95
11/10 am	Calcium F	100	5	1	95
11/10 am	Calcium F	100	5	1	95
11/10 am	Calcium F	100	640	7	60
10/9 am	Calcium F	100	5	1	95
10/9 am	Calcium F	100	80	1	20
10/9 am	Calcium F	100	80	1	20
10/9 am	Calcium F	100	80	1	20
10/9 am	Calcium F	100	80	1	20
10/8 pm	Calcium F	100	15	1	85
10/8 am	Calcium F	100	5	1	95
10/8 am	Calcium F	100	15	1	85
10/8 am	Calcium F	100	15	1	85
10/8 am	Calcium F	100	15	1	85
10/6 pm	Calcium F	100	15	1	85
10/6 am	Calcium F	100	5	1	95
10/6 am	Calcium F	100	15	1	85
10/6 am	Calcium F	100	15	1	85
10/6 am	Calcium F	100	15	1	85
10/31 am	Calcium F	100	10	1	90
10/31 am	Calcium F	100	10	1	90
10/31 am	Calcium F	100	10	1	90
10/31 am	Calcium F	100	10	1	90
10/30 am	Calcium F	100	10	1	90
10/30 am	Calcium F	100	10	1	90
10/30 am	Calcium F	100	10	1	90
10/30 am	Calcium F	100	10	1	90
10/30 am	Calcium F	100	5	1	95
10/30 am	Calcium F	100	5	1	95
10/29 pm	Calcium F	100	80	1	20
10/29 am	Calcium F	100	750	8	50
10/27 am	Calcium F	100	750	8	50
10/25 am	Calcium F	100	5	1	95
10/23 am	Calcium F	100	550	6	50
10/23 am	Calcium F	100	5	1	95
10/21 pm	Calcium F	100	680	7	20
10/21 am	Calcium F	100	15	1	85
10/21 am	Calcium F	100	15	1	85

10/21 am	Calcium F	100	15	1	85
10/21 am	Calcium F	100	15	1	85
10/21 am	Calcium F	100	5	1	95
10/21 am	Calcium F	100	15	1	85
10/21 am	Calcium F	100	15	1	85
10/21 am	Calcium F	100	15	1	85
10/21 am	Calcium F	100	15	1	85
10/21 am	Calcium F	100	5	1	95
10/18 pm	Calcium F	100	5	1	95
10/15 pm	Calcium F	100	5	1	95
10/14 pm	Calcium F	100	10	1	90
10/14 pm	Calcium F	100	10	1	90
10/14 pm	Calcium F	100	10	1	90
10/14 pm	Calcium F	100	10	1	90
10/14 pm	Calcium F	100	5	1	95
10/12 pm	Calcium F	100	10	1	90
10/12 pm	Calcium F	100	10	1	90
10/12 pm	Calcium F	100	10	1	90
10/12 pm	Calcium F	100	10	1	90
10/12 pm	Calcium F	100	5	1	95
10/11 pm	Calcium F	100	5	1	95
10/10 am	Calcium F	100	5	1	95
10/10 am	Calcium F	100	80	1	20
10/10 am	Calcium F	100	80	1	20
10/10 am	Calcium F	100	80	1	20
10/10 am	Calcium F	100	80	1	20
				316	17939.3
12/1 am	Etoposide/	40	70	2	10
12/1 am	Etoposide/	40	50	2	30
11/9 am	Etoposide/	40	50	2	30
11/9 am	Etoposide/	40	90	3	30
11/9 am	Etoposide/	40	30	1	10
11/9 am	Etoposide/	40	70	2	10
11/8 pm	Etoposide/	40	100	3	20
11/8 am	Etoposide/	40	90	3	30
11/8 am	Etoposide/	40	90	3	30
11/8 am	Etoposide/	40	70	2	10
11/7 pm	Etoposide/	40	100	3	20
11/7 am	Etoposide/	40	90	3	30
11/7 am	Etoposide/	40	70	2	10
11/7 am	Etoposide/	40	70	2	10
11/6 pm	Etoposide/	40	100	3	20
11/6 pm	Etoposide/	40	100	3	20
11/6 am	Etoposide/	40	90	3	30
11/6 am	Etoposide/	40	70	2	10
11/6 am	Etoposide/	40	30	1	10
11/6 am	Etoposide/	40	70	2	10
11/6 am	Etoposide/	40	70	2	10
11/6 am	Etoposide/	40	100	3	20
11/5 am	Etoposide/	40	70	2	10
11/5 am	Etoposide/	40	70	2	10

11/5 am	Etoposide/	40	20	1	20
11/5 am	Etoposide/	40	70	2	10
11/5 am	Etoposide/	40	70	2	10
11/4 pm	Etoposide/	40	20	1	20
11/4 am	Etoposide/	40	20	1	20
11/4 am	Etoposide/	40	70	2	10
11/4 am	Etoposide/	40	70	2	10
11/3 pm	Etoposide/	40	20	1	20
11/3 am	Etoposide/	40	20	1	20
11/3 am	Etoposide/	40	70	2	10
11/30 pm	Etoposide/	40	50	2	30
11/30 am	Etoposide/	40	70	2	10
11/30 am	Etoposide/	40	30	1	10
11/30 am	Etoposide/	40	20	1	20
11/30 am	Etoposide/	40	60	2	20
11/30 am	Etoposide/	40	75	2	5
11/30 am	Etoposide/	40	75	2	5
11/30 am	Etoposide/	40	50	2	30
11/2 pm	Etoposide/	40	20	1	20
11/2 am	Etoposide/	40	30	1	10
11/2 am	Etoposide/	40	80	3	40
11/2 am	Etoposide/	40	20	1	20
11/29 am	Etoposide/	40	20	1	20
11/29 am	Etoposide/	40	85	3	35
11/27 pm	Etoposide/	40	100	3	20
11/27 am	Etoposide/	40	70	2	10
11/27 am	Etoposide/	40	30	1	10
11/26 pm	Etoposide/	40	38	1	2
11/26 am	Etoposide/	40	70	2	10
11/25 pm	Etoposide/	40	85	3	35
11/25 am	Etoposide/	40	60	2	20
11/24 am	Etoposide/	40	10	1	30
11/24 am	Etoposide/	40	60	2	20
11/23 am	Etoposide/	40	10	1	30
11/2311/23	Etoposide/	40	30	1	10
11/2311/23	Etoposide/	40	60	2	20
11/22 pm	Etoposide/	40	10	1	30
11/22 pm	Etoposide/	40	100	3	20
11/22 pm	Etoposide/	40	100	3	20
11/22 am	Etoposide/	40	70	2	10
11/21 am	Etoposide/	40	10	1	30
11/20 pm	Etoposide/	40	100	3	20
11/20 pm	Etoposide/	40	100	3	20
11/20 am	Etoposide/	40	30	1	10
11/1 am	Etoposide/	40	70	2	10
11/1 am	Etoposide/	40	100	3	20
11/1 am	Etoposide/	40	90	3	30
11/19 am	Etoposide/	40	20	1	20
11/19 am	Etoposide/	40	100	3	20
11/19 am	Etoposide/	40	38	1	2
11/18 am	Etoposide/	40	20	1	20

11/18 am	Etoposide/	40	90	3	30
11/18 am	Etoposide/	40	100	3	20
11/17 am	Etoposide/	40	90	3	30
11/17 am	Etoposide/	40	70	3	50
11/17 am	Etoposide/	40	100	3	20
11/17 am	Etoposide/	40	100	3	20
11/17 am	Etoposide/	40	70	2	10
11/17 am	Etoposide/	40	20	1	20
11/16 am	Etoposide/	40	30	1	10
11/16 am	Etoposide/	40	130	4	30
11/16 am	Etoposide/	40	28	1	12
11/16 am	Etoposide/	40	70	2	10
11/16 am	Etoposide/	40	90	3	30
11/15 am	Etoposide/	40	60	2	20
11/15 am	Etoposide/	40	130	4	30
11/15 am	Etoposide/	40	28	1	12
11/15 am	Etoposide/	40	70	2	10
11/15 am	Etoposide/	40	100	3	20
11/15 am	Etoposide/	40	100	3	20
11/14 am	Etoposide/	40	30	1	10
11/14 am	Etoposide/	40	70	2	10
11/14 am	Etoposide/	40	100	3	20
11/14 am	Etoposide/	40	90	3	30
11/14 am	Etoposide/	40	60	2	20
11/14 am	Etoposide/	40	130	4	30
11/13 pm	Etoposide/	40	30	1	10
11/13 pm	Etoposide/	40	100	3	20
11/13 pm	Etoposide/	40	50	2	30
11/13 am	Etoposide/	40	30	1	10
11/13 am	Etoposide/	40	70	2	10
11/13 am	Etoposide/	40	100	3	20
11/13 am	Etoposide/	40	100	3	20
11/13 am	Etoposide/	40	100	3	20
11/13 am	Etoposide/	40	90	3	30
11/13 am	Etoposide/	40	60	2	20
11/13 am	Etoposide/	40	130	4	30
11/13 am	Etoposide/	40	28	1	12
11/12 pm	Etoposide/	40	130	4	30
11/12 pm	Etoposide/	40	70	2	10
11/12 pm	Etoposide/	40	38	1	2
11/12 am	Etoposide/	40	30	1	10
11/12 am	Etoposide/	40	50	2	30
11/12 am	Etoposide/	40	30	1	10
11/12 am	Etoposide/	40	38	1	2
11/12 am	Etoposide/	40	90	3	30
11/11 am	Etoposide/	40	30	1	10
11/11 am	Etoposide/	40	50	2	30
11/11 am	Etoposide/	40	50	2	30
11/10 am	Etoposide/	40	90	3	30
11/10 am	Etoposide/	40	50	2	30
11/10 am	Etoposide/	40	50	2	30

11/10 am	Etoposide/	40	70	2	10
10/9 am	Etoposide/	40	50	2	30
10/9 am	Etoposide/	40	100	3	20
10/8 am	Etoposide/	40	100	3	20
10/7 pm	Etoposide/	40	100	3	20
10/7 pm	Etoposide/	40	100	3	20
10/7 am	Etoposide/	40	100	3	20
10/7 am	Etoposide/	40	100	3	20
10/7 am	Etoposide/	40	50	2	30
10/4 am	Etoposide/	40	100	3	20
10/31 am	Etoposide/	40	70	2	10
10/31 am	Etoposide/	40	100	3	20
10/31 am	Etoposide/	40	90	3	30
10/30 pm	Etoposide/	40	50	2	30
10/30 pm	Etoposide/	40	100	3	20
10/30 am	Etoposide/	40	70	2	10
10/30 am	Etoposide/	40	30	1	10
10/30 am	Etoposide/	40	90	3	30
10/2 pm	Etoposide/	40	100	3	20
10/2 am	Etoposide/	40	30	1	10
10/29 am	Etoposide/	40	30	1	10
10/29 am	Etoposide/	40	76	2	4
10/29 am	Etoposide/	40	50	2	30
10/28 am	Etoposide/	40	60	2	20
10/28 am	Etoposide/	40	70	2	10
10/28 am	Etoposide/	40	100	3	20
10/28 am	Etoposide/	40	50	2	30
10/28 am	Etoposide/	40	100	3	20
10/28 am	Etoposide/	40	50	2	30
10/27 am	Etoposide/	40	60	2	20
10/27 am	Etoposide/	40	50	2	30
10/27 am	Etoposide/	40	100	3	20
10/27 am	Etoposide/	40	50	2	30
10/26 am	Etoposide/	40	30	1	10
10/26 am	Etoposide/	40	100	3	20
10/26 am	Etoposide/	40	50	2	30
10/26 am	Etoposide/	40	60	2	20
10/26 am	Etoposide/	40	100	3	20
10/26 am	Etoposide/	40	100	3	20
10/26 am	Etoposide/	40	75	2	5
10/26 am	Etoposide/	40	75	2	5
10/25 am	Etoposide/	40	75	2	5
10/25 am	Etoposide/	40	75	2	5
10/24 am	Etoposide/	40	90	3	30
10/24 am	Etoposide/	40	100	3	20
10/24 am	Etoposide/	40	100	3	20
10/24 am	Etoposide/	40	75	2	5
10/24 am	Etoposide/	40	75	2	5
10/23 am	Etoposide/	40	50	2	30
10/23 am	Etoposide/	40	100	3	20
10/23 am	Etoposide/	40	100	3	20

10/23 am	Etoposide/	40	90	3	30
10/23 am	Etoposide/	40	60	2	20
10/23 am	Etoposide/	40	70	2	10
10/22 pm	Etoposide/	40	90	3	30
10/22 pm	Etoposide/	40	100	3	20
10/22 pm	Etoposide/	40	100	3	20
10/22 pm	Etoposide/	40	100	3	20
10/22 am	Etoposide/	40	50	2	30
10/22 am	Etoposide/	40	100	3	20
10/22 am	Etoposide/	40	100	3	20
10/22 am	Etoposide/	40	60	2	20
10/22 am	Etoposide/	40	70	2	10
10/21 pm	Etoposide/	40	17	1	23
10/21 pm	Etoposide/	40	50	2	30
10/21 am	Etoposide/	40	90	3	30
10/21 am	Etoposide/	40	30	1	10
10/21 am	Etoposide/	40	60	2	20
10/21 am	Etoposide/	40	70	2	10
10/20 am	Etoposide/	40	90	3	30
10/20 am	Etoposide/	40	30	1	10
10/1 am	Etoposide/	40	30	1	10
10/19 am	Etoposide/	40	30	1	10
10/19 am	Etoposide/	40	60	2	20
10/19 am	Etoposide/	40	90	3	30
10/18 pm	Etoposide/	40	60	2	20
10/18 am	Etoposide/	40	60	2	20
10/17 am	Etoposide/	40	60	2	20
10/17 am	Etoposide/	40	60	2	20
10/17 am	Etoposide/	40	70	2	10
10/16 pm	Etoposide/	40	100	3	20
10/16 am	Etoposide/	40	60	2	20
10/16 am	Etoposide/	40	60	2	20
10/16 am	Etoposide/	40	30	1	10
10/16 am	Etoposide/	40	50	2	30
10/16 am	Etoposide/	40	70	2	10
10/16 am	Etoposide/	40	30	1	10
10/15 pm	Etoposide/	40	70	2	10
10/15 am	Etoposide/	40	100	3	20
10/15 am	Etoposide/	40	70	2	10
10/15 am	Etoposide/	40	50	2	30
10/15 am	Etoposide/	40	100	3	20
10/14 am	Etoposide/	40	28	1	12
10/14 am	Etoposide/	40	100	3	20
10/14 am	Etoposide/	40	50	2	30
10/14 am	Etoposide/	40	70	2	10
10/14 am	Etoposide/	40	50	2	30
10/13 am	Etoposide/	40	28	1	12
10/13 am	Etoposide/	40	70	2	10
10/12 am	Etoposide/	40	28	1	12
10/12 am	Etoposide/	40	50	2	30
10/12 am	Etoposide/	40	100	3	20

10/12 am	Etoposide/	40	70	2	10
10/11 am	Etoposide/	40	28	1	12
10/11 am	Etoposide/	40	70	2	10
10/11 am	Etoposide/	40	50	2	30
10/11 am	Etoposide/	40	100	3	20
10/10 am	Etoposide/	40	50	2	30
10/10 am	Etoposide/	40	100	3	20
10/10 am	Etoposide/	40	70	2	10
				509	4469
9/9 am	Etoposide/	0.1	0.05	1	50
9/9 am	Etoposide/	0.1	0.07	1	30
9/9 am	Etoposide/	0.1	0.05	1	50
9/9 am	Etoposide/	0.1	0.05	1	50
9/9 am	Etoposide/	0.1	0.07	1	30
9/9 am	Etoposide/	0.1	0.08	1	20
9/9 am	Etoposide/	0.1	0.07	1	30
9/9 am	Etoposide/	0.1	0.07	1	30
9/9 am	Etoposide/	0.1	0.06	1	40
9/8 am	Etoposide/	0.1	0.05	1	50
9/8 am	Etoposide/	0.1	0.07	1	30
9/8 am	Etoposide/	0.1	0.05	1	50
9/8 am	Etoposide/	0.1	0.05	1	50
9/8 am	Etoposide/	0.1	0.07	1	30
9/8 am	Etoposide/	0.1	0.08	1	20
9/8 am	Etoposide/	0.1	0.07	1	30
9/8 am	Etoposide/	0.1	0.07	1	30
9/8 am	Etoposide/	0.1	0.06	1	40
9/7 pm	Etoposide/	0.1	0.129	2	71
9/7 am	Etoposide/	0.1	0.05	1	50
9/7 am	Etoposide/	0.1	0.07	1	30
9/7 am	Etoposide/	0.1	0.15	2	50
9/7 am	Etoposide/	0.1	0.07	1	30
9/7 am	Etoposide/	0.1	0.07	1	30
9/6 pm	Etoposide/	0.1	0.129	2	71
9/6 pm	Etoposide/	0.1	0.15	2	50
9/6 am	Etoposide/	0.1	0.07	1	30
9/6 am	Etoposide/	0.1	0.053	1	47
9/6 am	Etoposide/	0.1	0.05	1	50
9/6 am	Etoposide/	0.1	0.05	1	50
9/6 am	Etoposide/	0.1	0.125	2	75
9/6 am	Etoposide/	0.1	0.05	1	50
9/5 am	Etoposide/	0.1	0.07	1	30
9/5 am	Etoposide/	0.1	0.06	1	40
9/5 am	Etoposide/	0.1	0.05	1	50
9/5 am	Etoposide/	0.1	0.053	1	47
9/5 am	Etoposide/	0.1	0.05	1	50
9/5 am	Etoposide/	0.1	0.05	1	50
9/4 pm	Etoposide/	0.1	0.053	1	47
9/4 am	Etoposide/	0.1	0.02	1	80
9/4 am	Etoposide/	0.1	0.06	1	40
9/4 am	Etoposide/	0.1	0.05	1	50

9/4 am	Etoposide/	0.1	0.05	1	50
9/4 am	Etoposide/	0.1	0.12	2	80
9/4 am	Etoposide/	0.1	0.07	1	30
9/3 am	Etoposide/	0.1	0.02	1	80
9/3 am	Etoposide/	0.1	0.06	1	40
9/3 am	Etoposide/	0.1	0.05	1	50
9/3 am	Etoposide/	0.1	0.12	2	80
9/3 am	Etoposide/	0.1	0.08	1	20
9/3 am	Etoposide/	0.1	0.09	1	10
9/3 am	Etoposide/	0.1	0.05	1	50
9/3 am	Etoposide/	0.1	0.05	1	50
9/3 am	Etoposide/	0.1	0.05	1	50
9/30 pm	Etoposide/	0.1	0.094	1	6
9/30 pm	Etoposide/	0.1	0.094	1	6
9/30 pm	Etoposide/	0.1	0.094	1	6
9/30 pm	Etoposide/	0.1	0.094	1	6
9/30 pm	Etoposide/	0.1	0.07	1	30
9/30 pm	Etoposide/	0.1	0.07	1	30
9/30 pm	Etoposide/	0.1	0.07	1	30
9/30 pm	Etoposide/	0.1	0.07	1	30
9/30 pm	Etoposide/	0.1	0.07	1	30
9/30 pm	Etoposide/	0.1	0.07	1	30
9/30 pm	Etoposide/	0.1	0.05	1	50
9/2 pm	Etoposide/	0.1	0.05	1	50
9/2 am	Etoposide/	0.1	0.05	1	50
9/2 am	Etoposide/	0.1	0.02	1	80
9/2 am	Etoposide/	0.1	0.12	2	80
9/2 am	Etoposide/	0.1	0.08	1	20
9/2 am	Etoposide/	0.1	0.09	1	10
9/2 am	Etoposide/	0.1	0.08	1	20
9/2 am	Etoposide/	0.1	0.02	1	80
9/2 am	Etoposide/	0.1	0.05	1	50
9/28 pm	Etoposide/	0.1	0.03	1	70
9/28 am	Etoposide/	0.1	0.04	1	60
9/28 am	Etoposide/	0.1	0.12	2	80
9/27 am	Etoposide/	0.1	0.03	1	70
9/27 am	Etoposide/	0.1	0.04	1	60
9/27 am	Etoposide/	0.1	0.07	1	30
9/27 am	Etoposide/	0.1	0.05	1	50
9/27 am	Etoposide/	0.1	0.03	1	70
9/26 pm	Etoposide/	0.1	0.06	1	40
9/26 pm	Etoposide/	0.1	0.06	1	40
9/26 pm	Etoposide/	0.1	0.07	1	30
9/26 am	Etoposide/	0.1	0.04	1	60
9/26 am	Etoposide/	0.1	0.05	1	50
9/26 am	Etoposide/	0.1	0.07	1	30
9/26 am	Etoposide/	0.1	0.03	1	70
9/26 am	Etoposide/	0.1	0.12	2	80
9/26 am	Etoposide/	0.1	0.04	1	60
9/26 am	Etoposide/	0.1	0.05	1	50
9/25 am	Etoposide/	0.1	0.05	1	50

9/25 am	Etoposide/	0.1	0.069	1	31
9/25 am	Etoposide/	0.1	0.12	2	80
9/25 am	Etoposide/	0.1	0.04	1	60
9/25 am	Etoposide/	0.1	0.03	1	70
9/25 am	Etoposide/	0.1	0.07	1	30
9/25 am	Etoposide/	0.1	0.04	1	60
9/25 am	Etoposide/	0.1	0.05	1	50
9/25 am	Etoposide/	0.1	0.05	1	50
9/24 pm	Etoposide/	0.1	0.06	1	40
9/24 pm	Etoposide/	0.1	0.05	1	50
9/24 am	Etoposide/	0.1	0.03	1	70
9/24 am	Etoposide/	0.1	0.05	1	50
9/24 am	Etoposide/	0.1	0.069	1	31
9/24 am	Etoposide/	0.1	0.12	2	80
9/24 am	Etoposide/	0.1	0.04	1	60
9/23 pm	Etoposide/	0.1	0.06	1	40
9/23 pm	Etoposide/	0.1	0.05	1	50
9/23 am	Etoposide/	0.1	0.03	1	70
9/23 am	Etoposide/	0.1	0.05	1	50
9/23 am	Etoposide/	0.1	0.069	1	31
9/23 am	Etoposide/	0.1	0.04	1	60
9/22 am	Etoposide/	0.1	0.07	1	30
9/22 am	Etoposide/	0.1	0.06	1	40
9/22 am	Etoposide/	0.1	0.06	1	40
9/21 pm	Etoposide/	0.1	0.045	1	55
9/21 pm	Etoposide/	0.1	0.065	1	35
9/21 pm	Etoposide/	0.1	0.045	1	55
9/21 am	Etoposide/	0.1	0.07	1	30
9/21 am	Etoposide/	0.1	0.07	1	30
9/21 am	Etoposide/	0.1	0.06	1	40
9/21 am	Etoposide/	0.1	0.125	2	75
9/21 am	Etoposide/	0.1	0.04	1	60
9/21 am	Etoposide/	0.1	0.06	1	40
9/21 am	Etoposide/	0.1	0.06	1	40
9/20 am	Etoposide/	0.1	0.06	1	40
9/20 am	Etoposide/	0.1	0.08	1	20
9/20 am	Etoposide/	0.1	0.07	1	30
9/20 am	Etoposide/	0.1	0.07	1	30
9/20 am	Etoposide/	0.1	0.06	1	40
9/20 am	Etoposide/	0.1	0.06	1	40
9/20 am	Etoposide/	0.1	0.05	1	50
9/20 am	Etoposide/	0.1	0.04	1	60
9/20 am	Etoposide/	0.1	0.06	1	40
9/1 am	Etoposide/	0.1	0.08	1	20
9/1 am	Etoposide/	0.1	0.09	1	10
9/19 pm	Etoposide/	0.1	0.045	1	55
9/19 pm	Etoposide/	0.1	0.045	1	55
9/19 pm	Etoposide/	0.1	0.125	2	75
9/19 am	Etoposide/	0.1	0.08	1	20
9/19 am	Etoposide/	0.1	0.06	1	40
9/19 am	Etoposide/	0.1	0.06	1	40

9/19 am	Etoposide/	0.1	0.07	1	30
9/19 am	Etoposide/	0.1	0.07	1	30
9/19 am	Etoposide/	0.1	0.05	1	50
9/19 am	Etoposide/	0.1	0.06	1	40
9/19 am	Etoposide/	0.1	0.04	1	60
9/19 am	Etoposide/	0.1	0.05	1	50
9/19 am	Etoposide/	0.1	0.04	1	60
9/19 am	Etoposide/	0.1	0.06	1	40
9/18 am	Etoposide/	0.1	0.08	1	20
9/18 am	Etoposide/	0.1	0.06	1	40
9/18 am	Etoposide/	0.1	0.07	1	30
9/18 am	Etoposide/	0.1	0.045	1	55
9/18 am	Etoposide/	0.1	0.05	1	50
9/18 am	Etoposide/	0.1	0.06	1	40
9/17 pm	Etoposide/	0.1	0.06	1	40
9/17 am	Etoposide/	0.1	0.05	1	50
9/17 am	Etoposide/	0.1	0.065	1	35
9/17 am	Etoposide/	0.1	0.045	1	55
9/17 am	Etoposide/	0.1	0.06	1	40
9/17 am	Etoposide/	0.1	0.06	1	40
9/16 am	Etoposide/	0.1	0.05	1	50
9/16 am	Etoposide/	0.1	0.05	1	50
9/16 am	Etoposide/	0.1	0.06	1	40
9/16 am	Etoposide/	0.1	0.05	1	50
9/15 am	Etoposide/	0.1	0.05	1	50
9/15 am	Etoposide/	0.1	0.06	1	40
9/15 am	Etoposide/	0.1	0.05	1	50
9/15 am	Etoposide/	0.1	0.05	1	50
9/14 am	Etoposide/	0.1	0.05	1	50
9/14 am	Etoposide/	0.1	0.065	1	35
9/14 am	Etoposide/	0.1	0.06	1	40
9/13 pm	Etoposide/	0.1	0.05	1	50
9/13 am	Etoposide/	0.1	0.02	1	80
9/13 am	Etoposide/	0.1	0.05	1	50
9/13 am	Etoposide/	0.1	0.06	1	40
9/13 am	Etoposide/	0.1	0.07	1	30
9/13 am	Etoposide/	0.1	0.08	1	20
9/13 am	Etoposide/	0.1	0.08	1	20
9/13 am	Etoposide/	0.1	0.05	1	50
9/12 am	Etoposide/	0.1	0.05	1	50
9/12 am	Etoposide/	0.1	0.06	1	40
9/12 am	Etoposide/	0.1	0.07	1	30
9/12 am	Etoposide/	0.1	0.05	1	50
9/12 am	Etoposide/	0.1	0.08	1	20
9/12 am	Etoposide/	0.1	0.08	1	20
9/12 am	Etoposide/	0.1	0.08	1	20
9/12 am	Etoposide/	0.1	0.08	1	20
9/12 am	Etoposide/	0.1	0.05	1	50
9/11 pm	Etoposide/	0.1	0.07	1	30
9/11 am	Etoposide/	0.1	0.08	1	20
9/11 am	Etoposide/	0.1	0.08	1	20

9/11 am	Etoposide/	0.1	0.08	1	20
9/11 am	Etoposide/	0.1	0.05	1	50
9/11 am	Etoposide/	0.1	0.08	1	20
9/11 am	Etoposide/	0.1	0.02	1	80
9/11 am	Etoposide/	0.1	0.06	1	40
9/11 am	Etoposide/	0.1	0.05	1	50
9/10 pm	Etoposide/	0.1	0.08	1	20
9/10 am	Etoposide/	0.1	0.08	1	20
9/10 am	Etoposide/	0.1	0.08	1	20
9/10 am	Etoposide/	0.1	0.05	1	50
9/10 am	Etoposide/	0.1	0.07	1	30
9/10 am	Etoposide/	0.1	0.06	1	40
9/10 am	Etoposide/	0.1	0.05	1	50
9/10 am	Etoposide/	0.1	0.05	1	50
10/5 pm	Etoposide/	0.1	0.03	1	70
10/2 pm	Etoposide/	0.1	0.03	1	70
10/2 am	Etoposide/	0.1	0.094	1	6
10/23 am	Etoposide/	0.1	0.03	1	70
10/23 am	Etoposide/	0.1	0.12	2	80
10/23 am	Etoposide/	0.1	0.0017	1	98.3
10/22 am	Etoposide/	0.1	0.12	2	80
10/22 am	Etoposide/	0.1	0.0017	1	98.3
10/22 am	Etoposide/	0.1	0.08	1	20
10/22 am	Etoposide/	0.1	0.076	1	24
10/1 am	Etoposide/	0.1	0.05	1	50
10/19 am	Etoposide/	0.1	0.03	1	70
10/18 am	Etoposide/	0.1	0.05	1	50
10/12 pm	Etoposide/	0.1	0.03	1	70
				239	9976.6
9/9 pm	Cytarabine	0.5	0.03	1	470
9/9 am	Cytarabine	0.5	0.048	1	452
9/9 am	Cytarabine	0.5	0.3	1	200
9/9 am	Cytarabine	0.5	0.2	1	300
9/9 am	Cytarabine	0.5	0.2	1	300
9/9 am	Cytarabine	0.5	0.369	1	131
9/8 am	Cytarabine	0.5	0.048	1	452
9/7 pm	Cytarabine	0.5	0.193	1	307
9/7 am	Cytarabine	0.5	0.193	1	307
9/6 pm	Cytarabine	0.5	0.193	1	307
9/6 pm	Cytarabine	0.5	0.2	1	300
9/6 am	Cytarabine	0.5	0.193	1	307
9/3 am	Cytarabine	0.5	0.1	1	400
9/30 pm	Cytarabine	0.5	0.085	1	415
9/30 am	Cytarabine	0.5	1.88	4	120
9/30 am	Cytarabine	0.5	0.075	1	425
9/30 am	Cytarabine	0.5	0.085	1	415
9/30 am	Cytarabine	0.5	0.2	1	300
9/30 am	Cytarabine	0.5	0.2	1	300
9/2 pm	Cytarabine	0.5	0.156	1	344
9/2 am	Cytarabine	0.5	0.1	1	400
9/29 pm	Cytarabine	0.5	0.1	1	400

9/29 pm	Cytarabine	0.5	0.2	1	300
9/29 am	Cytarabine	0.5	0.2	1	300
9/29 am	Cytarabine	0.5	0.156	1	344
9/29 am	Cytarabine	0.5	1.88	4	120
9/29 am	Cytarabine	0.5	0.085	1	415
9/28 pm	Cytarabine	0.5	0.085	1	415
9/28 pm	Cytarabine	0.5	0.085	1	415
9/28 pm	Cytarabine	0.5	1.18	4	820
9/28 am	Cytarabine	0.5	1.18	4	820
9/28 am	Cytarabine	0.5	0.075	1	425
9/28 am	Cytarabine	0.5	0.01	1	490
9/28 am	Cytarabine	0.5	0.3	1	200
9/28 am	Cytarabine	0.5	0.2	1	300
9/28 am	Cytarabine	0.5	0.2	1	300
9/28 am	Cytarabine	0.5	0.3	1	200
9/28 am	Cytarabine	0.5	0.22	1	280
9/27 pm	Cytarabine	0.5	0.075	1	425
9/27 am	Cytarabine	0.5	0.075	1	425
9/27 am	Cytarabine	0.5	0.3	1	200
9/27 am	Cytarabine	0.5	0.2	1	300
9/27 am	Cytarabine	0.5	0.2	1	300
9/27 am	Cytarabine	0.5	0.3	1	200
9/26 pm	Cytarabine	0.5	0.05	1	450
9/26 am	Cytarabine	0.5	0.075	1	425
9/26 am	Cytarabine	0.5	0.3	1	200
9/26 am	Cytarabine	0.5	0.2	1	300
9/26 am	Cytarabine	0.5	0.2	1	300
9/26 am	Cytarabine	0.5	0.3	1	200
9/25 pm	Cytarabine	0.5	0.05	1	450
9/25 pm	Cytarabine	0.5	0.2	1	300
9/25 am	Cytarabine	0.5	0.05	1	450
9/25 am	Cytarabine	0.5	0.3	1	200
9/25 am	Cytarabine	0.5	0.2	1	300
9/25 am	Cytarabine	0.5	0.3	1	200
9/25 am	Cytarabine	0.5	0.3	1	200
9/24 pm	Cytarabine	0.5	0.2	1	300
9/24 pm	Cytarabine	0.5	0.035	1	465
9/24 am	Cytarabine	0.5	0.3	1	200
9/24 am	Cytarabine	0.5	0.3	1	200
9/23 pm	Cytarabine	0.5	0.046	1	454
9/23 pm	Cytarabine	0.5	0.035	1	465
9/23 pm	Cytarabine	0.5	0.046	1	454
9/23 am	Cytarabine	0.5	0.046	1	454
9/23 am	Cytarabine	0.5	0.3	1	200
9/23 am	Cytarabine	0.5	0.2	1	300
9/23 am	Cytarabine	0.5	0.046	1	454
9/22 pm	Cytarabine	0.5	0.046	1	454
9/22 am	Cytarabine	0.5	0.046	1	454
9/21 pm	Cytarabine	0.5	0.046	1	454
9/21 am	Cytarabine	0.5	0.046	1	454
9/21 am	Cytarabine	0.5	0.3	1	200

9/21 am	Cytarabine	0.5	0.4	1	100
9/20 pm	Cytarabine	0.5	0.046	1	454
9/20 pm	Cytarabine	0.5	0.05	1	450
9/20 am	Cytarabine	0.5	0.046	1	454
9/20 am	Cytarabine	0.5	0.045	1	455
9/20 am	Cytarabine	0.5	0.3	1	200
9/20 am	Cytarabine	0.5	0.05	1	450
9/1 am	Cytarabine	0.5	0.1	1	400
9/1 am	Cytarabine	0.5	0.2	1	300
9/19 pm	Cytarabine	0.5	0.05	1	450
9/19 pm	Cytarabine	0.5	0.045	1	455
9/19 pm	Cytarabine	0.5	0.046	1	454
9/19 am	Cytarabine	0.5	0.05	1	450
9/19 am	Cytarabine	0.5	0.05	1	450
9/19 am	Cytarabine	0.5	0.045	1	455
9/19 am	Cytarabine	0.5	0.046	1	454
9/19 am	Cytarabine	0.5	0.2	1	300
9/19 am	Cytarabine	0.5	0.3	1	200
9/19 am	Cytarabine	0.5	0.2	1	300
9/18 pm	Cytarabine	0.5	0.045	1	455
9/18 pm	Cytarabine	0.5	0.05	1	450
9/18 pm	Cytarabine	0.5	0.046	1	454
9/18 am	Cytarabine	0.5	0.05	1	450
9/18 am	Cytarabine	0.5	0.046	1	454
9/18 am	Cytarabine	0.5	0.3	1	200
9/18 am	Cytarabine	0.5	0.3	1	200
9/18 am	Cytarabine	0.5	0.2	1	300
9/17 pm	Cytarabine	0.5	0.046	1	454
9/17 pm	Cytarabine	0.5	0.045	1	455
9/17 am	Cytarabine	0.5	0.046	1	454
9/17 am	Cytarabine	0.5	0.035	1	465
9/17 am	Cytarabine	0.5	0.05	1	450
9/17 am	Cytarabine	0.5	0.05	1	450
9/17 am	Cytarabine	0.5	0.2	1	300
9/17 am	Cytarabine	0.5	0.2	1	300
9/16 pm	Cytarabine	0.5	0.05	1	450
9/16 am	Cytarabine	0.5	0.05	1	450
9/16 am	Cytarabine	0.5	0.2	1	300
9/16 am	Cytarabine	0.5	0.3	1	200
9/16 am	Cytarabine	0.5	0.3	1	200
9/16 am	Cytarabine	0.5	0.2	1	300
9/16 am	Cytarabine	0.5	0.2	1	300
9/16 am	Cytarabine	0.5	0.234	1	266
9/15 pm	Cytarabine	0.5	0.05	1	450
9/15 pm	Cytarabine	0.5	0.03	1	470
9/15 pm	Cytarabine	0.5	0.3	1	200
9/15 am	Cytarabine	0.5	0.05	1	450
9/15 am	Cytarabine	0.5	0.03	1	470
9/15 am	Cytarabine	0.5	0.3	1	200
9/15 am	Cytarabine	0.5	0.2	1	300
9/15 am	Cytarabine	0.5	0.3	1	200

9/15 am	Cytarabine	0.5	0.2	1	300
9/14 pm	Cytarabine	0.5	0.05	1	450
9/14 pm	Cytarabine	0.5	0.03	1	470
9/14 pm	Cytarabine	0.5	0.3	1	200
9/14 am	Cytarabine	0.5	0.05	1	450
9/14 am	Cytarabine	0.5	0.03	1	470
9/14 am	Cytarabine	0.5	0.3	1	200
9/14 am	Cytarabine	0.5	0.2	1	300
9/14 am	Cytarabine	0.5	0.2	1	300
9/13 pm	Cytarabine	0.5	0.05	1	450
9/13 pm	Cytarabine	0.5	0.2	1	300
9/13 am	Cytarabine	0.5	0.045	1	455
9/13 am	Cytarabine	0.5	0.8	2	200
9/13 am	Cytarabine	0.5	0.27	1	230
9/13 am	Cytarabine	0.5	0.03	1	470
9/13 am	Cytarabine	0.5	0.03	1	470
9/13 am	Cytarabine	0.5	0.3	1	200
9/13 am	Cytarabine	0.5	0.2	1	300
9/13 am	Cytarabine	0.5	0.2	1	300
9/12 pm	Cytarabine	0.5	0.03	1	470
9/12 pm	Cytarabine	0.5	0.2	1	300
9/12 pm	Cytarabine	0.5	0.2	1	300
9/12 am	Cytarabine	0.5	0.8	2	200
9/12 am	Cytarabine	0.5	0.045	1	455
9/12 am	Cytarabine	0.5	0.03	1	470
9/12 am	Cytarabine	0.5	0.3	1	200
9/12 am	Cytarabine	0.5	0.2	1	300
9/11 pm	Cytarabine	0.5	0.2	1	300
9/11 pm	Cytarabine	0.5	0.2	1	300
9/11 am	Cytarabine	0.5	0.03	1	470
9/11 am	Cytarabine	0.5	0.045	1	455
9/11 am	Cytarabine	0.5	0.03	1	470
9/11 am	Cytarabine	0.5	0.3	1	200
9/11 am	Cytarabine	0.5	0.2	1	300
9/11 am	Cytarabine	0.5	0.2	1	300
9/11 am	Cytarabine	0.5	0.2	1	300
9/10 pm	Cytarabine	0.5	0.03	1	470
9/10 am	Cytarabine	0.5	0.03	1	470
9/10 am	Cytarabine	0.5	0.045	1	455
9/10 am	Cytarabine	0.5	0.3	1	200
9/10 am	Cytarabine	0.5	0.2	1	300
9/10 am	Cytarabine	0.5	0.2	1	300
9/10 am	Cytarabine	0.5	0.2	1	300
11/9 am	Cytarabine	0.5	0.2	1	300
11/8 pm	Cytarabine	0.5	0.27	1	230
11/8 am	Cytarabine	0.5	0.2	1	300
11/7 am	Cytarabine	0.5	0.2	1	300
11/6 am	Cytarabine	0.5	0.2	1	300
11/5 pm	Cytarabine	0.5	0.1	1	400
11/5 am	Cytarabine	0.5	0.2	1	300
11/5 am	Cytarabine	0.5	0.2	1	300

11/4 pm	Cytarabine	0.5	0.2	1	300
11/4 pm	Cytarabine	0.5	0.3	1	200
11/4 am	Cytarabine	0.5	0.2	1	300
11/4 am	Cytarabine	0.5	0.39	1	110
11/3 pm	Cytarabine	0.5	0.15	1	350
11/3 pm	Cytarabine	0.5	0.2	1	300
11/3 am	Cytarabine	0.5	0.15	1	350
11/3 am	Cytarabine	0.5	0.2	1	300
11/30 am	Cytarabine	0.5	0.15	1	350
11/30 am	Cytarabine	0.5	0.2	1	300
11/2 pm	Cytarabine	0.5	0.15	1	350
11/2 am	Cytarabine	0.5	0.15	1	350
11/2 am	Cytarabine	0.5	0.2	1	300
11/29 am	Cytarabine	0.5	0.1	1	400
11/29 am	Cytarabine	0.5	0.15	1	350
11/29 am	Cytarabine	0.5	0.2	1	300
11/29 am	Cytarabine	0.5	0.255	1	245
11/29 am	Cytarabine	0.5	0.24	1	260
11/28 am	Cytarabine	0.5	0.1	1	400
11/28 am	Cytarabine	0.5	0.15	1	350
11/28 am	Cytarabine	0.5	0.2	1	300
11/27 am	Cytarabine	0.5	0.1	1	400
11/27 am	Cytarabine	0.5	0.15	1	350
11/27 am	Cytarabine	0.5	0.2	1	300
11/27 am	Cytarabine	0.5	0.2	1	300
11/26 pm	Cytarabine	0.5	0.1	1	400
11/26 am	Cytarabine	0.5	0.2	1	300
11/26 am	Cytarabine	0.5	0.22	1	280
11/26 am	Cytarabine	0.5	1.8	4	200
11/26 am	Cytarabine	0.5	1.8	4	200
11/25 am	Cytarabine	0.5	0.2	1	300
11/25 am	Cytarabine	0.5	0.255	1	245
11/25 am	Cytarabine	0.5	0.035	1	465
11/25 am	Cytarabine	0.5	0.165	1	335
11/25 am	Cytarabine	0.5	1.8	4	200
11/25 am	Cytarabine	0.5	1.8	4	200
11/24 am	Cytarabine	0.5	0.07	1	430
11/24 am	Cytarabine	0.5	0.2	1	300
11/2311/23	Cytarabine	0.5	0.07	1	430
11/2311/23	Cytarabine	0.5	0.2	1	300
11/2311/23	Cytarabine	0.5	0.035	1	465
11/2311/23	Cytarabine	0.5	1.8	4	200
11/22 am	Cytarabine	0.5	0.07	1	430
11/22 am	Cytarabine	0.5	0.2	1	300
11/22 am	Cytarabine	0.5	0.3	1	200
11/21 am	Cytarabine	0.5	0.07	1	430
11/21 am	Cytarabine	0.5	0.2	1	300
11/20 am	Cytarabine	0.5	0.2	1	300
11/20 am	Cytarabine	0.5	1.8	4	200
11/1 pm	Cytarabine	0.5	0.15	1	350
11/1 pm	Cytarabine	0.5	0.14	1	360

11/1 am	Cytarabine	0.5	0.15	1	350
11/1 am	Cytarabine	0.5	0.2	1	300
11/17 pm	Cytarabine	0.5	1.8	4	200
11/17 pm	Cytarabine	0.5	1.8	4	200
11/17 am	Cytarabine	0.5	0.07	1	430
11/16 am	Cytarabine	0.5	0.2	1	300
11/15 am	Cytarabine	0.5	0.07	1	430
11/15 am	Cytarabine	0.5	0.2	1	300
11/14 am	Cytarabine	0.5	0.07	1	430
11/14 am	Cytarabine	0.5	0.2	1	300
11/13 am	Cytarabine	0.5	0.2	1	300
11/12 am	Cytarabine	0.5	0.2	1	300
11/11 am	Cytarabine	0.5	0.2	1	300
11/10 am	Cytarabine	0.5	0.2	1	300
10/9 pm	Cytarabine	0.5	0.03	1	470
10/9 am	Cytarabine	0.5	0.03	1	470
10/8 pm	Cytarabine	0.5	0.03	1	470
10/8 am	Cytarabine	0.5	0.03	1	470
10/8 am	Cytarabine	0.5	0.2	1	300
10/7 pm	Cytarabine	0.5	0.369	1	131
10/7 am	Cytarabine	0.5	0.2	1	300
10/7 am	Cytarabine	0.5	0.2	1	300
10/6 am	Cytarabine	0.5	0.2	1	300
10/6 am	Cytarabine	0.5	0.2	1	300
10/6 am	Cytarabine	0.5	0.2	1	300
10/5 pm	Cytarabine	0.5	0.15	1	350
10/5 pm	Cytarabine	0.5	0.035	1	465
10/5 am	Cytarabine	0.5	0.15	1	350
10/5 am	Cytarabine	0.5	0.2	1	300
10/5 am	Cytarabine	0.5	0.2	1	300
10/5 am	Cytarabine	0.5	0.2	1	300
10/4 pm	Cytarabine	0.5	0.15	1	350
10/4 am	Cytarabine	0.5	0.15	1	350
10/4 am	Cytarabine	0.5	0.2	1	300
10/4 am	Cytarabine	0.5	0.2	1	300
10/4 am	Cytarabine	0.5	0.2	1	300
10/3 pm	Cytarabine	0.5	0.15	1	350
10/3 pm	Cytarabine	0.5	0.035	1	465
10/3 am	Cytarabine	0.5	0.15	1	350
10/3 am	Cytarabine	0.5	0.075	1	425
10/3 am	Cytarabine	0.5	0.2	1	300
10/3 am	Cytarabine	0.5	0.2	1	300
10/3 am	Cytarabine	0.5	0.2	1	300
10/3 am	Cytarabine	0.5	0.15	1	350
10/31 am	Cytarabine	0.5	0.15	1	350
10/31 am	Cytarabine	0.5	0.15	1	350
10/31 am	Cytarabine	0.5	0.2	1	300
10/30 pm	Cytarabine	0.5	0.15	1	350
10/30 am	Cytarabine	0.5	0.15	1	350
10/30 am	Cytarabine	0.5	0.2	1	300
10/2 am	Cytarabine	0.5	0.15	1	350

10/2 am	Cytarabine	0.5	0.15	1	350
10/2 am	Cytarabine	0.5	0.075	1	425
10/2 am	Cytarabine	0.5	0.2	1	300
10/2 am	Cytarabine	0.5	0.2	1	300
10/2 am	Cytarabine	0.5	0.2	1	300
10/29 pm	Cytarabine	0.5	0.15	1	350
10/29 pm	Cytarabine	0.5	0.035	1	465
10/29 pm	Cytarabine	0.5	0.035	1	465
10/29 am	Cytarabine	0.5	0.15	1	350
10/29 am	Cytarabine	0.5	0.2	1	300
10/28 am	Cytarabine	0.5	0.1	1	400
10/28 am	Cytarabine	0.5	0.15	1	350
10/28 am	Cytarabine	0.5	0.15	1	350
10/28 am	Cytarabine	0.5	0.2	1	300
10/28 am	Cytarabine	0.5	0.15	1	350
10/27 am	Cytarabine	0.5	0.1	1	400
10/27 am	Cytarabine	0.5	0.15	1	350
10/27 am	Cytarabine	0.5	0.2	1	300
10/27 am	Cytarabine	0.5	0.2	1	300
10/26 am	Cytarabine	0.5	0.1	1	400
10/26 am	Cytarabine	0.5	0.15	1	350
10/26 am	Cytarabine	0.5	0.2	1	300
10/26 am	Cytarabine	0.5	0.2	1	300
10/25 am	Cytarabine	0.5	0.15	1	350
10/25 am	Cytarabine	0.5	0.2	1	300
10/25 am	Cytarabine	0.5	0.2	1	300
10/24 pm	Cytarabine	0.5	0.047	1	453
10/24 am	Cytarabine	0.5	0.047	1	453
10/24 am	Cytarabine	0.5	0.15	1	350
10/24 am	Cytarabine	0.5	0.2	1	300
10/23 pm	Cytarabine	0.5	0.047	1	453
10/23 am	Cytarabine	0.5	0.047	1	453
10/23 am	Cytarabine	0.5	0.2	1	300
10/22 pm	Cytarabine	0.5	0.047	1	453
10/22 am	Cytarabine	0.5	0.047	1	453
10/22 am	Cytarabine	0.5	0.2	1	300
10/21 pm	Cytarabine	0.5	0.047	1	453
10/21 am	Cytarabine	0.5	0.047	1	453
10/21 am	Cytarabine	0.5	0.06	1	440
10/21 am	Cytarabine	0.5	0.048	1	452
10/21 am	Cytarabine	0.5	0.2	1	300
10/20 pm	Cytarabine	0.5	0.047	1	453
10/20 am	Cytarabine	0.5	0.047	1	453
10/20 am	Cytarabine	0.5	0.06	1	440
10/20 am	Cytarabine	0.5	0.048	1	452
10/20 am	Cytarabine	0.5	0.2	1	300
10/1 pm	Cytarabine	0.5	0.15	1	350
10/1 am	Cytarabine	0.5	0.035	1	465
10/1 am	Cytarabine	0.5	0.15	1	350
10/1 am	Cytarabine	0.5	0.075	1	425
10/1 am	Cytarabine	0.5	0.2	1	300

10/1 am	Cytarabine	0.5	0.2	1	300
10/1 am	Cytarabine	0.5	0.2	1	300
10/19 am	Cytarabine	0.5	0.047	1	453
10/19 am	Cytarabine	0.5	0.06	1	440
10/19 am	Cytarabine	0.5	0.048	1	452
10/19 am	Cytarabine	0.5	0.047	1	453
10/19 am	Cytarabine	0.5	0.2	1	300
10/18 pm	Cytarabine	0.5	0.047	1	453
10/18 am	Cytarabine	0.5	0.2	1	300
10/18 am	Cytarabine	0.5	0.047	1	453
10/18 am	Cytarabine	0.5	0.06	1	440
10/18 am	Cytarabine	0.5	0.048	1	452
10/18 am	Cytarabine	0.5	0.2	1	300
10/16 am	Cytarabine	0.5	0.03	1	470
10/16 am	Cytarabine	0.5	0.3	3	1200
10/16 am	Cytarabine	0.5	0.2	1	300
10/16 am	Cytarabine	0.5	0.2	1	300
10/15 am	Cytarabine	0.5	0.075	1	425
10/15 am	Cytarabine	0.5	0.3	3	1200
10/15 am	Cytarabine	0.5	0.2	1	300
10/15 am	Cytarabine	0.5	0.2	1	300
10/14 pm	Cytarabine	0.5	0.03	1	470
10/14 am	Cytarabine	0.5	0.03	1	470
10/14 am	Cytarabine	0.5	0.06	1	440
10/14 am	Cytarabine	0.5	0.048	1	452
10/14 am	Cytarabine	0.5	0.075	1	425
10/14 am	Cytarabine	0.5	0.3	1	200
10/14 am	Cytarabine	0.5	0.2	1	300
10/14 am	Cytarabine	0.5	0.2	1	300
10/13 pm	Cytarabine	0.5	0.03	1	470
10/13 pm	Cytarabine	0.5	0.3	1	200
10/13 am	Cytarabine	0.5	0.03	1	470
10/13 am	Cytarabine	0.5	0.06	1	440
10/13 am	Cytarabine	0.5	0.048	1	452
10/13 am	Cytarabine	0.5	0.075	1	425
10/12 pm	Cytarabine	0.5	0.03	1	470
10/12 am	Cytarabine	0.5	0.03	1	470
10/12 am	Cytarabine	0.5	0.06	1	440
10/12 am	Cytarabine	0.5	0.048	1	452
10/12 am	Cytarabine	0.5	0.075	1	425
10/12 am	Cytarabine	0.5	0.1	1	400
10/11 pm	Cytarabine	0.5	0.03	1	470
10/11 pm	Cytarabine	0.5	0.3	1	200
10/11 am	Cytarabine	0.5	0.03	1	470
10/11 am	Cytarabine	0.5	0.048	1	452
10/11 am	Cytarabine	0.5	0.06	1	440
10/10 pm	Cytarabine	0.5	0.03	1	470
10/10 am	Cytarabine	0.5	0.03	1	470
				418	134075
9/25 am	Oxaliplatin	50	136	3	14
9/23 am	Oxaliplatin	50	240	5	10

9/23 am	Oxaliplatir	50	190	4	10
9/17 am	Oxaliplatir	50	220	5	30
10/6 am	Oxaliplatir	50	188	4	12
10/1 am	Oxaliplatir	50	120	3	30
				24	106
9/13 pm	Actinomyc	0.2	0.5	3	0.1
11/8 am	Actinomyc	0.2	0.45	3	0.15
11/13 pm	Actinomyc	0.2	0.5	3	0.1
10/30 pm	Actinomyc	0.2	0.5	3	0.1
10/29 am	Actinomyc	0.2	0.5	3	0.1
10/13 pm	Actinomyc	0.2	0.45	3	0.15
				18	0.7
9/9 pm	Cyclophos	0.2	0.59	3	10
9/9 am	Cyclophos	0.2	0.9	5	100
9/9 am	Cyclophos	0.2	0.369	2	31
9/9 am	Cyclophos	0.2	0.7	4	100
9/8 am	Cyclophos	0.2	0.925	5	75
9/8 am	Cyclophos	0.2	0.59	3	10
9/7 pm	Cyclophos	0.2	0.5	3	100
9/7 am	Cyclophos	0.2	0.9	5	100
9/7 am	Cyclophos	0.2	1.1	6	100
9/7 am	Cyclophos	0.2	0.9	5	100
9/7 am	Cyclophos	0.2	0.9	5	100
9/6 am	Cyclophos	0.2	0.9	5	100
9/5 am	Cyclophos	0.2	0.9	5	100
9/3 pm	Cyclophos	0.2	0.3	2	100
9/3 pm	Cyclophos	0.2	1.1	6	100
9/30 pm	Cyclophos	0.2	0.9	5	100
9/30 am	Cyclophos	0.2	0.254	2	146
9/30 am	Cyclophos	0.2	0.9	5	100
9/2 pm	Cyclophos	0.2	0.156	1	44
9/2 am	Cyclophos	0.2	0.9	5	100
9/2 am	Cyclophos	0.2	0.5	3	100
9/29 am	Cyclophos	0.2	0.254	2	146
9/29 am	Cyclophos	0.2	0.97	5	30
9/29 am	Cyclophos	0.2	0.156	1	44
9/29 am	Cyclophos	0.2	0.3	2	100
9/29 am	Cyclophos	0.2	0.9	5	100
9/28 pm	Cyclophos	0.2	0.254	2	146
9/28 pm	Cyclophos	0.2	0.22	2	180
9/27 pm	Cyclophos	0.2	0.3	2	100
9/27 am	Cyclophos	0.2	1.3	7	100
9/25 pm	Cyclophos	0.2	0.3	2	100
9/25 am	Cyclophos	0.2	1.1	6	100
9/24 pm	Cyclophos	0.2	0.9	5	100
9/24 pm	Cyclophos	0.2	0.5	3	100
9/24 pm	Cyclophos	0.2	0.5	3	100
9/24 am	Cyclophos	0.2	1.1	6	100
9/24 am	Cyclophos	0.2	0.9	5	100
9/23 am	Cyclophos	0.2	1.1	6	100
9/23 am	Cyclophos	0.2	0.3	2	100

9/23 am	Cyclophos	0.2	0.9	5	100
9/22 am	Cyclophos	0.2	0.9	5	100
9/21 am	Cyclophos	0.2	0.5	3	100
9/21 am	Cyclophos	0.2	0.5	3	100
9/20 pm	Cyclophos	0.2	0.5	3	100
9/1 pm	Cyclophos	0.2	0.7	4	100
9/1 am	Cyclophos	0.2	0.9	5	100
9/1 am	Cyclophos	0.2	0.75	4	50
9/19 pm	Cyclophos	0.2	0.5	3	100
9/18 pm	Cyclophos	0.2	0.7	4	100
9/18 am	Cyclophos	0.2	0.12	1	80
9/18 am	Cyclophos	0.2	1.1	6	100
9/17 pm	Cyclophos	0.2	0.9	5	100
9/17 am	Cyclophos	0.2	0.12	1	80
9/17 am	Cyclophos	0.2	0.3	2	100
9/17 am	Cyclophos	0.2	0.3	2	100
9/16 am	Cyclophos	0.2	0.85	5	150
9/16 am	Cyclophos	0.2	0.95	5	50
9/16 am	Cyclophos	0.2	0.12	1	80
9/16 am	Cyclophos	0.2	0.7	4	100
9/16 am	Cyclophos	0.2	0.7	4	100
9/16 am	Cyclophos	0.2	1.1	6	100
9/15 pm	Cyclophos	0.2	0.7	4	100
9/15 am	Cyclophos	0.2	0.12	2	280
9/15 am	Cyclophos	0.2	1.1	6	100
9/15 am	Cyclophos	0.2	1.3	7	100
9/14 am	Cyclophos	0.2	0.12	2	280
9/14 am	Cyclophos	0.2	1.1	6	100
9/14 am	Cyclophos	0.2	0.95	5	50
9/14 am	Cyclophos	0.2	0.95	5	50
9/14 am	Cyclophos	0.2	0.9	5	100
9/14 am	Cyclophos	0.2	0.9	5	100
9/14 am	Cyclophos	0.2	0.5	3	100
9/14 am	Cyclophos	0.2	0.7	8	900
9/13 am	Cyclophos	0.2	0.27	2	130
9/11 pm	Cyclophos	0.2	0.7	4	100
9/10 am	Cyclophos	0.2	0.3	2	100
9/10 am	Cyclophos	0.2	0.97	5	30
11/9 pm	Cyclophos	0.2	0.88	5	120
11/9 pm	Cyclophos	0.2	0.97	5	30
11/9 am	Cyclophos	0.2	0.9	5	100
11/9 am	Cyclophos	0.2	0.95	5	50
11/8 pm	Cyclophos	0.2	0.7	4	100
11/8 am	Cyclophos	0.2	0.27	2	130
11/6 am	Cyclophos	0.2	0.7	4	100
11/5 pm	Cyclophos	0.2	3.3	17	100
11/4 pm	Cyclophos	0.2	0.7	4	100
11/4 am	Cyclophos	0.2	0.3	2	100
11/4 am	Cyclophos	0.2	0.3	2	100
11/4 am	Cyclophos	0.2	0.3	2	100
11/4 am	Cyclophos	0.2	0.39	2	10

11/3 pm	Cyclophos	0.2	0.3	2	100
11/3 am	Cyclophos	0.2	0.7	4	100
11/3 am	Cyclophos	0.2	1.1	6	100
11/30 am	Cyclophos	0.2	0.9	5	100
11/30 am	Cyclophos	0.2	0.9	5	100
11/2 pm	Cyclophos	0.2	0.875	5	125
11/2 am	Cyclophos	0.2	0.9	5	100
11/29 am	Cyclophos	0.2	0.24	2	160
11/28 pm	Cyclophos	0.2	0.7	4	100
11/28 am	Cyclophos	0.2	0.5	3	100
11/28 am	Cyclophos	0.2	0.7	4	100
11/27 pm	Cyclophos	0.2	0.5	3	100
11/27 am	Cyclophos	0.2	0.7	4	100
11/27 am	Cyclophos	0.2	1.1	6	100
11/26 pm	Cyclophos	0.2	0.9	5	100
11/25 am	Cyclophos	0.2	0.165	1	35
11/24 pm	Cyclophos	0.2	0.9	5	100
11/24 pm	Cyclophos	0.2	0.3	2	100
11/23 pm	Cyclophos	0.2	0.5	3	100
11/23 am	Cyclophos	0.2	0.98	5	20
11/23 am	Cyclophos	0.2	0.9	5	100
11/23 am	Cyclophos	0.2	0.85	5	150
11/23 am	Cyclophos	0.2	0.9	5	100
11/22 am	Cyclophos	0.2	0.3	2	100
11/21 pm	Cyclophos	0.2	0.5	3	100
11/21 am	Cyclophos	0.2	0.9	5	100
11/21 am	Cyclophos	0.2	0.3	2	100
11/20 am	Cyclophos	0.2	0.3	2	100
11/20 am	Cyclophos	0.2	0.3	2	100
11/1 pm	Cyclophos	0.2	0.14	1	60
11/1 am	Cyclophos	0.2	1.1	6	100
11/19 am	Cyclophos	0.2	0.3	2	100
11/19 am	Cyclophos	0.2	0.3	2	100
11/19 am	Cyclophos	0.2	0.9	5	100
11/19 am	Cyclophos	0.2	0.5	3	100
11/19 am	Cyclophos	0.2	0.5	3	100
11/18 am	Cyclophos	0.2	0.67	4	130
11/18 am	Cyclophos	0.2	1.1	6	100
11/17 am	Cyclophos	0.2	0.7	4	100
11/17 am	Cyclophos	0.2	0.7	4	100
11/17 am	Cyclophos	0.2	0.45	3	150
11/16 pm	Cyclophos	0.2	0.225	2	175
11/16 pm	Cyclophos	0.2	0.5	3	100
11/16 am	Cyclophos	0.2	0.07	1	130
11/16 am	Cyclophos	0.2	0.9	5	100
11/16 am	Cyclophos	0.2	0.7	4	100
11/13 am	Cyclophos	0.2	0.95	5	50
11/12 pm	Cyclophos	0.2	0.3	2	100
11/12 am	Cyclophos	0.2	0.94	5	60
11/12 am	Cyclophos	0.2	0.5	3	100
11/12 am	Cyclophos	0.2	0.97	5	30

11/11 pm	Cyclophos	0.2	0.9	5	100
11/11 pm	Cyclophos	0.2	1.3	7	100
11/11 am	Cyclophos	0.2	0.9	5	100
10/8 pm	Cyclophos	0.2	0.3	2	100
10/8 am	Cyclophos	0.2	1.1	6	100
10/7 pm	Cyclophos	0.2	0.369	2	31
10/7 am	Cyclophos	0.2	0.3	2	100
10/6 am	Cyclophos	0.2	0.3	2	100
10/2 am	Cyclophos	0.2	0.254	2	146
10/29 pm	Cyclophos	0.2	0.7	4	100
10/28 pm	Cyclophos	0.2	0.15	1	50
10/28 am	Cyclophos	0.2	0.9	5	100
10/27 pm	Cyclophos	0.2	0.5	3	100
10/27 am	Cyclophos	0.2	0.3	2	100
10/26 am	Cyclophos	0.2	0.9	5	100
10/26 am	Cyclophos	0.2	0.9	5	100
10/26 am	Cyclophos	0.2	0.9	5	100
10/26 am	Cyclophos	0.2	0.3	2	100
10/22 pm	Cyclophos	0.2	0.3	2	100
10/22 am	Cyclophos	0.2	0.36	2	40
10/22 am	Cyclophos	0.2	0.5	3	100
10/22 am	Cyclophos	0.2	0.3	2	100
10/22 am	Cyclophos	0.2	0.7	4	100
10/22 am	Cyclophos	0.2	0.97	5	30
10/21 am	Cyclophos	0.2	0.3	2	100
10/21 am	Cyclophos	0.2	0.3	2	100
10/21 am	Cyclophos	0.2	0.3	2	100
10/21 am	Cyclophos	0.2	0.3	2	100
10/21 am	Cyclophos	0.2	0.36	2	40
10/21 am	Cyclophos	0.2	0.9	5	100
10/21 am	Cyclophos	0.2	0.7	4	100
10/20 am	Cyclophos	0.2	0.3	2	100
10/1 am	Cyclophos	0.2	0.254	2	146
10/1 am	Cyclophos	0.2	1.1	6	100
10/18 am	Cyclophos	0.2	0.98	5	20
10/18 am	Cyclophos	0.2	0.9	5	100
10/18 am	Cyclophos	0.2	0.7	4	100
10/18 am	Cyclophos	0.2	0.87	5	130
10/16 am	Cyclophos	0.2	0.51	3	90
10/16 am	Cyclophos	0.2	0.9	5	100
10/15 pm	Cyclophos	0.2	0.5	3	100
10/15 pm	Cyclophos	0.2	0.3	2	100
10/15 pm	Cyclophos	0.2	0.3	2	100
10/15 am	Cyclophos	0.2	0.51	3	90
10/15 am	Cyclophos	0.2	0.9	5	100
10/14 pm	Cyclophos	0.2	0.51	3	90
10/13 pm	Cyclophos	0.2	0.9	5	100
10/13 am	Cyclophos	0.2	0.3	2	100
10/12 pm	Cyclophos	0.2	0.7	4	100
10/12 am	Cyclophos	0.2	0.7	4	100
10/11 pm	Cyclophos	0.2	0.7	4	100

10/11 am	Cyclophos	0.2	0.7	4	100
10/10 am	Cyclophos	0.2	0.7	4	100
				734	19590
11/5 am	Ratitrexed	2	3.2	2	0.8
11/29 am	Ratitrexed	2	3.8	2	0.2
11/27 pm	Ratitrexed	2	4.8	3	1.2
10/30 am	Ratitrexed	2	5	3	1
				10	3.2
9/9 am	Vincristine	1	1.12	2	0.88
9/9 am	Vincristine	1	1.04	2	0.96
9/9 am	Vincristine	1	1.5	2	0.5
9/8 am	Vincristine	1	1.5	2	0.5
9/7 am	Vincristine	1	1.5	2	0.5
9/6 am	Vincristine	1	1.45	2	0.55
9/6 am	Vincristine	1	0.85	1	0.15
9/5 am	Vincristine	1	1.35	2	0.65
9/4 am	Vincristine	1	0.8	1	0.2
9/3 am	Vincristine	1	0.67	1	0.33
9/2 pm	Vincristine	1	1.12	2	0.88
9/2 pm	Vincristine	1	0.78	1	0.22
9/2 am	Vincristine	1	1.75	2	0.25
9/29 am	Vincristine	1	0.78	1	0.22
9/28 am	Vincristine	1	1.1	2	0.9
9/27 am	Vincristine	1	0.85	1	0.15
9/25 am	Vincristine	1	1.5	2	0.5
9/24 am	Vincristine	1	0.88	1	0.12
9/24 am	Vincristine	1	1.2	2	0.8
9/23 am	Vincristine	1	1.35	2	0.65
9/23 am	Vincristine	1	1.25	2	0.75
9/23 am	Vincristine	1	1.6	2	0.4
9/22 pm	Vincristine	1	0.67	1	0.33
9/20 am	Vincristine	1	0.45	1	0.55
9/20 am	Vincristine	1	1.57	2	0.43
9/1 am	Vincristine	1	1.5	2	0.5
9/19 am	Vincristine	1	0.45	1	0.55
9/17 am	Vincristine	1	1.2	2	0.8
9/17 am	Vincristine	1	1.1	2	0.9
9/17 am	Vincristine	1	0.88	1	0.12
9/17 am	Vincristine	1	1.57	2	0.43
9/16 am	Vincristine	1	1.2	2	0.8
9/16 am	Vincristine	1	1.5	2	0.5
9/16 am	Vincristine	1	1.69	2	0.31
9/15 am	Vincristine	1	1.12	2	0.88
9/15 am	Vincristine	1	0.67	1	0.33
9/13 pm	Vincristine	1	1.39	2	0.61
9/13 am	Vincristine	1	0.85	1	0.15
9/13 am	Vincristine	1	1.3	2	0.7
9/11 am	Vincristine	1	1.2	2	0.8
9/10 am	Vincristine	1	1.845	2	0.155
11/8 am	Vincristine	1	0.67	1	0.33
11/8 am	Vincristine	1	1.35	2	0.65

11/8 am	Vincristine	1	1.3	2	0.7
11/5 am	Vincristine	1	1.35	2	0.65
11/5 am	Vincristine	1	1.95	2	0.05
11/27 am	Vincristine	1	1.6	2	0.4
11/26 am	Vincristine	1	1.35	2	0.65
11/25 am	Vincristine	1	0.8	1	0.2
11/24 am	Vincristine	1	1.3	2	0.7
11/22 am	Vincristine	1	1.5	2	0.5
11/20 am	Vincristine	1	1.35	2	0.65
11/19 am	Vincristine	1	1.35	2	0.65
11/19 am	Vincristine	1	1.2	2	0.8
11/18 am	Vincristine	1	1.25	2	0.75
11/18 am	Vincristine	1	1.2	2	0.8
11/16 am	Vincristine	1	0.9	1	0.1
11/14 am	Vincristine	1	1.17	2	0.83
11/12 am	Vincristine	1	0.67	1	0.33
11/12 am	Vincristine	1	1.67	2	0.33
11/11 am	Vincristine	1	1.5	2	0.5
11/11 am	Vincristine	1	1.69	2	0.31
10/8 am	Vincristine	1	1.845	2	0.155
10/7 pm	Vincristine	1	1.04	2	0.96
10/6 pm	Vincristine	1	1.41	2	0.59
10/5 am	Vincristine	1	1.6	2	0.4
10/5 am	Vincristine	1	0.85	1	0.15
10/28 am	Vincristine	1	0.75	1	0.25
10/27 pm	Vincristine	1	1.41	2	0.59
10/26 am	Vincristine	1	1.8	2	0.2
10/25 pm	Vincristine	1	0.9	1	0.1
10/23 am	Vincristine	1	0.88	1	0.12
10/22 am	Vincristine	1	1.28	2	0.72
10/21 am	Vincristine	1	1.2	2	0.8
10/21 am	Vincristine	1	1.3	2	0.7
10/19 pm	Vincristine	1	0.78	1	0.22
10/19 am	Vincristine	1	0.78	1	0.22
10/16 am	Vincristine	1	1.2	2	0.8
10/16 am	Vincristine	1	0.88	1	0.12
10/15 am	Vincristine	1	1.57	2	0.43
10/14 am	Vincristine	1	1.69	2	0.31
10/14 am	Vincristine	1	1.8	2	0.2
10/13 am	Vincristine	1	1.41	2	0.59
10/13 am	Vincristine	1	1.2	2	0.8
10/13 am	Vincristine	1	0.67	1	0.33
10/11 pm	Vincristine	1	1.5	2	0.5
				147	42.04
9/9 am	Loplatin	10	45	5	5
9/4 am	Loplatin	10	5	1	5
9/1 am	Loplatin	10	35	4	5
9/17 am	Loplatin	10	45	5	5
9/16 am	Loplatin	10	45	5	5
9/11 am	Loplatin	10	45	5	5
9/11 am	Loplatin	10	45	5	5

10/27 am	Loplatin	10	78	8	2
10/23 am	Loplatin	10	5	1	5
				39	42
9/7 am	Loplatin	50	45	1	5
9/4 am	Loplatin	50	55	2	45
9/4 am	Loplatin	50	75	2	25
9/2 am	Loplatin	50	40	1	10
9/27 am	Loplatin	50	40	1	10
9/27 am	Loplatin	50	40	1	10
9/27 am	Loplatin	50	40	1	10
9/24 am	Loplatin	50	45	1	5
9/23 am	Loplatin	50	40	1	10
9/14 am	Loplatin	50	45	1	5
9/14 am	Loplatin	50	40	1	10
9/11 am	Loplatin	50	45	1	5
11/9 am	Loplatin	50	45	1	5
11/8 pm	Loplatin	50	45	1	5
11/6 am	Loplatin	50	40	1	10
11/3 am	Loplatin	50	45	1	5
11/25 am	Loplatin	50	40	1	10
11/11 pm	Loplatin	50	40	1	10
11/11 am	Loplatin	50	45	1	5
11/10 am	Loplatin	50	40	1	10
10/6 pm	Loplatin	50	40	1	10
10/3 am	Loplatin	50	40	1	10
10/31 pm	Loplatin	50	40	1	10
10/30 am	Loplatin	50	40	1	10
10/28 am	Loplatin	50	45	1	5
10/23 am	Loplatin	50	40	1	10
10/23 am	Loplatin	50	40	1	10
10/23 am	Loplatin	50	40	1	10
10/23 am	Loplatin	50	40	1	10
10/23 am	Loplatin	50	45	1	5
10/1 am	Loplatin	50	45	1	5
10/19 am	Loplatin	50	45	1	5
10/12 am	Loplatin	50	45	1	5
				35	315
9/9 am	Nedaplatin	10	147	15	3
11/10 am	Nedaplatin	10	136	14	4
10/16 am	Nedaplatin	10	123	13	7
10/11 am	Nedaplatin	10	127	13	3
				55	17
9/7 am	Nedaplatin	50	140	3	10
9/3 am	Nedaplatin	50	140	3	10
				6	20
9/7 am	Pemetrexe	100	280	3	20
9/29 am	Pemetrexe	100	760	8	40
9/23 am	Pemetrexe	100	280	3	20
9/20 am	Pemetrexe	100	785	8	15
9/1 am	Pemetrexe	100	280	3	20
9/1 am	Pemetrexe	100	760	8	40

9/19 am	Pemetrexe	100	650	7	50
9/18 am	Pemetrexe	100	230	3	70
9/18 am	Pemetrexe	100	250	3	50
9/17 am	Pemetrexe	100	850	9	50
9/17 am	Pemetrexe	100	350	4	50
9/17 am	Pemetrexe	100	790	8	10
9/16 am	Pemetrexe	100	415	5	85
9/16 am	Pemetrexe	100	915	10	85
9/14 am	Pemetrexe	100	750	8	50
9/14 am	Pemetrexe	100	770	8	30
9/13 am	Pemetrexe	100	840	9	60
9/12 am	Pemetrexe	100	850	9	50
9/10 am	Pemetrexe	100	850	9	50
11/9 am	Pemetrexe	100	280	3	20
11/9 am	Pemetrexe	100	850	9	50
11/9 am	Pemetrexe	100	260	3	40
11/9 am	Pemetrexe	100	260	3	40
11/5 am	Pemetrexe	100	880	9	20
11/5 am	Pemetrexe	100	850	9	50
11/3 am	Pemetrexe	100	770	8	30
11/30 am	Pemetrexe	100	780	8	20
11/30 am	Pemetrexe	100	350	4	50
11/2 am	Pemetrexe	100	770	8	30
11/2 am	Pemetrexe	100	850	9	50
11/29 am	Pemetrexe	100	850	9	50
11/27 am	Pemetrexe	100	840	9	60
11/26 am	Pemetrexe	100	880	9	20
11/25 am	Pemetrexe	100	850	9	50
11/25 am	Pemetrexe	100	790	8	10
11/25 am	Pemetrexe	100	780	8	20
11/20 am	Pemetrexe	100	150	2	50
11/1 am	Pemetrexe	100	650	7	50
11/1 am	Pemetrexe	100	350	4	50
11/19 am	Pemetrexe	100	850	9	50
11/17 am	Pemetrexe	100	885	9	15
11/16 am	Pemetrexe	100	785	8	15
11/12 am	Pemetrexe	100	780	8	20
10/9 am	Pemetrexe	100	890	9	10
10/8 am	Pemetrexe	100	380	4	20
10/28 am	Pemetrexe	100	850	9	50
10/27 am	Pemetrexe	100	250	3	50
10/23 am	Pemetrexe	100	750	8	50
10/21 am	Pemetrexe	100	290	3	10
10/1 am	Pemetrexe	100	740	8	60
10/1 am	Pemetrexe	100	250	3	50
10/15 am	Pemetrexe	100	880	9	20
10/14 am	Pemetrexe	100	850	9	50
10/14 am	Pemetrexe	100	250	3	50
10/13 am	Pemetrexe	100	840	9	60
10/12 am	Pemetrexe	100	770	8	30
10/12 am	Pemetrexe	100	850	9	50

10/11 am	Pemetrexe	100	850	9	50
				400	2315
9/8 am	Pemetrexe	500	900	2	100
9/14 am	Pemetrexe	500	750	2	250
				4	350
11/22 am	Bortezomi	1	1.8	2	0.2
10/7 am	Bortezomi	1	2.3	3	0.7
10/25 am	Bortezomi	1	1.8	2	0.2
10/21 pm	Bortezomi	1	1.8	2	0.2
10/20 am	Bortezomi	1	1.9	2	0.1
10/18 pm	Bortezomi	1	2.3	3	0.7
10/16 pm	Bortezomi	1	1.8	2	0.2
				16	2.3
9/23 am	Trastuzum	440	360	1	80
10/13 am	Trastuzum	440	360	1	80
				2	160
9/8 am	Epirubicin /Manufactu	10	138	14	2
				14	2
9/7 pm	Epirubicin	10	65	7	5
9/5 am	Epirubicin	10	55	6	5
9/5 am	Epirubicin	10	55	6	5
9/4 am	Epirubicin	10	55	6	5
9/4 am	Epirubicin	10	55	6	5
9/2 am	Epirubicin	10	45	5	5
9/25 am	Epirubicin	10	65	7	5
9/24 am	Epirubicin	10	65	7	5
9/23 am	Epirubicin	10	45	5	5
9/21 am	Epirubicin	10	55	6	5
9/20 am	Epirubicin	10	45	5	5
9/20 am	Epirubicin	10	55	6	5
9/1 am	Epirubicin	10	45	5	5
9/19 am	Epirubicin	10	45	5	5
11/9 pm	Epirubicin	10	65	7	5
11/7 am	Epirubicin	10	65	7	5
11/6 am	Epirubicin	10	65	7	5
11/3 am	Epirubicin	10	65	7	5
11/2 am	Epirubicin	10	65	7	5
11/2 am	Epirubicin	10	55	6	5
11/28 am	Epirubicin	10	45	5	5
11/27 am	Epirubicin	10	65	7	5
11/27 am	Epirubicin	10	45	5	5
11/26 am	Epirubicin	10	65	7	5
11/1 am	Epirubicin	10	55	6	5
11/18 am	Epirubicin	10	65	7	5
11/17 am	Epirubicin	10	65	7	5
11/16 am	Epirubicin	10	55	6	5
11/16 am	Epirubicin	10	55	6	5
11/15 am	Epirubicin	10	55	6	5
11/11 am	Epirubicin	10	65	7	5
11/10 pm	Epirubicin	10	65	7	5

10/7 am	Epirubicin	10	45	5	5
10/7 am	Epirubicin	10	55	6	5
10/6 am	Epirubicin	10	45	5	5
10/6 am	Epirubicin	10	55	6	5
10/31 am	Epirubicin	10	55	6	5
10/30 am	Epirubicin	10	45	5	5
10/30 am	Epirubicin	10	55	6	5
10/29 am	Epirubicin	10	45	5	5
10/26 am	Epirubicin	10	45	5	5
10/26 am	Epirubicin	10	55	6	5
10/25 am	Epirubicin	10	45	5	5
10/18 am	Epirubicin	10	55	6	5
10/18 am	Epirubicin	10	55	6	5
10/18 am	Epirubicin	10	55	6	5
10/16 am	Epirubicin	10	55	6	5
10/14 am	Epirubicin	10	65	7	5
10/13 am	Epirubicin	10	65	7	5
10/12 am	Epirubicin	10	35	4	5
10/11 am	Epirubicin	10	45	5	5
				306	255
9/19 am	Bleomycin	15	20	2	10
9/18 am	Bleomycin	15	20	2	10
9/17 am	Bleomycin	15	20	2	10
11/12 am	Bleomycin	15	20	2	10
10/18 am	Bleomycin	15	20	2	10
10/16 pm	Bleomycin	15	20	2	10
10/15 am	Bleomycin	15	20	2	10
				14	70
9/27 am	Doxorubic	10	25	3	5
11/8 pm	Doxorubic	10	15	2	5
10/29 am	Doxorubic	10	39	4	1
10/1 am	Doxorubic	10	39	4	1
10/13 pm	Doxorubic	10	15	2	5
				15	17
9/9 am	Gemcitabi	0.2	0.35	2	50
9/8 am	Gemcitabi	0.2	0.5	3	100
9/2 am	Gemcitabi	0.2	0.35	2	50
9/25 am	Gemcitabi	0.2	0.5	3	100
9/23 pm	Gemcitabi	0.2	1.1	6	100
9/23 am	Gemcitabi	0.2	0.44	3	160
9/11 am	Gemcitabi	0.2	0.5	3	100
9/11 am	Gemcitabi	0.2	0.7	4	100
11/6 am	Gemcitabi	0.2	0.5	3	100
11/5 am	Gemcitabi	0.2	0.5	3	100
11/18 am	Gemcitabi	0.2	0.5	3	100
11/15 pm	Gemcitabi	0.2	2.05	11	150
11/14 am	Gemcitabi	0.2	0.44	3	160
11/12 am	Gemcitabi	0.2	0.5	3	100
10/8 am	Gemcitabi	0.2	0.3	2	100
10/5 am	Gemcitabi	0.2	0.5	3	100
10/30 am	Gemcitabi	0.2	0.3	2	100

10/2 am	Gemcitabi	0.2	0.5	3	100
10/2 am	Gemcitabi	0.2	0.5	3	100
10/23 am	Gemcitabi	0.2	0.5	3	100
10/1 am	Gemcitabi	0.2	0.3	2	100
10/16 am	Gemcitabi	0.2	0.5	3	100
10/15 am	Gemcitabi	0.2	0.5	3	100
10/14 am	Gemcitabi	0.2	0.7	4	100
10/12 am	Gemcitabi	0.2	0.5	3	100
				83	2570
9/7 pm	Gemcitabi	1	1.35	2	650
9/6 am	Gemcitabi	1	1.77	2	230
9/29 pm	Gemcitabi	1	1.77	2	230
9/29 am	Gemcitabi	1	1.89	2	110
9/29 am	Gemcitabi	1	1.6	2	400
9/28 pm	Gemcitabi	1	1.35	2	650
9/28 am	Gemcitabi	1	1.58	2	420
9/24 am	Gemcitabi	1	1.89	2	110
9/22 am	Gemcitabi	1	1.63	2	370
9/1 pm	Gemcitabi	1	1.6	2	400
9/14 am	Gemcitabi	1	1.63	2	370
9/13 pm	Gemcitabi	1	1.5	2	500
9/13 am	Gemcitabi	1	1.77	2	230
11/9 pm	Gemcitabi	1	0.7	1	300
11/9 pm	Gemcitabi	1	1.5	2	500
11/8 am	Gemcitabi	1	2.05	3	950
11/6 am	Gemcitabi	1	1.47	2	530
11/5 am	Gemcitabi	1	1.47	2	530
11/4 am	Gemcitabi	1	1.47	2	530
11/4 am	Gemcitabi	1	1.7	2	300
11/2 am	Gemcitabi	1	1.63	2	370
11/25 pm	Gemcitabi	1	1.63	2	370
11/24 pm	Gemcitabi	1	1.47	2	530
11/16 pm	Gemcitabi	1	1.63	2	370
11/15 pm	Gemcitabi	1	1.87	2	130
11/12 pm	Gemcitabi	1	1.5	2	500
11/11 pm	Gemcitabi	1	1.77	2	230
11/11 am	Gemcitabi	1	1.47	2	530
11/11 am	Gemcitabi	1	1.7	2	300
10/8 pm	Gemcitabi	1	1.77	2	230
10/30 am	Gemcitabi	1	1.77	2	230
10/29 am	Gemcitabi	1	1.47	2	530
10/27 pm	Gemcitabi	1	1.6	2	400
10/26 am	Gemcitabi	1	1.63	2	370
10/25 am	Gemcitabi	1	2.05	3	950
10/22 pm	Gemcitabi	1	1.5	2	500
10/22 am	Gemcitabi	1	1.49	2	510
10/21 am	Gemcitabi	1	1.77	2	230
10/21 am	Gemcitabi	1	1.49	2	510
10/19 am	Gemcitabi	1	1.35	2	650
10/18 am	Gemcitabi	1	2.05	3	950
10/15 pm	Gemcitabi	1	1.89	2	110

10/14 pm	Gemcitabi	1	2.4	3	600
10/14 pm	Gemcitabi	1	1.49	2	510
10/13 pm	Gemcitabi	1	0.7	1	300
				92	19220
9/9 am	Rubidomy	20	18.6	1	1.4
9/8 am	Rubidomy	20	25	2	15
9/4 am	Rubidomy	20	27	2	13
9/30 pm	Rubidomy	20	31	2	9
9/2 pm	Rubidomy	20	18.6	1	1.4
9/2 am	Rubidomy	20	29	2	11
9/24 am	Rubidomy	20	17	1	3
9/23 am	Rubidomy	20	30	2	10
9/20 am	Rubidomy	20	30	2	10
9/20 am	Rubidomy	40	30	2	50
9/1 am	Rubidomy	20	25	2	15
11/6 am	Rubidomy	20	17	1	3
11/5 am	Rubidomy	20	22.5	2	17.5
11/4 am	Rubidomy	20	30	2	10
11/27 am	Rubidomy	20	16	1	4
11/27 am	Rubidomy	20	17	1	3
11/26 am	Rubidomy	20	22.5	2	17.5
11/20 am	Rubidomy	20	16	1	4
11/19 am	Rubidomy	20	22.5	2	17.5
11/16 am	Rubidomy	20	15	1	5
11/12 am	Rubidomy	20	22.5	2	17.5
10/7 am	Rubidomy	20	24	2	16
10/7 am	Rubidomy	20	45	3	15
10/5 pm	Rubidomy	20	45	3	15
10/3 pm	Rubidomy	20	45	3	15
10/3 am	Rubidomy	20	45	3	15
10/27 am	Rubidomy	20	24	2	16
10/25 pm	Rubidomy	20	15	1	5
10/21 am	Rubidomy	20	45	3	15
10/1 pm	Rubidomy	20	31	2	9
10/1 pm	Rubidomy	20	45	3	15
10/15 pm	Rubidomy	20	17	1	3
10/14 pm	Rubidomy	20	30	2	10
10/14 am	Rubidomy	20	24	2	16
				64	402.8
9/8 am	Irinotecan	40	270	7	10
9/8 am	Irinotecan	40	290	8	30
9/5 am	Irinotecan	40	300	8	20
9/4 am	Irinotecan	40	290	8	30
9/3 am	Irinotecan	40	300	8	20
9/3 am	Irinotecan	40	300	8	20
9/3 am	Irinotecan	40	300	8	20
9/30 am	Irinotecan	40	270	7	10
9/28 am	Irinotecan	40	290	8	30
9/23 am	Irinotecan	40	290	8	30
9/22 am	Irinotecan	40	110	3	10
9/20 am	Irinotecan	40	290	8	30

9/20 am	Irinotecan	40	300	8	20
9/18 am	Irinotecan	40	350	9	10
9/18 am	Irinotecan	40	270	7	10
9/15 am	Irinotecan	40	270	7	10
11/5 am	Irinotecan	40	250	7	30
11/2 am	Irinotecan	40	270	7	10
11/29 pm	Irinotecan	40	30	1	10
11/26 am	Irinotecan	40	270	7	10
11/26 am	Irinotecan	40	300	8	20
11/25 am	Irinotecan	40	270	7	10
11/24 am	Irinotecan	40	290	8	30
11/21 am	Irinotecan	40	300	8	20
11/20 am	Irinotecan	40	278	7	2
11/1 am	Irinotecan	40	300	8	20
11/14 am	Irinotecan	40	60	2	20
11/13 am	Irinotecan	40	60	2	20
11/13 am	Irinotecan	40	230	6	10
11/12 am	Irinotecan	40	60	2	20
11/10 am	Irinotecan	40	270	7	10
10/7 pm	Irinotecan	40	290	8	30
10/4 am	Irinotecan	40	270	7	10
10/29 am	Irinotecan	40	270	7	10
10/28 am	Irinotecan	40	270	7	10
10/26 am	Irinotecan	40	270	7	10
10/23 am	Irinotecan	40	290	8	30
10/22 am	Irinotecan	40	20	1	20
10/22 am	Irinotecan	40	290	8	30
10/19 am	Irinotecan	40	270	7	10
10/19 am	Irinotecan	40	350	9	10
10/19 am	Irinotecan	40	278	7	2
10/15 am	Irinotecan	40	300	8	20
10/12 am	Irinotecan	40	270	7	10
				298	754
9/23 am	Ifosfamide	1	3.3	4	700
9/22 am	Ifosfamide	1	3.3	4	700
9/20 am	Ifosfamide	1	3.3	4	700
9/1 am	Ifosfamide	1	1.6	2	400
9/1 am	Ifosfamide	1	1.2	2	800
9/19 am	Ifosfamide	1	1.6	2	400
9/19 am	Ifosfamide	1	3.3	4	700
9/18 am	Ifosfamide	1	1.6	2	400
9/17 am	Ifosfamide	1	1.6	2	400
9/16 am	Ifosfamide	1	1.6	2	400
11/9 pm	Ifosfamide	1	1.9	2	100
11/9 pm	Ifosfamide	1	1.9	2	100
11/5 am	Ifosfamide	1	1.6	2	400
11/4 am	Ifosfamide	1	1.6	2	400
11/4 am	Ifosfamide	1	3.6	4	400
11/30 am	Ifosfamide	1	0.8	1	200
11/2 am	Ifosfamide	1	1.6	2	400
11/29 pm	Ifosfamide	1	0.8	1	200

11/28 pm	Ifosfamide	1	0.8	1	200
11/27 pm	Ifosfamide	1	0.8	1	200
11/26 pm	Ifosfamide	1	0.8	1	200
11/21 am	Ifosfamide	1	1.5	2	500
11/21 am	Ifosfamide	1	1.5	2	500
11/1 am	Ifosfamide	1	1.6	2	400
10/8 am	Ifosfamide	1	1.6	2	400
10/7 am	Ifosfamide	1	1.6	2	400
10/6 am	Ifosfamide	1	1.6	2	400
10/5 am	Ifosfamide	1	1.6	2	400
10/4 am	Ifosfamide	1	1.6	2	400
10/28 am	Ifosfamide	1	0.6	1	400
10/28 am	Ifosfamide	1	3.3	4	700
10/27 am	Ifosfamide	1	0.6	1	400
10/27 am	Ifosfamide	1	3.3	4	700
10/26 am	Ifosfamide	1	0.6	1	400
10/26 am	Ifosfamide	1	3.3	4	700
10/25 pm	Ifosfamide	1	2.5	3	500
10/25 am	Ifosfamide	1	3.5	4	500
10/25 am	Ifosfamide	1	0.6	1	400
10/25 am	Ifosfamide	1	3.3	4	700
10/24 am	Ifosfamide	1	0.6	1	400
10/24 am	Ifosfamide	1	3.5	4	500
10/23 am	Ifosfamide	1	3.5	4	500
10/22 pm	Ifosfamide	1	3.3	4	700
10/22 am	Ifosfamide	1	1.8	2	200
10/22 am	Ifosfamide	1	3.5	4	500
10/21 am	Ifosfamide	1	3.5	4	500
10/21 am	Ifosfamide	1	1.8	2	200
10/20 am	Ifosfamide	1	3.3	4	700
10/20 am	Ifosfamide	1	3.5	4	500
10/19 am	Ifosfamide	1	3.3	4	700
				127	22600
9/8 am	Ifosfamide	0.5	1.03	3	470
9/6 am	Ifosfamide	0.5	1.03	3	470
9/5 am	Ifosfamide	0.5	3.3	7	200
9/4 am	Ifosfamide	0.5	3.3	7	200
9/3 pm	Ifosfamide	0.5	0.72	2	280
9/3 am	Ifosfamide	0.5	0.72	2	280
9/3 am	Ifosfamide	0.5	3.3	7	200
9/2 pm	Ifosfamide	0.5	0.72	2	280
9/2 am	Ifosfamide	0.5	0.72	2	280
9/2 am	Ifosfamide	0.5	3.3	7	200
9/27 am	Ifosfamide	0.5	1.1	3	400
9/27 am	Ifosfamide	0.5	1.3	3	200
9/26 am	Ifosfamide	0.5	1.1	3	400
9/26 am	Ifosfamide	0.5	1.3	3	200
9/25 am	Ifosfamide	0.5	2.4	5	100
9/25 am	Ifosfamide	0.5	1.3	3	200
9/24 am	Ifosfamide	0.5	1.3	3	200
9/23 am	Ifosfamide	0.5	1.3	3	200

9/21 am	Ifosfamide	0.5	3.3	7	200
9/1 pm	Ifosfamide	0.5	0.72	2	280
9/1 am	Ifosfamide	0.5	3.3	7	200
9/19 pm	Ifosfamide	0.5	0.8	2	200
9/19 am	Ifosfamide	0.5	0.8	2	200
9/17 pm	Ifosfamide	0.5	0.8	2	200
9/13 am	Ifosfamide	0.5	2.2	5	300
9/12 am	Ifosfamide	0.5	2.2	5	300
9/11 am	Ifosfamide	0.5	2.2	5	300
9/07（9/7 a	Ifosfamide	0.5	1.03	3	470
9/05（9/5 a	Ifosfamide	0.5	1.03	3	470
9/04（9/4 a	Ifosfamide	0.5	1.03	3	470
11/5 am	Ifosfamide	0.5	3.3	7	200
11/4 am	Ifosfamide	0.5	3.3	7	200
11/4 am	Ifosfamide	0.5	2.4	5	100
11/3 am	Ifosfamide	0.5	3.3	7	200
11/30 pm	Ifosfamide	0.5	2.4	5	100
11/2 am	Ifosfamide	0.5	3.3	7	200
11/20 am	Ifosfamide	0.5	3.3	7	200
11/20 am	Ifosfamide	0.5	1.26	3	240
11/20 am	Ifosfamide	0.5	1.3	3	200
11/1 am	Ifosfamide	0.5	3.3	7	200
11/19 am	Ifosfamide	0.5	3.3	7	200
11/19 am	Ifosfamide	0.5	1.26	3	240
11/19 am	Ifosfamide	0.5	1.3	3	200
11/18 am	Ifosfamide	0.5	3.3	7	200
11/18 am	Ifosfamide	0.5	1.26	3	240
11/18 am	Ifosfamide	0.5	1.3	3	200
11/17 am	Ifosfamide	0.5	3.3	7	200
11/17 am	Ifosfamide	0.5	1.26	3	240
11/17 am	Ifosfamide	0.5	1.3	3	200
11/16 pm	Ifosfamide	0.5	1.3	3	200
11/16 am	Ifosfamide	0.5	3.3	7	200
11/16 am	Ifosfamide	0.5	1.26	3	240
11/15 am	Ifosfamide	0.5	1.7	4	300
11/14 am	Ifosfamide	0.5	1.7	4	300
11/13 am	Ifosfamide	0.5	1.7	4	300
10/9 am	Ifosfamide	0.5	3.3	7	200
10/8 am	Ifosfamide	0.5	3.3	7	200
10/7 am	Ifosfamide	0.5	3.3	7	200
10/6 am	Ifosfamide	0.5	3.3	7	200
10/1 am	Ifosfamide	0.5	2.4	5	100
10/18 am	Ifosfamide	0.5	3.3	7	200
10/18 am	Ifosfamide	0.5	3.3	7	200
10/16 am	Ifosfamide	0.5	3.3	7	200
10/15 pm	Ifosfamide	0.5	2.4	5	100
10/14 am	Ifosfamide	0.5	2.2	5	300
10/13 am	Ifosfamide	0.5	2.2	5	300
10/10 am	Ifosfamide	0.5	3.3	7	200
				312	16050
11/8 pm	Dextrazox	250	150	1	100

11/13 am	Dextrazox:	250	740	3	10
10/27 am	Dextrazox:	250	240	1	10
10/14 pm	Dextrazox:	250	240	1	10
10/13 pm	Dextrazox:	250	150	1	100
				7	230
9/9 am	Paclitaxel	100	370	4	30
9/9 am	Paclitaxel	100	450	5	50
9/9 am	Paclitaxel	100	390	4	10
9/9 am	Paclitaxel	100	450	5	50
9/8 am	Paclitaxel	100	460	5	40
9/7 am	Paclitaxel	100	360	4	40
9/7 am	Paclitaxel	100	360	4	40
9/7 am	Paclitaxel	100	230	3	70
9/7 am	Paclitaxel	100	390	4	10
9/4 am	Paclitaxel	100	390	4	10
9/4 am	Paclitaxel	100	450	5	50
9/30 am	Paclitaxel	100	350	4	50
9/30 am	Paclitaxel	100	470	5	30
9/30 am	Paclitaxel	100	350	4	50
9/2 am	Paclitaxel	100	180	2	20
9/2 am	Paclitaxel	100	380	4	20
9/2 am	Paclitaxel	100	450	5	50
9/29 am	Paclitaxel	100	470	5	30
9/29 am	Paclitaxel	100	380	4	20
9/29 am	Paclitaxel	100	360	4	40
9/28 am	Paclitaxel	100	370	4	30
9/28 am	Paclitaxel	100	360	4	40
9/27 am	Paclitaxel	100	395	4	5
9/26 am	Paclitaxel	100	439	5	61
9/24 am	Paclitaxel	100	350	4	50
9/24 am	Paclitaxel	100	480	5	20
9/24 am	Paclitaxel	100	390	4	10
9/23 am	Paclitaxel	100	390	4	10
9/23 am	Paclitaxel	100	380	4	20
9/23 am	Paclitaxel	100	180	2	20
9/23 am	Paclitaxel	100	350	4	50
9/23 am	Paclitaxel	100	390	4	10
9/23 am	Paclitaxel	100	450	5	50
9/20 pm	Paclitaxel	100	360	4	40
9/20 am	Paclitaxel	100	390	4	10
9/1 am	Paclitaxel	100	480	5	20
9/1 am	Paclitaxel	100	350	4	50
9/1 am	Paclitaxel	100	350	4	50
9/1 am	Paclitaxel	100	390	4	10
9/19 am	Paclitaxel	100	460	5	40
9/19 am	Paclitaxel	100	450	5	50
9/19 am	Paclitaxel	100	470	5	30
9/19 am	Paclitaxel	100	480	5	20
9/19 am	Paclitaxel	100	470	5	30
9/18 am	Paclitaxel	100	410	5	90
9/18 am	Paclitaxel	100	440	5	60

9/18 am	Paclitaxel	100	450	5	50
9/17 am	Paclitaxel	100	180	2	20
9/17 am	Paclitaxel	100	350	4	50
9/16 am	Paclitaxel	100	480	5	20
9/16 am	Paclitaxel	100	370	4	30
9/15 pm	Paclitaxel	100	350	4	50
9/15 am	Paclitaxel	100	196	2	4
9/15 am	Paclitaxel	100	480	5	20
9/14 am	Paclitaxel	100	360	4	40
9/14 am	Paclitaxel	100	230	3	70
9/14 am	Paclitaxel	100	350	4	50
9/11 pm	Paclitaxel	100	360	4	40
9/11 am	Paclitaxel	100	370	4	30
9/11 am	Paclitaxel	100	350	4	50
9/10 am	Paclitaxel	100	370	4	30
9/10 am	Paclitaxel	100	350	4	50
11/9 am	Paclitaxel	100	350	4	50
11/9 am	Paclitaxel	100	360	4	40
11/8 pm	Paclitaxel	100	380	4	20
11/8 pm	Paclitaxel	100	380	4	20
11/8 pm	Paclitaxel	100	330	4	70
11/8 am	Paclitaxel	100	380	4	20
11/8 am	Paclitaxel	100	390	4	10
11/8 am	Paclitaxel	100	188	2	12
11/7 pm	Paclitaxel	100	350	4	50
11/7 am	Paclitaxel	100	390	4	10
11/6 pm	Paclitaxel	100	380	4	20
11/6 am	Paclitaxel	100	380	4	20
11/6 am	Paclitaxel	100	390	4	10
11/6 am	Paclitaxel	100	450	5	50
11/5 am	Paclitaxel	100	320	4	80
11/4 am	Paclitaxel	100	480	5	20
11/4 am	Paclitaxel	100	180	2	20
11/4 am	Paclitaxel	100	360	4	40
11/4 am	Paclitaxel	100	350	4	50
11/4 am	Paclitaxel	100	350	4	50
11/30 am	Paclitaxel	100	370	4	30
11/29 pm	Paclitaxel	100	380	4	20
11/29 pm	Paclitaxel	100	380	4	20
11/29 pm	Paclitaxel	100	380	4	20
11/29 am	Paclitaxel	100	480	5	20
11/29 am	Paclitaxel	100	350	4	50
11/27 am	Paclitaxel	100	480	5	20
11/26 am	Paclitaxel	100	170	2	30
11/26 am	Paclitaxel	100	150	2	50
11/26 am	Paclitaxel	100	450	5	50
11/25 am	Paclitaxel	100	350	4	50
11/24 am	Paclitaxel	100	450	5	50
11/24 am	Paclitaxel	100	380	4	20
11/23 am	Paclitaxel	100	380	4	20
11/22 pm	Paclitaxel	100	350	4	50

11/22 am	Paclitaxel	100	350	4	50
11/21 am	Paclitaxel	100	350	4	50
11/20 am	Paclitaxel	100	460	5	40
11/1 am	Paclitaxel	100	430	5	70
11/1 am	Paclitaxel	100	150	2	50
11/19 pm	Paclitaxel	100	350	4	50
11/19 pm	Paclitaxel	100	490	5	10
11/19 pm	Paclitaxel	100	350	4	50
11/19 am	Paclitaxel	100	450	5	50
11/18 am	Paclitaxel	100	350	4	50
11/17 am	Paclitaxel	100	380	4	20
11/17 am	Paclitaxel	100	380	4	20
11/17 am	Paclitaxel	100	480	5	20
11/16 pm	Paclitaxel	100	450	5	50
11/16 pm	Paclitaxel	100	390	4	10
11/16 pm	Paclitaxel	100	150	2	50
11/16 am	Paclitaxel	100	180	2	20
11/16 am	Paclitaxel	100	470	5	30
11/15 pm	Paclitaxel	100	370	4	30
11/14 am	Paclitaxel	100	180	2	20
11/12 am	Paclitaxel	100	360	4	40
11/12 am	Paclitaxel	100	395	4	5
11/12 am	Paclitaxel	100	350	4	50
11/12 am	Paclitaxel	100	380	4	20
11/11 pm	Paclitaxel	100	380	4	20
11/11 pm	Paclitaxel	100	370	4	30
11/11 am	Paclitaxel	100	440	5	60
11/11 am	Paclitaxel	100	364	4	36
11/10 am	Paclitaxel	100	350	4	50
11/10 am	Paclitaxel	100	350	4	50
10/7 am	Paclitaxel	100	480	5	20
10/6 pm	Paclitaxel	100	380	4	20
10/31 pm	Paclitaxel	100	360	4	40
10/31 am	Paclitaxel	100	390	4	10
10/31 am	Paclitaxel	100	188	2	12
10/30 pm	Paclitaxel	100	490	5	10
10/30 pm	Paclitaxel	100	380	4	20
10/30 am	Paclitaxel	100	350	4	50
10/30 am	Paclitaxel	100	390	4	10
10/29 pm	Paclitaxel	100	170	2	30
10/29 am	Paclitaxel	100	450	5	50
10/29 am	Paclitaxel	100	450	5	50
10/29 am	Paclitaxel	100	350	4	50
10/27 pm	Paclitaxel	100	380	4	20
10/27 am	Paclitaxel	100	380	4	20
10/26 pm	Paclitaxel	100	350	4	50
10/26 am	Paclitaxel	100	270	3	30
10/26 am	Paclitaxel	100	370	4	30
10/25 am	Paclitaxel	100	350	4	50
10/24 am	Paclitaxel	100	67.5	1	32.5
10/23 am	Paclitaxel	100	390	4	10

10/23 am	Paclitaxel	100	360	4	40
10/23 am	Paclitaxel	100	360	4	40
10/22 am	Paclitaxel	100	395	4	5
10/22 am	Paclitaxel	100	380	4	20
10/21 pm	Paclitaxel	100	480	5	20
10/21 am	Paclitaxel	100	250	3	50
10/21 am	Paclitaxel	100	370	4	30
10/21 am	Paclitaxel	100	350	4	50
10/20 am	Paclitaxel	100	350	4	50
10/1 am	Paclitaxel	100	350	4	50
10/19 am	Paclitaxel	100	380	4	20
10/19 am	Paclitaxel	100	470	5	30
10/19 am	Paclitaxel	100	440	5	60
10/18 pm	Paclitaxel	100	380	4	20
10/18 am	Paclitaxel	100	350	4	50
10/18 am	Paclitaxel	100	350	4	50
10/16 am	Paclitaxel	100	390	4	10
10/16 am	Paclitaxel	100	480	5	20
10/16 am	Paclitaxel	100	460	5	40
10/14 pm	Paclitaxel	100	380	4	20
10/14 pm	Paclitaxel	100	390	4	10
10/14 am	Paclitaxel	100	440	5	60
10/14 am	Paclitaxel	100	480	5	20
10/12 am	Paclitaxel	100	483	5	17
10/11 am	Paclitaxel	100	390	4	10
10/11 am	Paclitaxel	100	390	4	10
				708	5869.5
9/6 am	Paclitaxel	30	200	7	10
9/27 am	Paclitaxel	30	220	8	20
9/19 am	Paclitaxel	30	230	8	10
9/16 am	Paclitaxel	30	230	8	10
11/8 am	Paclitaxel	30	75	3	15
11/4 pm	Paclitaxel	30	200	7	10
11/30 am	Paclitaxel	30	260	9	10
11/29 am	Paclitaxel	30	75	3	15
11/22 am	Paclitaxel	30	75	3	15
11/20 am	Paclitaxel	30	200	7	10
11/1 am	Paclitaxel	30	260	9	10
11/1 am	Paclitaxel	30	230	8	10
11/19 am	Paclitaxel	30	230	8	10
11/16 am	Paclitaxel	30	260	9	10
11/15 am	Paclitaxel	30	75	3	15
11/10 pm	Paclitaxel	30	140	5	10
11/10 am	Paclitaxel	30	220	8	20
10/22 am	Paclitaxel	30	220	8	20
10/16 am	Paclitaxel	30	230	8	10
10/11 am	Paclitaxel	30	230	8	10
				137	250
9/3 am	Calcium le	50	15	1	35
9/3 am	Calcium le	50	15	1	35
9/3 am	Calcium le	50	344	7	6

9/3 am	Calcium le	50	330	7	20
9/2 am	Calcium le	50	6.8	1	43.2
9/2 am	Calcium le	50	6.8	1	43.2
9/2 am	Calcium le	50	6.8	1	43.2
9/2 am	Calcium le	50	6.8	1	43.2
9/30 am	Calcium le	50	10	1	40
9/30 am	Calcium le	50	10	1	40
9/30 am	Calcium le	50	10	1	40
9/30 am	Calcium le	50	10	1	40
9/28 pm	Calcium le	50	10	1	40
9/28 pm	Calcium le	50	10	1	40
9/28 pm	Calcium le	50	10	1	40
9/28 pm	Calcium le	50	10	1	40
9/28 pm	Calcium le	50	20	1	30
9/25 am	Calcium le	50	15	1	35
9/25 am	Calcium le	50	15	1	35
9/24 am	Calcium le	50	15	1	35
9/20 am	Calcium le	50	330	7	20
9/17 pm	Calcium le	50	15	1	35
9/17 pm	Calcium le	50	15	1	35
9/16 am	Calcium le	50	15	1	35
9/16 am	Calcium le	50	15	1	35
9/15 am	Calcium le	50	15	1	35
9/15 am	Calcium le	50	15	1	35
9/14 am	Calcium le	50	15	1	35
9/14 am	Calcium le	50	15	1	35
11/30 am	Calcium le	50	15	1	35
11/30 am	Calcium le	50	15	1	35
11/29 pm	Calcium le	50	5	1	45
11/29 pm	Calcium le	50	5	1	45
11/29 am	Calcium le	50	15	1	35
11/29 am	Calcium le	50	15	1	35
11/29 am	Calcium le	50	15	1	35
11/28 am	Calcium le	50	15	1	35
11/28 am	Calcium le	50	15	1	35
11/27 pm	Calcium le	50	5	1	45
11/26 am	Calcium le	50	10	1	40
11/26 am	Calcium le	50	344	7	6
11/1 am	Calcium le	50	15	1	35
11/1 am	Calcium le	50	15	1	35
11/1 am	Calcium le	50	344	7	6
11/15 am	Calcium le	50	15	1	35
11/15 am	Calcium le	50	15	1	35
11/14 am	Calcium le	50	15	1	35
11/14 am	Calcium le	50	15	1	35
11/10 am	Calcium le	50	10	1	40
10/8 am	Calcium le	50	15	1	35
10/8 am	Calcium le	50	15	1	35
10/31 am	Calcium le	50	15	1	35
10/31 am	Calcium le	50	15	1	35
10/31 am	Calcium le	50	15	1	35

10/31 am	Calcium le	50	15	1	35
10/30 am	Calcium le	50	15	1	35
10/30 am	Calcium le	50	15	1	35
10/2 am	Calcium le	50	15	1	35
10/2 am	Calcium le	50	15	1	35
10/1 am	Calcium le	50	10	1	40
10/1 am	Calcium le	50	15	1	35
10/1 am	Calcium le	50	15	1	35
10/1 am	Calcium le	50	344	7	6
10/18 am	Calcium le	50	15	1	35
10/18 am	Calcium le	50	15	1	35
10/18 am	Calcium le	50	15	1	35
10/18 am	Calcium le	50	15	1	35
10/18 am	Calcium le	50	330	7	20
10/16 am	Calcium le	50	15	1	35
10/16 am	Calcium le	50	15	1	35
				112	2401.8
9/7 pm	Paclitaxel	30	200	7	10
9/7 pm	Paclitaxel	30	200	7	10
9/6 pm	Paclitaxel	30	230	8	10
9/5 pm	Paclitaxel	30	200	7	10
9/30 pm	Paclitaxel	30	200	7	10
9/2 pm	Paclitaxel	30	190	7	20
9/29 pm	Paclitaxel	30	220	8	20
9/29 pm	Paclitaxel	30	230	8	10
9/24 pm	Paclitaxel	30	190	7	20
9/24 pm	Paclitaxel	30	220	8	20
9/20 pm	Paclitaxel	30	220	8	20
9/1 pm	Paclitaxel	30	220	8	20
9/13 pm	Paclitaxel	30	220	8	20
9/11 pm	Paclitaxel	30	200	7	10
9/10 pm	Paclitaxel	30	230	8	10
11/30 pm	Paclitaxel	30	220	8	20
11/17 am	Paclitaxel	30	80	3	10
11/15 am	Paclitaxel	30	80	3	10
10/8 pm	Paclitaxel	30	160	6	20
10/6 am	Paclitaxel	30	220	8	20
10/31 am	Paclitaxel	30	220	8	20
10/26 am	Paclitaxel	30	258	9	12
10/14 pm	Paclitaxel	30	160	6	20
				164	352
				9464	314898.625
